# Maternal Diet Enrichment in *n*–3 Long-Chain Polyunsaturated Fatty Acids during Pregnancy and Lactation Improves Their Transfer from the Mother to the Offspring and Counterbalances High-Fat Diet-Induced Alterations in a Sex-Dependent Manner in Rats

**DOI:** 10.1016/j.cdnut.2026.109508

**Published:** 2026-08-07

**Authors:** Valentine S Moullé, Agnès David-Sochard, Blandine Castellano, Isabelle Grit, Alexis Gandon, Jean-Charles Martin, Patricia Parnet

**Affiliations:** 1 Nantes Université, INRAE, UMR 1280, PhAN, IMAD, F-44000 Nantes, France; 2 Centre Cardiovasculaire et Nutrition, INRAE, INSERM, Aix Marseille Université, Marseille, France

**Keywords:** perinatal, *n*–3 long-chain polyunsaturated fatty acid, milk, brain, metabolic programming

## Abstract

**Background:**

Low birth weight (LBW) increases the risk of developing metabolic disorders in adulthood and reduces fatty acid (FA) brain accretion in offspring in rodent models. As milk composition is partially influenced by maternal diet, it is conceivable to act on maternal diet to improve FA brain accretion and development in offspring.

**Objectives:**

The study aimed to evaluate the impact of *n*–3 long-chain polyunsaturated FA (LCPUFA) deficiency or enrichment in the maternal diet during gestation and lactation on maternal diet on milk composition, offspring brain lipids, and the potential to counterbalance the effects of a high-fat diet (HFD).

**Methods:**

We investigated the influence of *n*–3 LCPUFA-deficient and -enriched maternal diets, as well as HFD challenge, on key aspects of offspring development, including birth weight, milk and brain FA composition, glucose and lipid metabolism outcomes, and general behavior in male and female offspring from a rat model of maternal protein restriction.

**Results:**

Maternal *n*–3 LCPUFA–deficient diet leads to discernible alterations both in mother red blood cell (RBC) membranes, milk, and offspring brain, impacting critical FAs such as α-linolenic acid, eicosapentaenoic acid (EPA), and docosahexaenoic acid (DHA). The exact opposite effect is observed with a maternal EPA-, DHA-, and arachidonic acid (AA)–enriched diet. DHA, *n*–3 LCPUFA, and the *n*–6/*n*–3 ratio in maternal RBC membrane are identified as offspring-predicting brain FA composition. An *n*–3-deficient maternal diet clearly impacts metabolic health, in particular hepatic lipid metabolism, and general motor activity of the offspring later in life in a sex-dependent manner. Providing an EPA-DHA-AA–enriched diet during gestation and lactation partially normalized these parameters.

**Conclusions:**

Our findings demonstrate that maternal *n*–3 LCPUFA deficiency disrupts offspring brain FA composition and metabolic health, whereas an EPA-DHA-AA–enriched diet during gestation and lactation partially restores these alterations, highlighting the potential of maternal dietary intervention to mitigate long-term developmental and metabolic risks in LBW offspring, in particular after HFD challenge.

## Introduction

In humans, low birth weight (LBW), defined as <2500 g, increases the susceptibility to metabolic disorders in adulthood. This initial observation laid the foundation for the developmental origins of health and disease concept [1,2]. Any disparity between the amount of nutrients supplied by the placenta and the demand of the fetus leads to intrauterine growth restriction (IUGR). Furthermore, inadequate postnatal nutrition may contribute to extrauterine growth restriction (EUGR). Emerging evidence underscores the abnormal transfer of long-chain PUFAs (LCPUFAs) in IUGR infants [3], and the increased expression of specific fatty acid (FA) transporters in IUGR placentas does not necessarily correlate with heightened FA flow through the placenta to the fetus [4,5]. EUGR may result from inappropriate feeding, where insufficient quality milk is provided, which directly impacts brain development, as this is highly dependent on FA. Indeed, in a rat model of maternal protein restriction (PR), EUGR has been observed specifically in offspring born to and fed by PR mothers, in contrast to offspring weaned by control mothers, as previously reported in our laboratory [[6], [7], [8]]. Targeted and untargeted lipid analyses revealed that a low-protein diet affects milk lipid content more significantly than milk protein content [9,10]. We demonstrated that *n*–3 LCPUFAs are notably reduced in PR mothers, with very low levels of α-linolenic acid (ALA; C18:3 *n*–3), an essential FA sourced exclusively from food, as well as its derivatives, EPA (C20:5 *n*–3) and DHA (C22:6 *n*–3) [9].

Among LCPUFAs, arachidonic acid (AA; C20:4 *n*–6) and DHA are crucial for brain development because of their structural role in the brain as components of cell membranes [11]. They ensure membrane fluidity and contribute to health by limiting oxidative stress and inflammation [12]. An adequate supply through the mother’s diet is essential, as the conversion of their precursors, linoleic acid (LA; C18:2 *n*–6) and ALA, in the fetal liver and placenta is minimal [13,14].

Our dietary habits have undergone substantial changes, resulting in a significant increase in *n*–6 LCPUFA consumption through oils (LA) and animal products such as meat, eggs, and dairy (AA) [15]. In addition, because of their competition for the desaturase and elongase enzymes responsible for *n*–3 and *n*–6 LCPUFA biosynthesis, an excess of *n*–6 LCPUFA could downregulate DHA production. Consequently, insufficient *n*–3 LCPUFA supply may contribute to increased inflammation and impaired brain function, including deficits in learning and memory, as well as a heightened risk of mental disorders, which could exacerbate the burden associated with IUGR and EUGR.

Although breast milk is considered the most suitable and complete food for baby growth, evidence from randomized clinical trials in humans does not consistently demonstrate clear benefits of maternal *n*–3 LCPUFA supplementation on offspring neurodevelopment [16]. Supplementing with *n*–3 LCPUFA (DHA/EPA) during pregnancy reduces the risk of early preterm birth [16]. In children, reducing SFA and replacing it with PUFA or MUFA improves blood pressure and lipid levels. For other outcomes—such as overall pregnancy outcomes, infant growth, neurodevelopment, cardiovascular health, and allergies—the evidence is either inconsistent or shows no effect, so no clear conclusions can be made [17,18]. However, some studies have indicated the favorable effects of *n*–3 LCPUFA supplementation on specific domains of child development, and given the importance of this topic, the effects of *n*–3 LCPUFA deficiency and supplementation on cognitive development have been extensively studied in basic experimental research, with rodents offering greater experimental control over factors such as maternal or pup nutritional status [19].

Low levels of essential FA, including *n*–3 LCPUFAs, in the milk of PR mothers [9] prompted us to investigate the impact of a maternal low-protein diet, poor or enriched in *n*–3 LCPUFAs, on milk composition and offspring brain lipid profile. In the text, we referred to the diets as “*n*–3 reduced” or “*n*–3 enriched” without exhaustively listing every lipid species that differed between formulations. However, we provide a complete description of the diet composition, as well as comprehensive data on maternal and pup plasma lipid changes and milk composition in relation to the diets consumed by the dams.

To lay the groundwork for potential clinical application, we evaluated whether the lipid composition of mother red blood cell (RBC) membranes could serve as a reliable surrogate marker for lipid accumulation in both milk and the brains of offspring using a robust mathematical model. Finally, we assessed a range of physiological and behavioral parameters, such as postweaning growth, mobility, anxiety, and glucose and lipid metabolism, in both male and female offspring to determine the potential cumulative effects of *n*–3 insufficiency and maternal PR, as well as the extent to which these effects could be mitigated through dietary supplementation.

## Methods

### Ethics statement

All experiments were carried out in accordance with the guidelines of the National Legislation on Animal Care of the French Ministry of Research (Decree No 2013-118, 1 February, 2013) and with European Union (EU) legal frameworks relating to the protection of animals used for scientific purposes (i.e., Directive 2010/63/EU). They were approved by the Animal Ethics Committee of Pays de La Loire under reference APAFIS#13253-2020060814013364 v1. The scientists in charge of the experiments received training and personal authorization. The experiment was conducted at the experimental facilities at Nantes University (permit number E-44-015) delivered by French veterinary services on 18 April, 2019.

### Experimental diets

Diets were manufactured at INRAE SAAJ (Sciences de l’Animal et de l’Aliment de Jouy, Jouy-en-Josas, France), and their compositions based on the purified AIN-93G diet are provided in Supplemental Table 1. All diets contained a low-protein supply (8%) to induce a maternal PR during gestation and lactation. They also contained the same quantity of lipids (7%) but differed in their lipid composition. Different *n*–6/*n*–3 ratio in diets were obtained by using different oils. Rapeseed oil (Huilerie Bailly) was used to get an *n*–6/*n*–3 ratio equal to 2 and is referred to as the control diet (CTL group). Corn oil (Huilerie de Lapalisse) was used to get a high *n*–6/*n*–3 ratio equal to 50 and is referred to as an *n*–3-deficient diet (*n*–3 DEF group). A mix of rapeseed and coconut (C1758, Sigma) oils, Omégavie tuna oil 25 DHA triglyceride (TG) (1105-1-THONT 6, Polaris), and Arasco (50 1503, DSM Nutritional Products) was used to get a low *n*–6/*n*–3 ratio equal to 2 but with a directly available EPA, DHA, and AA, and is referred to as the EPA+DHA–enriched diet (EPA+DHA group). The FA composition of diets is provided in Supplemental Table 2.

### Animals

**FIGURE 1:**
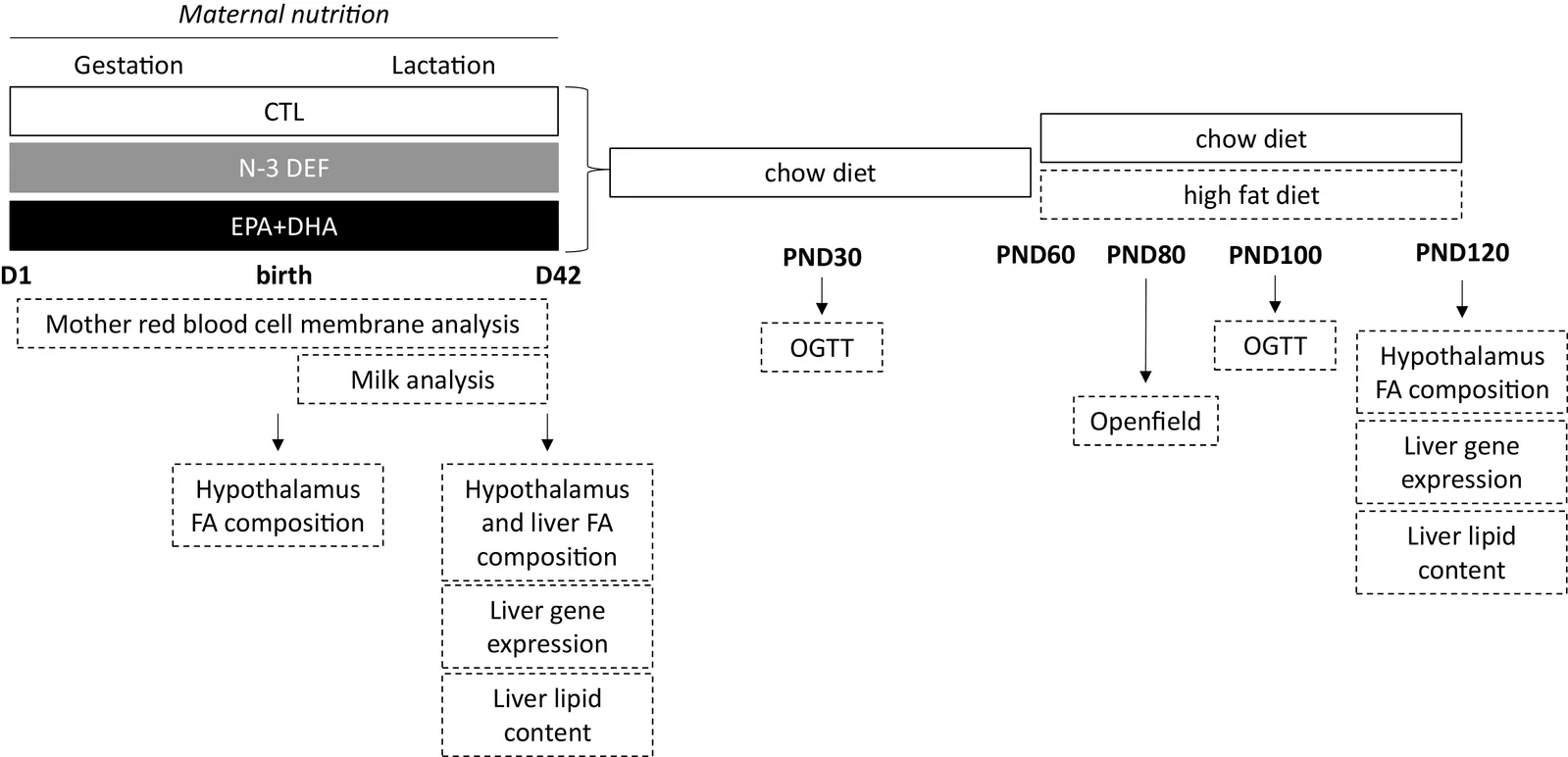
Schematic diagram of the study design. Three groups of animals were generated from mothers fed with a control diet (CTL; 8% proteins), a diet deficient in *n*–3 LCPUFA (*n*–3 DEF; 8% proteins), or a diet enriched in EPA, DHA, and AA (EPA+DHA; 8% proteins). Fatty acid (FA) composition was measured in mother red blood cell membrane and milk during gestation and lactation, and in the hypothalamus, prefrontal cortex, and liver in pups at birth, weaning, and postnatal day (PND) 120. At weaning, pups were fed an unpurified diet until PND60, where the diet was switched to a high-fat diet. The oral glucose tolerance test (OGTT) was performed at PND30 and PND100. LCPUFA, long-chain PUFA.

Pregnant Sprague–Dawley rats at gestation day (G) 1 were purchased from Janvier Labs. On arrival, rats were individually housed under controlled conditions (22°C, 12 h/12 h dark/light cycle, red tunnel, and Kraft paper curl for enrichment) and were randomly assigned to experimental diets during the gestation and lactation periods (Figure 1). Pregnant dams were weighed 3 times a week, and food and water intakes were measured at the same times. At birth, litter size was adjusted to 8 pups per dam (4 females and 4 males). Supernumerary animals were sacrificed, and the hypothalamus and liver were collected and immediately frozen in liquid nitrogen. Mothers and pups were weighed 3 times a week, and food and water intakes were measured at the same times. At weaning, PND22, 2 male and 2 female offspring and their mothers were anesthetized with isoflurane 4% and decapitated. Mesenteric WAT, perirenal WAT, subcutaneous WAT, and mammary gland from mothers were weighed. Hypothalamus and liver from offspring were weighed, collected, immediately frozen, and kept at –80°C until analysis. At PND60, half of the offspring were fed with an HFD (SAFE U8955 Version 2, SAFE) until the end of the experiments, that is, PND120, where hypothalamus and liver were collected, immediately frozen, and kept at –80°C. Animal cages were randomly located on the rack to limit confounders.

### Oral glucose tolerance test

Glucose (2 g/kg) was administered per os in 16-h fasted animals for the oral glucose tolerance test. Blood glucose was measured on a glucometer (ACCU-CHEK PERFORMA, Roche Diagnostics France) by a tail snip at 0, 15, 30, 45, 60, 90, and 120 min. Blood samples were taken at 0, 15, and 30 min. Tests were performed at PND30 and PND100.

### Locomotor activity and anxiety

Between PND80 and PND85, each animal was transferred to the open-field apparatus (50 × 50 cm) and was allowed to freely explore the new environment for 5 min. The experiment was performed in the light phase, without a visual cue, and between 09:00 and 12:00. The Viewpoint videotrack system (Point Grey Research Inc) allowed analyzing total distance traveled (cm) and time spent in the central area of the arena (%).

### Biological sampling collection

See Supplemental materials for further details.

### FA measurement in mother RBC membrane, maternal milk, and offspring brain and liver

Total lipids were extracted from 100 μL of maternal RBC membrane, 50 μL of milk, whole hypothalamus, whole prefrontal cortex, and 50 mg of liver using the modified liquid–liquid extraction method of Bligh and Dyer [20]. FAs were extracted with 200 μL methanol-chloroform (1:1, v/v). Hypothalamus, cortex, and liver were crushed with a zirconium marble in PreCellys (Bertin Instruments). Before extraction, sodium chloride 150 mM and 25 μL of internal standard heptadecanoic acid 10 mg/mL (C17:0; Sigma) were added. Two other extractions with 100 μL chloroform were performed, and the samples were then centrifuged (10,000 × *g*, 10 min) to recover the extracts. The organic phases were dried under N2. One milliliter of toluene was added to dissolve TGs. Total FAs were transesterified using 1 mL of trifluoride in methanol (BF3; Sigma France) for 45 min at 100°C. The FAs methyl esters were extracted by the addition of 0.25 mL of water and 0.50 mL of hexane and analyzed by GC using an Agilent Technologies 7890A instrument equipped with a splitless injector (20:1) and a fused silica capillary Elite 225 column (30 m × 0.32 mm, 0.25 μm, Perkin Elmer France). H2 was used as carrier gas at 2 mL/min. The column temperature program started at 50°C for 2 min, ramped at 15°C/min to 180°C for 2 min, and then ramped at 10°C/min to 220°C for 10 min. The temperature of the injector and the flame ionization detector was 250°C. Each FA was expressed as a percentage of the total identified FAs, and only FAs whose percentage was >0.1% were considered. The sum of FAs (in g/L) was assumed to represent total lipids (free FAs, triacylglycerols, and phospholipids) content.

### TG and cholesterol content in the liver

Around 50 mg of a piece of liver was crushed in saline with a zirconium marble in PreCellys. See Supplemental Materials for further details.

### Biochemical analysis

Assay methods are described in Supplemental Materials.

### qPCR

Total RNA was extracted from offspring liver using NucleoSpin RNA/Protein (Machery-Nagel) following the manufacturer’s instructions, and its content and purity were measured with a NanoVueTM spectrophotometer (Dutsher). RNA quality was assessed by evaluating the 230/260 and the 260/280 ratios during quantification. cDNA was synthesized from 1 μg of purified and denatured mRNA using the Superscript III reverse transcriptase (Thermo Fisher, 19080093) and diluted to a final concentration of 8 ng eq RNA/μL. qPCR was performed using StepOne Plus (Thermo Fisher) detection system with Fast SYBR Green (Thermo Fisher, 4385612) master mix. The PCR signal was normalized against beta-actin as a reference gene to control for variability in the amount and quality of the RNA. The sense and antisense oligonucleotide primers used in this study are shown in Supplemental Table 7.

### Expression of data and statistical analysis

Data are expressed as mean ± SEM and individual values. Statistical analyses were performed using Student’s t-test or analysis of variance followed by 2 × 2 comparisons using Tukey’s post hoc test (GraphPad Prism 7 version 7.0a, software). *P <* 0.05 was considered significant. Correlations were performed using R software (version 3.3.0+), RStudio software (version 2022.07.2+576), and a homemade script with functions cor(x,y) and cor.mtest(mat,…) to calculate correlations and *P* values, respectively. Only *r* > 0.6 with at least a *P* value < 0.05 was considered as correlated and shown on correlograms.

PLS regression analyses for brain DHA prediction were performed using SIMCA P12 (Umetrix). The optimal mother RBC FA predictors for hypothalamic DHA content in pups were derived through the analysis of paired mother–pup data, employing the most effective partial least squares (PLS) regression coefficients determined by the PLS algorithm. These coefficients were meticulously selected based on a normality plot of their values [21,22]. Subsequently, these carefully chosen mother RBC FAs were integrated into a linear equation. This involved multiplying each individual FA value by its corresponding PLS regression coefficient and then adding a constant, following a methodology outlined elsewhere [21].

## Results

### Diets composition

The diets (see also the Methods section) contained the same quantity of lipids (7%) but differed in their lipid composition (Supplemental Table 1). Different *n*–6/*n*–3 ratio in diets were obtained by using different oils: Rapeseed oil was used to get a *n*–6/*n*–3 ratio equal to 2 and is referred to as the control diet (CTL group). Corn oil was used to get a high *n*–6/*n*–3 ratio equal to 50 and is referred to as an *n*–3-deficient diet (*n*–3 DEF group). A mix of rapeseed and coconut oils, Omégavie tuna oil, and Arasco was used to get a low *n*–6/*n*–3 ratio equal to 2 but with directly available EPA, DHA, and AA, and is referred to as the EPA+DHA–enriched diet (EPA+DHA group) for nomenclature simplification. The complete FA composition of diets is provided in Supplemental Table 2.

### Maternal physiological outcomes do not vary according to diet

At the end of gestation and lactation, the body weight gain and cumulative food and water intake of the dams were similar between groups (Figure 1; Table 1). Although cumulative lipid intake was similar, consumption of specific FA classes diverged and aligned with the FA composition of the respective diets during gestation and lactation (Supplemental Tables 1 and 2). In consequence, animals in the *n*–3 DEF and EPA+DHA groups demonstrated higher SFA intake and lower MUFA intake (*P <* 0.001). LCPUFA intake was notably 2-fold higher in the *n*–3 DEF group (*P <* 0.001) and akin to the CTL group in the EPA+DHA group.

**TABLE 1:** Follow-up of mothers during gestation and lactation

	CTL	*n*–3 DEF	EPA+DHA	ANOVA
At the end of gestation				
Total BW gain from G1 (g)	177.1 ± 5.2	187.6 ± 6.1	187.6 ± 5.7	ns
Cumulative food intake (kcal/kg)	6617.6 ± 207.9	6713.0 ± 123.4	6583.7 ± 199.5	ns
Cumulative lipid intake (kcal/kg)	1042.3 ± 32.7	1057.3 ± 19.4	1036.9 ± 31.4	ns
Cumulative SFA intake (kcal/kg)	70.8 ± 2.2	137.5 ± 2.5∗∗∗	132.9 ± 4.0∗∗∗^,^##	*P <* 0.001
Cumulative MUFA intake (kcal/kg)	654.6 ± 20.6	278.2 ± 5.1∗∗∗	558.7 ± 16.9∗∗∗^,^###	*P <* 0.001
Cumulative LCPUFA intake (kcal/kg)	316.9 ± 10.0	641.6 ± 11.8∗∗∗	345.3 ± 10.5###	*P <* 0.001
Cumulative water intake (g/kg)	1145.5 ± 44.9	1101.3 ± 32.3	1128.5 ± 23.4	ns
At the end of lactation				
Total BW gain from L1 (g)	–19.7 ± 8.9	–21.6 ± 5.9	–31.6 ± 10.9	ns
Cumulative food intake (kcal/kg)	9174.2 ± 155.4	8970.1 ± 250.1	8579.8 ± 294.6	ns
Cumulative Lipid intake (kcal/kg)	1412.8 ± 39.4	1444.9 ± 24.5	1351.3 ± 46.4	ns
Cumulative SFA intake (kcal/kg)	91.3 ± 2.5	187.9 ± 3.2∗∗∗	161.3 ± 5.5∗∗∗^,^###	*P <* 0.001
Cumulative MUFA intake (kcal/kg)	844.4 ± 23.5	380.1 ± 6.4∗∗∗	677.9 ± 23.3∗∗∗^,^###	*P <* 0.001
Cumulative LCPUFA intake (kcal/kg)	408.8 ± 11.4	876.9 ± 14.8∗∗∗	419.0 ± 14.4###	*P <* 0.001
Cumulative water intake (g/kg)	1786.5 ± 48.6	1779.3 ± 60.5	1804.6 ± 96.1	ns

Values are mean ± SEM. *N =* 13–15 mothers per group. One-way ANOVA followed by the Tukey test.

Abbreviations: ANOVA, analysis of variance; BW, body weight; CTL, control diet; LCPUFA, long-chain PUFA; *n*–3 DEF, *n*–3 deficient diet; ns, not significant.

∗∗∗ P < 0.001 compared with CTL.

## P < 0.01

### P < 0.001 compared with *n*–3 DEF.

Plasma TG levels were significantly reduced in the EPA+DHA group during the 2nd and 3rd weeks of gestation (*P <* 0.01) with no difference in blood glucose, plasma insulin, leptin, and adiponectin levels, hepatic TG and cholesterol content, or the weights of mesenteric, perirenal white adipose tissue (WAT), mammary gland, and the liver between groups (Supplemental Figure 1).

### The FA composition of the maternal diet impacts offspring numbers and their birth weight

At birth, the number of pups from *n*–3 DEF mothers were significantly higher than in the CTL group, with a higher number of females (Table 2). Although birth mortality did not statistically differ between groups, the EPA+DHA group exhibited a higher incidence of stillbirths. Litter weight showed a tendency to increase in the *n*–3 DEF group (*P =* 0.07; 1-way analysis of variance), aligning with the higher number of pups. The male offspring in the *n*–3 DEF group exhibited smaller body weights at birth than the CTL group (*P <* 0.01), whereas the EPA+DHA–enriched diet prevented lower birth weights only in female offspring (*P <* 0.05).

**TABLE 2:** Parturition

	CTL	*n*–3 DEF	EPA+DHA	ANOVA
*At birth*				
Pup number	11.2 ± 0.8	13.9 ± 0.6∗	12.9 ± 0.5	*P <* 0.05
Male	5.4 ± 0.6	6.2 ± 0.7	6.1 ± 0.6	ns
Female	5.5 ± 0.6	7.6 ± 0.6∗	6.6 ± 0.4	*P <* 0.05
Mortality at birth (%)	1.1 ± 0.8	0.8 ± 0.8	4.2 ± 2.1	ns
Litter weight (g)	80.2 ± 4.6	93.2 ± 4.2	89.8 ± 2.8	*P =* 0.07
Pup weight (g)				
Male	7.3 ± 0.1	6.8 ± 0.1∗∗	7.1 ± 0.1∗	*P <* 0.01
Female	6.7 ± 0.1	6.4 ± 0.1	6.7 ± 0.1	*P =* 0.06

Values are mean ± SEM. *N =* 49–56 pups for 14–15 litters per group. One-way ANOVA followed by the Tukey test.

Abbreviations: ANOVA, analysis of variance; CTL, control diet; *n*–3 DEF, *n*–3 deficient diet; ns, not significant.

∗ *P <* 0.05

∗∗ *P <* 0.01 compared with CTL.

### The FA composition of the maternal diet significantly influences the lipid profile of mother RBC membranes

**FIGURE 2:**
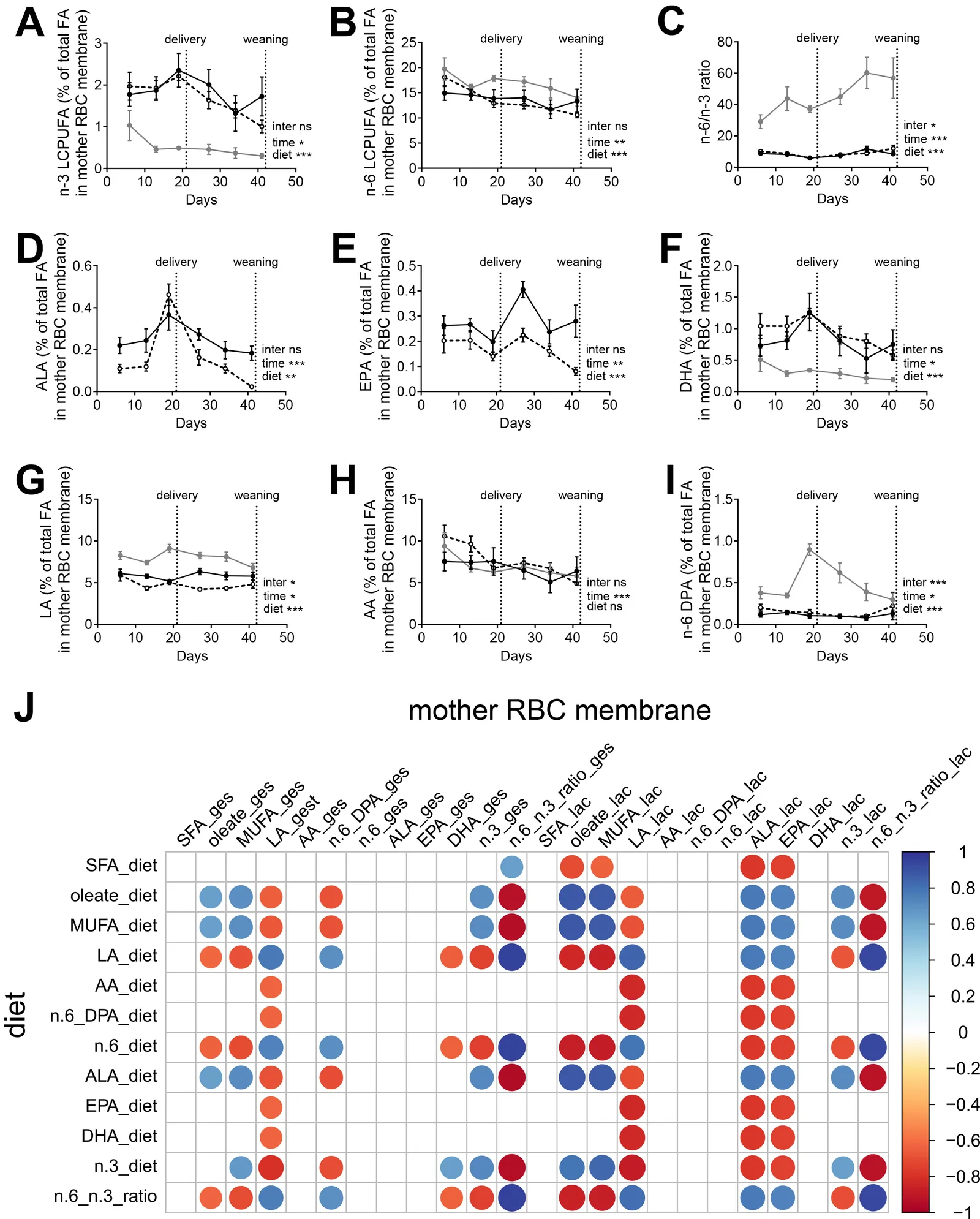
Evolution of selected fatty acid percentage in mother red blood cell membrane during gestation and lactation. Evolution of *n*–3 LCPUFA (A), *n*–6 LCPUFA (B), *n*–6/*n*–3 ratio (C), ALA (D), EPA (E), DHA (F), LA (G), AA (H), and *n*–6 DPA (I) in red blood cell membrane from maternal blood samples. Significant correlations >0.6 between selected fatty acids in the diet and maternal red blood cell membrane during gestation and lactation are presented in a correlation matrix (J). Solid line, CTL; gray line, *n*–3 DEF; Dotted black line, EPA+DHA. Vertical dotted line at D21 indicates delivery, and at D42 indicates weaning. Data are mean ± SEM (*n =* 3–11). *P <* 0.05, ∗∗*P <* 0.01, ∗∗∗*P <* 0.001, 2-way ANOVA with the Tukey post hoc test. AA, arachidonic acid; ALA, alpha-linolenic acid; ANOVA, analysis of variance; CTL, control diet; DPA, docosapentaenoic acid; EPA+DHA, EPA-, DHA-, and AA-enriched diet; LA, linoleic acid; LCPUFA, long-chain PUFA; *n*–3 DEF, *n*–3 deficient diet.

Both *n*–3 DEF and EPA+DHA–enriched diets induced marked changes in mother RBC FA composition (Table 3). Although total LCPUFA remained comparable across all groups, specific patterns emerged. *n*–3 LCPUFAs were 3 times lower in the *n*–3 DEF group (*P <* 0.001; Figure 2A), accompanied by a slight increase in *n*–6 LCPUFAs (Figure 2B), during both the gestation and lactation periods, leading to a significant rise in the *n*–6/*n*–3 ratio (*P <* 0.001; Figure 2C). ALA and EPA were undetectable in the RBC of the *n*–3 DEF group and reduced in the EPA+DHA group (Figure 2D, E). DHA levels were stable between the CTL and EPA+DHA groups but declined in the *n*–3 DEF group (*P <* 0.001; Figure 2F). LA increased significantly in the *n*–3 DEF group compared with the CTL group (*P <* 0.001) and decreased in the EPA+DHA group (Figure 2G). The AA percentage remained constant across groups during gestation and lactation (Figure 2H), whereas *n*–6 docosapentaenoic acid (DPA) significantly rose in *n*–3 DEF rats (*P <* 0.001; Figure 2I). These alterations were consistent with maternal dietary intake (Table 1; Supplemental Table 2).

**TABLE 3:** Percentage of selected fatty acids in maternal RBC membranes during gestation and lactation

	CTL	*n*–3 DEF	EPA+DHA	ANOVA	CTL	*n*–3 DEF	EPA+DHA	ANOVA
	*Gestation*				*Lactation*			
12:0	0.14 ± 0.02	0.14 ± 0.03	0.21 ± 0.02	*P =* 0.05	0.15 ± 0.01	0.17 ± 0.01	0.17 ± 0.1	ns
14:0	0.56 ± 0.02	0.57 ± 0.01	0.64 ± 0.02∗∗^,^##	<0.01	0.60 ± 0.01	0.57 ± 0.02	0.66 ± 0.02##	<0.01
15:0	0.28 ± 0.01	0.29 ± 0.01	0.32 ± 0.01∗	<0.05	0.28 ± 0.02	0.24 ± 0.01	0.32 ± 0.02#	<0.05
16:0	40.7 ± 0.7	43.2 ± 0.7	41.2 ± 0.9	ns	43.7 ± 1.2	45.1 ± 1.1	46.3 ± 0.4	ns
18:0	21.1 ± 0.3	22.1 ± 0.7	20.9 ± 0.4	ns	17.6 ± 0.4	19.5 ± 0.4∗	17.3 ± 0.4##	<0.01
20:0	0.22 ± 0.01	0.23 ± 0.01	0.21 ± 0.01	ns	0.211 ± 0.015	0.217 ± 0.002	0.197 ± 0.008	ns
22:0	0.65 ± 0.02	0.67 ± 0.02	0.64 ± 0.02	ns	0.65 ± 0.03	0.70 ± 0.03	0.67 ± 0.03	ns
24:0	2.0 ± 0.1	2.2 ± 0.1∗	2.0 ± 0.1	<0.05	2.0 ± 0.1	2.4 ± 0.1∗	2.2 ± 0.1	<0.05
**SCFA**	0.75 ± 0.05	0.75 ± 0.04	0.87 ± 0.07	ns	0.90 ± 0.07	0.89 ± 0.07	0.94 ± 0.01	ns
**MCFA**	62.2 ± 1.0	65.6 ± 1.2	62.4 ± 1.2	*P =* 0.07	61.6 ± 1.3	64.8 ± 1.0	63.9 ± 0.5	ns
**LCFA**	2.9 ± 0.1	3.1 ± 0.1∗	2.9 ± 0.1	<0.05	2.9 ± 0.1	3.3 ± 0.1∗	3.1 ± 0.1	<0.05
**SFA**	65.8 ± 1.0	69.6 ± 1.2	66.2 ± 1.2	*P =* 0.06	65.5 ± 1.4	69.1 ± 0.1∗	68.1 ± 0.4	<0.05
16:1 *n*–9	0.29 ± 0.01	0.20 ± 0.02∗∗	0.24 ± 0.01	<0.01	0.29 ± 0.01	0.20 ± 0.01∗∗∗	0.22 ± 0.02∗∗	<0.001
16:1 *n*–7	0.54 ± 0.05	0.41 ± 0.03	0.58 ± 0.04#	<0.05	0.93 ± 0.08	0.71 ± 0.03∗	0.81 ± 0.05	<0.05
18:1 *n*–9	10.5 ± 0.6	7.3 ± 0.4∗∗∗	9.7 ± 0.4##	<0.001	12.1 ± 0.3	8.0 ± 0.2∗∗∗	10.9 ± 0.4###	<0.001
20:1	0.22 ± 0.01	0.12 ± 0.01∗∗∗	0.19 ± 0.01∗^,^###	<0.001	0.24 ± 0.01	0.11 ± 0.00∗∗∗	0.19 ± 0.00∗^,^###	<0.001
22:1	1.1 ± 0.1	1.1 ± 0.1	1.1 ± 0.1	ns	1.1 ± 0.1	1.5 ± 0.2	1.3 ± 0.2	ns
24:1	1.44 ± 0.05	1.12 ± 0.04∗∗∗	1.41 ± 0.04###	<0.001	1.93 ± 0.06	1.30 ± 0.04∗∗∗	1.94 ± 0.06###	<0.001
**MUFA**	17.5 ± 0.7	13.0 ± 0.5∗∗∗	16.7 ± 0.6###	<0.001	20.1 ± 0.5	14.4 ± 0.3∗∗∗	19.0 ± 0.06###	<0.001
18:2 *n*–6 (LA)	5.9 ± 0.2	7.9 ± 0.4∗∗∗	4.8 ± 0.3∗^,^###	<0.001	6.0 ± 0.3	7.9 ± 0.3∗∗∗	4.3 ± 0.2∗∗∗^,^###	<0.001
20:2 *n*–6	0.23 ± 0.04	0.33 ± 0.04	0.17 ± 0.02#	<0.05	0.13 ± 0.01	0.16 ± 0.01	0.10 ± 0.01##	<0.01
20:3 *n*–6	0.34 ± 0.02	0.21 ± 0.02∗∗∗	0.28 ± 0.02#	<0.001	0.39 ± 0.03	0.31 ± 0.03	0.24 ± 0.01∗∗∗	<0.01
20:4 *n*–6 (AA)	7.6 ± 0.8	7.0 ± 0.4	9.1 ± 0.6	*P =* 0.06	5.8 ± 1.1	6.4 ± 0.7	6.3 ± 0.4	ns
22:4 *n*–6	0.68 ± 0.05	0.95 ± 0.06∗∗	0.80 ± 0.05	<0.01	0.4 ± 0.1	0.8 ± 0.1∗∗	0.4 ± 0.1##	<0.001
22:5 *n*–6 (DPA)	0.15 ± 0.02	0.46 ± 0.07∗∗∗	0.15 ± 0.02###	<0.001	0.08 ± 0.03	0.46 ± 0.07	0.25 ± 0.16	*P =* 0.06
**n-6 LCPUFA**	14.8 ± 1.0	16.8 ± 0.8	15.3 ± 0.8	ns	12.8 ± 1.5	16.1 ± 1.0	11.6 ± 0.5#	<0.05
18:3 *n*–3 (ALA)	0.22 ± 0.04	—	0.16 ± 0.03	**—**	0.22 ± 0.03	—	0.10 ± 0.01∗∗	—
20:5 *n*–3 (EPA)	0.24 ± 0.02	—	0.19 ± 0.03	—	0.31 ± 0.04	—	0.15 ± 0.01∗∗	**—**
22:5 *n*–3 (DPA)	0.54 ± 0.07	0.23 ± 0.03∗∗∗	0.53 ± 0.04###	<0.001	0.48 ± 0.09	0.15 ± 0.02∗∗	0.34 ± 0.02	<0.01
22:6 *n*–3 (DHA)	0.9 ± 0.1	0.3 ± 0.1∗∗	1.0 ± 0.1###	<0.001	0.66 ± 0.20	0.26 ± 0.05	0.75 ± 0.05#	<0.05
***n*–3 LCPUFA**	1.9 ± 0.2	0.6 ± 0.1∗∗∗	1.9 ± 0.2###	<0.001	1.6 ± 0.4	0.4 ± 0.1∗∗	1.3 ± 0.1##	<0.01
**Total LCPUFA**	16.7 ± 1.2	17.4 ± 0.9	17.1 ± 1.0	ns	14.5 ± 1.9	16.5 ± 1.1	13.0 ± 0.5	ns
***n*–6/*n*–3 ratio**	8.2 ± 0.3	32.9 ± 1.7∗∗∗	8.8 ± 0.6###	<0.001	9.2 ± 0.8	52.9 ± 5.5∗∗∗	9.5 ± 0.5###	<0.001

Values are mean ± SEM.

*N =* 3–11.

One-way ANOVA with the Tukey post hoc test, except for 18:3 *n*–3 (ALA) and 20:5 *n*–3 (EPA), where we performed a Student t-test.

Abbreviations: AA, arachidonic acid; ALA, alpha-linolenic acid; ANOVA, analysis of variance; CTL, control diet; DPA, docosapentaenoic acid; LA, linoleic acid; LCFA, long-chain SFA; LCPUFA, long-chain PUFA; MCFA, medium-chain SFA; *n*–3 DEF, *n*–3 deficient diet; ns, not significant; SCFA, short-chain saturated fatty acid.

∗ *P <* 0.05

∗∗ *P <* 0.01

∗∗∗ *P <* 0.001 compared with CTL.

# *P <* 0.05

## *P <* 0.01

### *P <* 0.001 compared with *n*–3 DEF.

Moreover, the mother RBC membrane composition was positively correlated with total MUFA, including oleate, LA, *n*–3 LCPUFAs, and *n*–6/*n*–3 ratio in the diet both during gestation and lactation (Figure 2J). LA was negatively correlated with *n*–3 LCPUFAs and positively correlated with the *n*–6/*n*–3 ratio. In contrast, ALA showed the opposite.

### The composition of maternal dietary fats also shapes the FA profile of the offspring’s brain at birth

**FIGURE 3:**
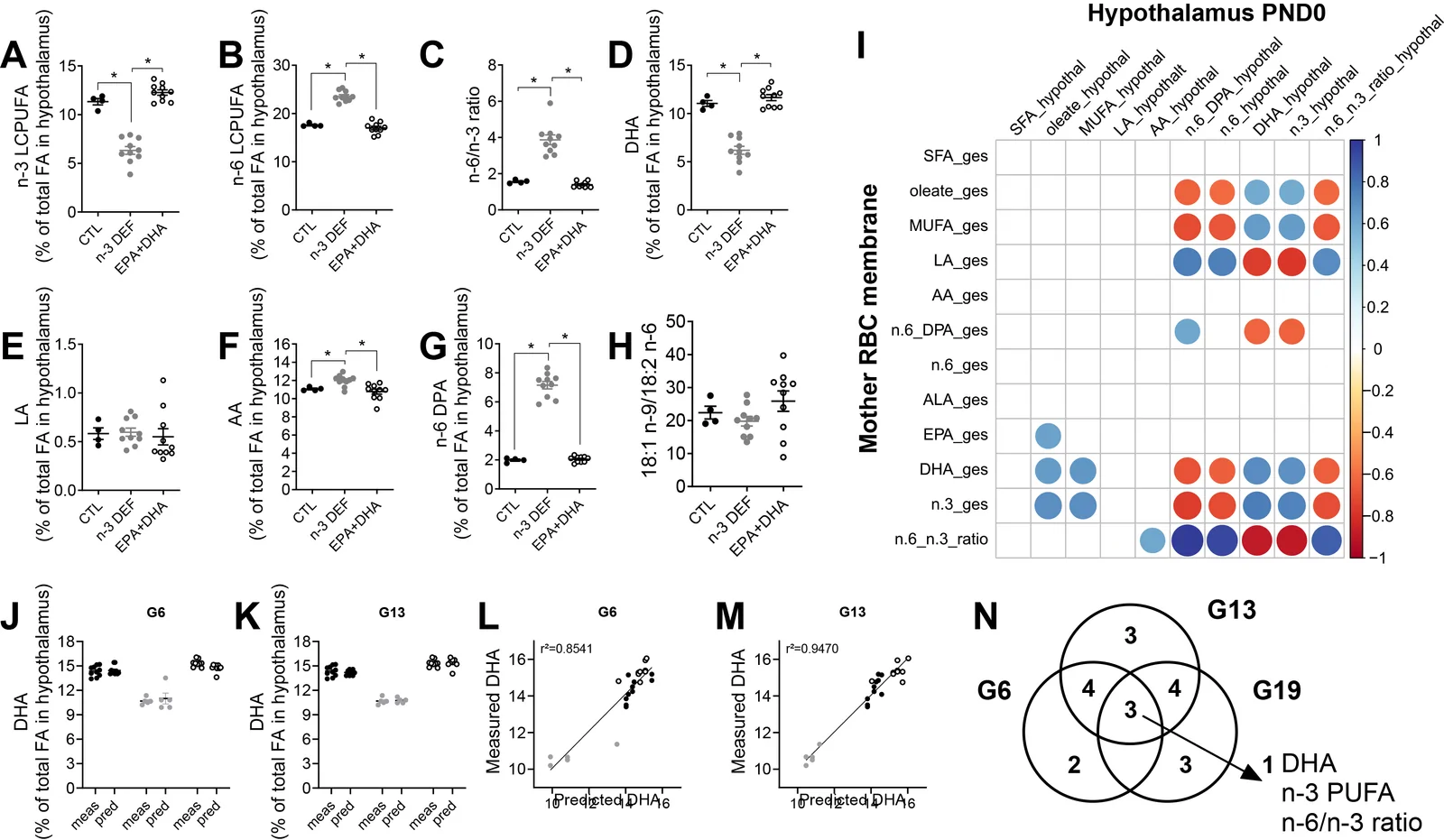
Evolution of selected FA percentage in offspring hypothalamus at birth and predicted DHA in offspring hypothalamus at PND21 from mother red blood cell (RBC) membrane FA composition. Evolution of *n*–3 LCPUFA (A), *n*–6 LCPUFA (B), *n*–6/*n*–3 ratio (C), DHA (D), LA (E), AA (F), *n*–6 DPA (G), and 18:1 *n*–9/18:2 *n*–6 ratio (H) in the hypothalamus of male and female offspring at birth. Significant correlations >0.6 between selected fatty acids in mother RBC membrane during gestation and the hypothalamus at birth are presented in a correlation matrix (I). Differences among measured and predicted DHA in offspring hypothalamus at G6 (J) and G13 (K), according to the predictive PLS algorithm. Correlations between measured and predicted DHA in the offspring hypothalamus at G6 (L) and G13 (M). The predictive equation calculated for the offspring hypothalamus from mother RBC fatty acids at G6 was as follows: [Predicted hypothalamic DHA = (2.39515 ∗ RBC C12:0 G6) – (10.8953 ∗ RBC C20:0 G6) + (2.6566 ∗ RBC C24:1 G6) – (4.86015 ∗ RBC C20:2*n*–6 G6) – (1.34763 ∗ RBC C22:4*n*–6 G6) – (3.23215 ∗ RBC C18:3*n*–3 G6) + (0.845823 ∗ RBC C22:5*n*–3 G6) + (0.392442 ∗ RBC C22:6*n*–3 G6) + (0.327801 ∗ RBC LCPUFA *n*–3 G6) – (0.0565429 ∗ RBC *n*–6/*n*–3_ratio G6) + 15.0918], and at G13 [Predicted hypothalamic DHA = (4.80692 ∗ RBC C15:0 G13) – (0.0750111 ∗ RBC C18:0 G13 – (5.00148 ∗ RBC C20:0 G13) + (0.845645 ∗ RBC C18:1*n*–7 G13) + (0.682158 ∗ RBC C24:1 G13) – (0.228451 ∗ RBC C18:2*n*–6 G13) + (1.50614 ∗ RBC C20:2*n*–6 G13) + (0.0172122 ∗ RBC C20:4*n*–6 G13) – (2.42756 ∗ RBC C22:5*n*–6 G13) + (1.31347∗ RBC C22_5_*n*–3 G13+ 0.55426∗ RBC C22:6*n*–3 G13) + (0.264646 ∗ RBC LCPUFA*n*–3 G13) – (0.028367 ∗ RBC *n*–6/*n*–3 ratio G13) + 12.6716]. The Venn diagram represents FA, which are in common between the different gestation days (N). Black circle, CTL; gray circle, *n*–3 DEF; Opened circle, EPA+DHA. Data are mean ± SEM and individual values (*n =* 5–10). ∗*P <* 0.05, ∗∗*P <* 0.01, ∗∗∗*P <* 0.001, 1-way ANOVA with Tukey posttest. AA, arachidonic acid; ALA, alpha-linolenic acid; ANOVA, analysis of variance; CTL, control diet; DPA, docosapentaenoic acid; EPA+DHA, EPA-, DHA-, and AA-enriched diet; FA, fatty acid; LA, linoleic acid; LCPUFA, long-chain PUFA; *n*–3 DEF, *n*–3 deficient diet; PLS, partial least squares; PND, postnatal day.

Although total brain LCPUFA content remained consistent across groups, *n*–3 LCPUFA levels were significantly reduced in the *n*–3 DEF group (*P <* 0.001; Figure 3A; Table 4). There was a concurrent increase in *n*–6 LCPUFAs (*P <* 0.001; Figure 3B), which resulted in a higher *n*–6/*n*–3 ratio (Figure 3C). *n*–3 DPA and DHA levels decreased significantly in the *n*–3 DEF group (Table 4; Figure 3D). Interestingly, despite the higher dietary provision, LA levels in the pup hypothalamus were unchanged in the *n*–3 DEF group (Figure 3E). AA, provided through the EPA+DHA diet, did not significantly rise in the EPA+DHA group (Figure 3F). However, the *n*–3 DEF group showed a slight increase in AA and a substantial 3.6-fold elevation in *n*–6 DPA (Figure 3F, G). The oleic acid-to-LA ratio, a marker of brain myelination [23], trended higher in the EPA+DHA group at birth (Figure 3H), becoming significant by postnatal day (PND)21 (Supplemental Figure 2).

**TABLE 4:** Percentage of selected fatty acids in the hypothalamus of offspring at birth

	CTL	*n*–3 DEF	EPA+DHA	ANOVA
14:0	1.56 ± 0.05	1.64 ± 0.04	1.70 ± 0.04	ns
15:0	n.d.	n.d.	n.d.	—
16:0	32.0 ± 0.2	32.42 ± 0.31	31.86 ± 0.32	ns
18:0	17.6 ± 0.6	17.68 ± 0.39	17.62 ± 0.39	ns
20:0	n.d.	n.d.	n.d.	—
22:0	n.d.	n.d.	n.d.	—
24:0	n.d.	n.d.	n.d.	—
**SCFA**	1.56 ± 0.05	1.64 ± 0.04	1.73 ± 0.04	ns
**MCFA**	49.6 ± 0.8	50.10 ± 0.67	49.67 ± 0.70	ns
**LCFA**	n.d.	n.d.	n.d.	—
**Total SFA**	51.2 ± 0.8	51.76 ± 0.65	51.25 ± 0.72	Ns
16:1 *n*–9	2.33 ± 0.04	2.25 ± 0.03	2.31 ± 0.03	Ns
16:1 *n*–7	1.50 ± 0.06	1.51 ± 0.04	1.56 ± 0.03	Ns
18:1 *n*–9	12.7 ± 0.4	11.30 ± 0.11∗∗	12.04 ± 0.25#	<0.01
18:1 *n*–7	2.99 ± 0.05	2.98 ± 0.05	3.10 ± 0.06	Ns
20:1	0.35 ± 0.01	0.29 ± 0.02	0.40 ± 0.14###	<0.01
22:1	n.d.	n.d.	n.d.	—
24:1	n.d.	n.d.	n.d.	—
**Total MUFA**	19.9 ± 0.5	18.34 ± 0.17∗	19.49 ± 0.38#	<0.05
18:2 *n*–6 (LA)	0.58 ± 0.06	0.60 ± 0.04	0.55 ± 0.08	ns
20:2 *n*–6	n.d.	n.d.	n.d.	—
20:3 *n*–6	0.32 ± 0.04	0.28 ± 0.03	0.26 ± 0.02	Ns
20:4 *n*–6 (AA)	11.1 ± 0.1	11.98 ± 0.20	10.77 ± 0.28##	<0.01
22:4 *n*–6	3.35 ± 0.09	3.38 ± 0.07	3.08 ± 0.13	Ns
22:5 *n*–6 (DPA)	1.98 ± 0.08	7.15 ± 0.26∗∗∗	2.05 ± 0.06###	<0.001
**n-6 LCPUFA**	17.6 ± 0.2	23.57 ± 0.34∗∗∗	16.99 ± 0.37###	<0.001
18:3 *n*–3 (ALA)	n.d.	n.d.	n.d.	—
20:5 *n*–3 (EPA)	n.d.	n.d.	n.d.	—
22:5 *n*–3 (DPA)	0.53 ± 0.08	0.23 ± 0.02∗	0.41 ± 0.05#	<0.05
22:6 *n*–3 (DHA)	11.0 ± 0.3	6.19 ± 0.41∗∗∗	11.65 ± 0.31###	<0.001
***n*–3 LCPUFA**	11.3 ± 0.3	6.33 ± 0.40∗∗∗	12.27 ± 0.27###	<0.001
**Total LCPUFA**	28.9 ± 0.4	29.90 ± 0.63	29.26 ± 0.43	ns
***n*–6/*n*–3 ratio**	1.56 ± 0.05	3.87 ± 0.27∗∗∗	1.39 ± 0.05###	<0.001

Values are mean ± SEM. *N =* 4–10.

One-way ANOVA with the Tukey post hoc test.

Abbreviations: AA, arachidonic acid; ALA, alpha-linolenic acid; ANOVA, analysis of variance; CTL, control diet; DPA, docosapentaenoic acid; LA, linoleic acid; LCFA, long-chain SFA; LCPUFA, long-chain PUFA; MCFA, medium-chain SFA; *n*–3 DEF, *n*–3 deficient diet; n.d., not detected; ns, not significant; SCFA, short-chain SFA.

∗ *P <* 0.05

∗∗ *P <* 0.01

∗∗∗ *P <* 0.001 compared with CTL.

# *P <* 0.05

## *P <* 0.01

### *P <* 0.001 compared with *n*–3 DEF.

### Maternal RBC FA composition as a predictive value for offspring brain DHA

DHA, *n*–3 LCPUFA, *n*–6 DPA, and the *n*–6/*n*–3 ratio in mother RBC membranes were positively correlated with their respective levels in the offspring hypothalamus at birth (Figure 3I). Using PLS regression, we successfully predicted offspring brain DHA concentrations in offspring based on maternal RBC membrane profiles at multiple time points (Figure 3J, K). Notably, a strong coefficient of determination for DHA was observed at G6 (*R*^2^ = 0.8541; Figure 3L) and G13 (*R*^2^ = 0.9470; Figure 3M). These results indicate key predictors including DHA, *n*–3 LCPUFA, and the *n*–6/*n*–3 ratio (Figure 3N). Although similar trends were noted at G19, L6, L13, and L19, these data were excluded from further discussion because of the reduced model reliability, likely resulting from a limited sample size (Supplemental Figure 3).

### The FA composition of the maternal diet influences both the FA composition of the milk and the developing brain at weaning

Notably, diets deficient in *n*–3 and diets enriched in EPA, DHA, and AA led to distinct changes in milk composition and subsequent effects on the offspring’s brain.

**FIGURE 4:**
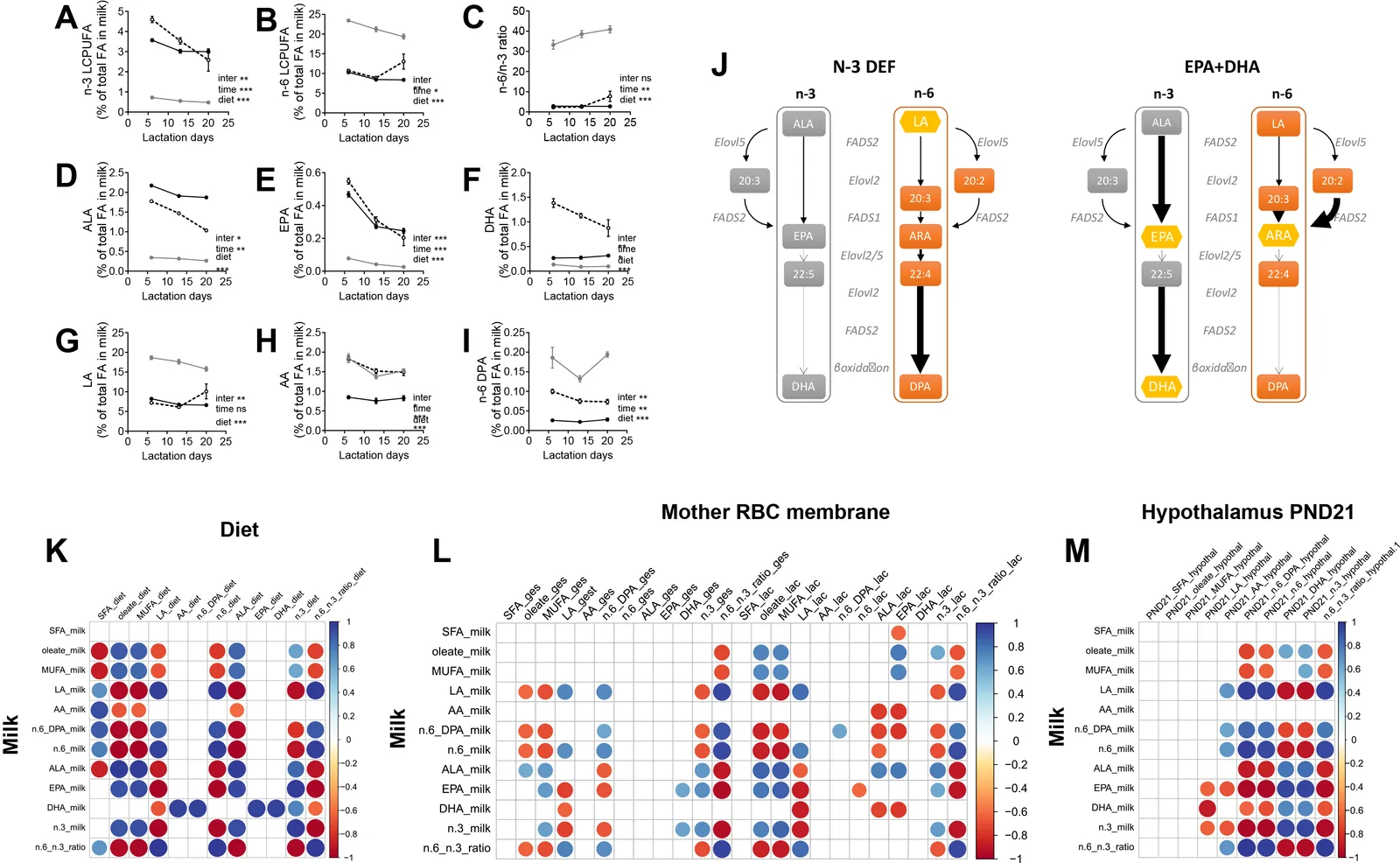
Evolution of selected fatty acid (FA) percentage in maternal milk. Evolution of *n*–3 LCPUFA (A), *n*–6 LCPUFA (B), *n*–6/*n*–3 ratio (C), ALA (D), EPA (E), DHA (F), LA (G), AA (H), and *n*–6 DPA (I) in maternal milk during the lactation period. Estimated rate of *n*–3 and *n*–6 synthesis in mammary gland based on calculated ratios of different LCPUFA synthesis steps with milk FA profiles (J). Significant correlations >0.6 between selected fatty acids in milk compared with diet (K), mother red blood cell (RBC) membrane during gestation and lactation (L), and male and female hypothalamus at PND21 (J) are presented in a correlation matrix. Solid line, CTL; gray line, *n*–3 DEF; Dotted black line, EPA+DHA. Data are mean ± SEM (*n =* 5-6). *P <* 0.05, ∗∗*P <* 0.01, ∗∗∗*P <* 0.001, 2-way ANOVA with Tukey posttest. AA, arachidonic acid; ALA, alpha-linolenic acid; ANOVA, analysis of variance; CTL, control diet; DPA, docosapentaenoic acid; EPA+DHA, EPA-, DHA-, and AA-enriched diet; LA, linoleic acid; LCPUFA, long-chain PUFA; *n*–3 DEF, *n*–3 deficient diet; PND, postnatal day.

Regarding the effects on milk FA composition, total LCPUFAs in milk significantly increased in the *n*–3 DEF group (*P <* 0.001; Table 5). However, *n*–3 LCPUFAs were markedly reduced in the *n*–3 DEF (*P <* 0.001; Figure 4A), whereas *n*–6 LCPUFAs exhibited a 2-fold increase (*P <* 0.001; Figure 4B). Consequently, the *n*–6/*n*–3 ratio was 35 in the *n*–3 DEF group and 3 in the EPA+DHA–enriched group and close to the CTL group (*P <* 0.001; Figure 4C).

**TABLE 5:** Percentage of fatty acid in maternal milk during the lactation period

	CTL			*n*–3 DEF			EPA+DHA			2-way ANOVA		
	L6	L13	L19	L6	L13	L19	L6	L13	L19	*Inter*	*Time*	*Maternal diet*
8:0	3.1 ± 0.3	4.2 ± 0.2	5.8 ± 0.4	3.7 ± 0.3	4.1 ± 0.1	6.0 ± 0.3	3.6 ± 0.2	5.1 ± 0.2∗^,^#	6.5 ± 0.3	ns	<0.001	<0.05
10:0	8.7 ± 0.4	12.6 ± 0.4	15.5 ± 0.6	9.6 ± 0.6	11.7 ± 0.7	15.8 ± 0.7	10.4 ± 0.3	15.1 ± 0.7∗∗^,^###	16.6 ± 0.5	ns	<0.001	<0.01
12:0	6.5 ± 0.5	10.4 ± 0.6	10.6 ± 0.6	6.9 ± 0.7	9.4 ± 0.8	10.8 ± 0.4	8.8 ± 0.2∗^,^#	12.2 ± 0.6##	11.2 ± 0.3	ns	<0.001	<0.05
14:0	6.1 ± 0.6	10.3 ± 1.0	8.4 ± 0.6	6.1 ± 0.8	9.0 ± 1.1	8.6 ± 0.4	8.1 ± 0.5	10.8 ± 0.6	8.4 ± 0.3	ns	<0.001	ns
16:0	19.9 ± 0.5	19.3 ± 0.5	15.7 ± 0.4	20.4 ± 0.2	20.6 ± 0.5	17.2 ± 0.6	19.5 ± 0.7	17.6 ± 0.3∗^,^###	15.8 ± 0.2	ns	<0.001	<0.001
18:0	2.3 ± 0.1	2.1 ± 0.1	2.1 ± 0.1	2.4 ± 0.1	2.4 ± 0.1	2.3 ± 0.1	2.3 ± 0.1	2.2 ± 0.1	2.2 ± 0.1	ns	<0.01	ns
**SCFA**	24.5 ± 1.2	37.5 ± 1.8	40.3 ± 1.8	26.3 ± 1.9	34.1 ± 2.5	41.1 ± 1.3	30.9 ± 0.6∗	43.2 ± 2.0∗^,^###	42.8 ± 0.9	ns	<0.001	<0.05
**MCFA**	22.3 ± 0.5	21.5 ± 0.5	17.8 ± 0.5	22.9 ± 0.3	23.0 ± 0.5	19.5 ± 0.7	22.0 ± 0.7	19.8 ± 0.3∗^,^###	18.1 ± 0.3	ns	<0.001	<0.001
**LCFA**	0.2 ± 0.0	0.2 ± 0.0	0.2 ± 0.0	0.1 ± 0.0	0.1 ± 0.0	0.2 ± 0.0	0.2 ± 0.0##	0.1 ± 0.0	0.1 ± 0.0	ns	<0.05	ns
**SFA**	46.9 ± 1.1	59.1 ± 2.1	58.3 ± 1.7	49.3 ± 1.7	57.2 ± 2.5	60.8 ± 1.2	53.0 ± 1.0∗	63.2 ± 1.8#	61.0 ± 0.8	ns	<0.001	<0.01
16:1 *n*–7	3.2 ± 0.3	1.4 ± 0.2	1.5 ± 0.2	3.0 ± 0.4	1.7 ± 0.3	1.3 ± 0.2	2.5 ± 0.1	1.5 ± 0.1	1.5 ± 0.1	<0.05	<0.001	ns
18:1 *n*–9	32.6 ± 0.8	25.5 ± 1.3	26.1 ± 1.1	21.2 ± 1.1∗∗∗	17.6 ± 1.6∗∗∗	16.5 ± 0.7∗∗∗	26.3 ± 0.6∗∗∗^,^##	20.7 ± 1.2∗∗	19.8 ± 1.2∗∗∗	ns	<0.001	<0.001
20:1	0.7 ± 0.0	0.6 ± 0.0	0.6 ± 0.0	0.4 ± 0.0∗∗∗	0.3 ± 0.0∗∗	0.3 ± 0.0∗∗	0.6 ± 0.1	0.5 ± 0.0#	0.4 ± 0.0	ns	<0.05	<0.001
**MUFA**	39.2 ± 0.9	29.4 ± 1.7	30.4 ± 1.4	26.5 ± 1.6∗∗∗	21.0 ± 2.1∗∗∗	19.4 ± 1.0∗∗∗	31.6 ± 0.7∗∗^,^#	24.4 ± 1.3∗	23.4 ± 1.4∗∗	ns	<0.001	<0.001
18:2 *n*–6 (LA)	8.2 ± 0.3	6.8 ± 0.3	6.6 ± 0.3	18.7 ± 0.5∗∗∗	17.6 ± 0.6∗∗∗	15.8 ± 0.6∗∗∗	7.2 ± 0.2#	6.1 ± 0.3###	10.1 ± 1.9∗^,^###	<0.01	ns	<0.001
20:2 n-6	0.3 ± 0.0	0.3 ± 0.0	0.2 ± 0.0	0.8 ± 0.0∗∗∗	0.6 ± 0.0∗∗∗	0.5 ± 0.0∗∗∗	0.4 ± 0.0###	0.3 ± 0.0###	0.3 ± 0.0##	<0.01	<0.001	<0.001
20:3 *n*–6	0.4 ± 0.0	0.3 ± 0.0	0.2 ± 0.0	0.8 ± 0.0∗∗∗	0.6 ± 0.0∗∗∗	0.4 ± 0.0∗∗∗	0.5 ± 0.0###	0.3 ± 0.0###	0.3 ± 0.0∗	<0.001	<0.001	<0.001
20:4 *n*–6 (AA)	0.9 ± 0.0	0.8 ± 0.1	0.8 ± 0.1	1.9 ± 0.1∗∗∗	1.4 ± 0.1∗∗∗	1.5 ± 0.1∗∗∗	1.8 ± 0.1∗∗∗	1.5 ± 0.1∗∗∗	1.5 ± 0.1∗∗∗	<0.05	<0.001	<0.001
22:4 *n*–6	0.2 ± 0.0	0.2 ± 0.0	0.3 ± 0.1	0.6 ± 0.1∗∗∗	0.5 ± 0.0∗∗∗	0.6 ± 0.0∗∗∗	0.5 ± 0.0∗∗∗	0.4 ± 0.0∗∗	0.5 ± 0.0∗∗^,^#	ns	<0.01	<0.001
22:5 *n*–6 (DPA)	0.0 ± 0.0	0.0 ± 0.0	0.0 ± 0.0	0.2 ± 0.0∗∗∗	0.1 ± 0.0∗∗∗	0.2 ± 0.0∗∗∗	0.1 ± 0.0∗∗∗^,^###	0.1 ± 0.0∗∗^,^###	0.1 ± 0.0∗∗^,^###	<0.01	<0.01	<0.001
**n-6 LCPUFA**	10.3 ± 0.3	8.5 ± 0.4	8.4 ± 0.3	23.5 ± 0.3∗∗∗	21.2 ± 0.6∗∗∗	19.4 ± 0.6∗∗∗	10.7 ± 0.3###	8.9 ± 0.4###	13.0 ± 1.9∗∗∗^,^###	<0.01	**<**0.05	<0.001
18:3 *n*–3 (ALA)	2.2 ± 0.1	1.9 ± 0.1	1.9 ± 0.1	0.3 ± 0.0∗∗∗	0.3 ± 0.0∗∗∗	0.3 ± 0.0∗∗∗	1.8 ± 0.0∗^,^###	1.5 ± 0.1∗^,^###	1.0 ± 0.3∗∗∗^,^###	<0.05	<0.01	<0.001
20:5 *n*–3 (EPA)	0.5 ± 0.0	0.3 ± 0.0	0.2 ± 0.0	0.1 ± 0.0∗∗∗	0.0 ± 0.0∗∗∗	0.0 ± 0.0∗∗∗	0.6 ± 0.0∗^,^###	0.3 ± 0.0###	0.2 ± 0.1###	<0.001	<0.001	<0.001
22:5 *n*–3 (DPA)	0.4 ± 0.0	0.3 ± 0.0	0.4 ± 0.0	0.1 ± 0.0∗∗∗	0.1 ± 0.0∗∗∗	0.1 ± 0.0∗∗∗	0.6 ± 0.0∗∗^,^###	0.4 ± 0.0###	0.4 ± 0.1###	**<0.05**	<0.05	<0.001
22:6 *n*–3 (DHA)	0.3 ± 0.0	0.3 ± 0.0	0.3 ± 0.0	0.1 ± 0.0	0.1 ± 0.0	0.1 ± 0.0	1.4 ± 0.1∗∗∗^,^###	1.1 ± 0.1∗∗∗^,^###	0.9 ± 0.2∗∗∗^,^###	<0.01	<0.05	<0.001
***n*–3 LCPUFA**	3.5 ± 0.1	3.0 ± 0.1	3.0 ± 0.1	0.7 ± 0.1∗∗∗	0.6 ± 0.0∗∗∗	0.5 ± 0.0∗∗∗	4.6 ± 0.2∗∗^,^###	3.5 ± 0.2###	2.6 ± 0.6###	<0.01	<0.001	<0.001
**Total LCPUFA**	13.9 ± 0.4	11.5 ± 0.5	11.4 ± 0.4	24.2 ± 0.3∗∗∗	21.8 ± 0.7∗∗∗	19.8 ± 0.6∗∗∗	15.3 ± 0.4###	12.5 ± 0.5###	15.6 ± 1.4∗∗∗^,^###	<0.01	<0.001	<0.001
**n-6/n-3 ratio**	2.9 ± 0.0	2.8 ± 0.0	2.8 ± 0.1	33.3 ± 2.2∗∗∗	38.6 ± 1.8∗∗∗	40.9 ± 1.8∗∗∗	2.3 ± 0.0###	2.5 ± 0.1###	7.7 ± 2.6∗**^,^**###	ns	<0.01	<0.001

Values are mean ± SEM. *N* = 5–6.

One-way ANOVA with the Tukey post hoc test.

Abbreviations: AA, arachidonic acid; ALA, alpha-linolenic acid; ANOVA, analysis of variance; CTL, control diet; DPA, docosapentaenoic acid; LA, linoleic acid; LCFA, long-chain SFA; LCPUFA, long-chain PUFA; MCFA, medium-chain SFA; *n*–3 DEF, *n*–3 deficient diet; ns, not significant; SCFA, short-chain SFA. ^∗^*P <* 0.05, ∗∗*P <* 0.01, ∗∗∗*P <* 0.001 compared with CTL.

# *P <* 0.05

## *P <* 0.01

### *P <* 0.001 compared with *n*–3 DEF.

Considering the shifts in individual FA, the *n*–3 DEF group exhibited a 7-fold decrease in ALA, a 3-fold decrease in EPA, and a 3-fold decrease in DHA (*P <* 0.001; Figure 4D–F) while LA, AA, and *n*–6 DPA increased (*P <* 0.001; Figure 4G–I), suggesting an enhanced *n*–6 synthesis in the mammary gland. In the EPA+DHA group, ALA decreased compared with the CTL group and continued to decline during lactation (*P <* 0.001; Figure 4D), whereas EPA slightly increased during the first week of lactation (Figure 4E). DHA increased 4-fold (*P <* 0.001; Figure 4F) due to dietary supply. The LA level remained comparable with that of the CTL group (Figure 4G). Conversely, AA levels resembled those in the *n*–3 DEF group (Figure 4H), suggesting increased downstream synthesis (Figure 4I; Table 5).

Using milk FA profiles to estimate mammary gland FA synthesis, we calculated FA ratios and estimated the activation of different LCPUFA synthesis steps. In the *n*–3 DEF group, *n*–3 synthesis appeared impaired at the EPA production step, favoring the *n*–6 pathway and DPA production (Table 6 and Figure 4J). In the EPA+DHA group, *n*–3 FA derivatives increased at every step. Early *n*–6 synthesis steps were also upregulated, likely due to the direct impact of EPA, DHA, and AA enrichment of the diet.

Regarding the correlation between diet composition, milk, and maternal RBC FA profiles, we observed strong positive correlations between dietary and milk levels of MUFA (including oleic acid), *n*–6 LCPUFA (including LA), *n*–3 LCPUFA (including ALA and DHA), and the *n*–6/*n*–3 ratio (Figure 4K), suggesting a substantial influence of maternal diets on milk FA composition. The precursor LA in milk was negatively correlated with *n*–3 LCPUFAs and positively correlated with the *n*–6/*n*–3 ratio in diet, whereas ALA was positively correlated with *n*–3 LCPUFAs and negatively correlated with the *n*–6/*n*–3 ratio in diet. Importantly, LA and ALA in the diet were not directly correlated with downstream AA and DHA levels in the milk.

LA, *n*–6 DPA, *n*–3 LCPUFAs, and the *n*–6/*n*–3 ratio in milk were positively correlated with their levels in mother RBC membrane during gestation and lactation (Figure 4L). LA in milk was negatively correlated with *n*–3 LCPUFAs and positively correlated with *n*–6/*n*–3 ratio in mother RBC membranes, whereas ALA in milk was positively correlated with *n*–3 LCPUFAs and negatively correlated with *n*–6/*n*–3 ratio in mother RBC membranes.

Regarding the impact on offspring brain FA composition, positive correlations were found between specific milk FAs and hypothalamus FA at PND21 (Figure 4J), namely *n*–6 DPA, *n*–6 LCPUFA, DHA, *n*–3 LCPUFA, and *n*–6/*n*–3 ratio. LA in milk was positively correlated with *n*–6 LCPUFAs and *n*–6/*n*–3 ratio in offspring hypothalamus at PND21 and negatively correlated with *n*–3 LCPUFAs. ALA in milk was positively correlated with hypothalamic DHA, highlighting the role of milk in brain DHA accretion. The prefrontal cortex showed similar trends (Supplemental Table 3). By PND21, FA profiles in the hypothalamus were stable and similar to those at birth, with no observed sex differences (Tables 4 and 5, Tables 6 and 7), and the marked effect of the maternal diet was still evident (Supplemental Figure 2).

**TABLE 6:** Fatty acid ratio in maternal milk

	CTL	*n*-3 DEF	EPA+DHA	ANOVA
20:2 *n*–6/18:2 *n*–6 (LA)	0.038 ± 0.001	0.035 ± 0.003	0.041 ± 0.001	ns
20:3 *n*–6/18:2 *n*–6 (LA)	0.043 ± 0.002	0.035 ± 0.002	0.050 ± 0.023###	<0.01
20:4 *n*–6 (AA)/18:2 *n*–6 (LA)	0.113 ± 0.006	0.092 ± 0.005	0.215 ± 0.023∗∗∗^,^###	<0.001
20:4 *n*–6 (AA)/ 20:2 *n*–6	2.98 ± 0.13	2.60 ± 0.11	5.27 ± 0.45∗∗∗^,^###	<0.001
20:4 *n*–6 (AA)/ 20:3 *n*–6	2.62 ± 0.12	2.62 ± 0.09	4.24 ± 0.29∗∗∗^,^###	<0.001
22:4 *n*–6/20:4 *n*–6 (AA)	0.28 ± 0.02	0.36 ± 0.01∗	0.28 ± 0.01#	<0.01
22:5 *n*–6 (DPA)/ 20:4 *n*–6 (AA)	0.032 ± 0.001	0.108 ± 0.005∗∗∗	0.051 ± 0.001∗∗^,^###	<0.001
22:5 *n*–6 (DPA)/ 22:4 *n*–6	0.11 ± 0.01	0.30 ± 0.01∗∗∗	0.18 ± 0.01∗∗∗^,^###	<0.001
22:5 *n*–6 (DPA)/ 18:2 *n*–6 (LA)	0.004 ± 0.000	0.010 ± 0.001∗∗∗	0.011 ± 0.001∗∗∗	<0.001
20:5 *n*–3 (EPA)/18:3 *n*–3 (ALA)	0.166 ± 0.007	0.157 ± 0.011	0.251 ± 0.009∗∗∗^,^###	<0.001
22:5 *n*–3 (DPA)/20:5 *n*–3 (EPA)	1.10 ± 0.06	1.58 ± 0.06∗∗∗	1.25 ± 0.06##	<0.001
22:6 *n*–3 (DHA)/ 22:5 *n*–3 (DPA)	0.80 ± 0.04	1.40 ± 0.06∗∗∗	2.56 ± 0.010∗∗∗^,^###	<0.001
22:6 *n*–3 (DHA)/ 20:5 *n*–3 (EPA)	0.87 ± 0.03	2.21 ± 0.09∗∗∗	3.17 ± 0.07∗∗∗^,^###	<0.001
22:6 *n*–3 (DHA)/ 18:3 *n*–3 (ALA)	0.144 ± 0.008	0.349 ± 0.036∗∗∗	0.794 ± 0.029∗∗∗^,^###	<0.001
18:2 *n*–6 (LA)/ 18:3 *n*–3 (ALA)	3.62 ± 0.05	56.18 ± 1.43∗∗∗	5.74 ± 0.83###	<0.001
20:4 *n*–6 (AA)/22:6 *n*–3 (DHA)	2.84 ± 0.11	15.15 ± 1.18∗∗∗	1.44 ± 0.04###	<0.001

Values are mean ± SEM. *N =* 5–6.

One-way ANOVA with the Tukey post hoc test.

Abbreviations: AA, arachidonic acid; ALA, alpha-linolenic acid; ANOVA, analysis of variance; CTL, control diet; DPA, docosapentaenoic acid; LA, linoleic acid; *n*–3 DEF, *n*–3 deficient diet.

∗ *P <* 0.05

∗∗ *P <* 0.01

∗∗∗ *P <* 0.001 compared with CTL.

# *P <* 0.05

## *P <* 0.01

### *P <* 0.001 compared with n-3 DEF.

**TABLE 7:** Percentage of selected fatty acid in the hypothalamus of male and female offspring at PND21–22

	CTL	*n*–3 DEF	EPA+DHA	CTL	*n*–3 DEF	EPA+DHA	2-way ANOVA		
	*Male*			*Female*			*Inter*	*Sex*	*Maternal diet*
14:0	0.33 ± 0.01	0.34 ± 0.2	0.37 ± 0.02	0.34 ± 0.01	0.31 ± 0.01	0.34 ± 0.01	ns	ns	ns
15:0	0.08 ± 0.01	0.07 ± 0.01	0.07 ± 0.00	0.09 ± 0.01	0.09 ± 0.01	0.08 ± 0.01	ns	ns	ns
16:0	25.3 ± 0.3	24.9 ± 0.2	25.0 ± 0.2	25.0 ± 0.4	25.0 ± 0.2	24.9 ± 0.3	ns	ns	ns
18:0	20.6 ± 0.1	20.8 ± 0.1	21.1 ± 0.2	20.8 ± 0.2	20.7 ± 0.2	21.0 ± 0.2	ns	ns	ns
20:0	0.23 ± 0.02	0.23 ± 0.01	0.22 ± 0.02	0.26 ± 0.03	0.24 ± 0.02	0.25 ± 0.01	ns	ns	ns
22:0	0.21 ± 0.02	0.21 ± 0.01	0.19 ± 0.03	0.23 ± 0.04	0.21 ± 0.02	0.23 ± 0.01	ns	ns	ns
24:0	0.24 ± 0.04	0.24 ± 0.02	0.31 ± 0.06	0.23 ± 0.06	0.24 ± 0.03	0.27 ± 0.04	ns	ns	ns
**SCFA**	0.33 ± 0.01	0.35 ± 0.02	0.37 ± 0.02	0.34 ± 0.01	0.33 ± 0.01	0.34 ± 0.01	ns	ns	ns
**MCFA**	46.9 ± 0.2	45.7 ± 0.2	46.2 ± 0.3	45.9 ± 0.4	45.8 ± 0.2	46.0 ± 0.2	ns	ns	ns
**LCFA**	0.67 ± 0.08	0.69 ± 0.04	0.72 ± 0.12	0.72 ± 0.13	0.69 ± 0.07	0.75 ± 0.05	ns	ns	ns
**Total SFA**	47.0 ± 0.2	46.7 ± 0.2	47.3 ± 0.2	47.0 ± 0.3	46.8 ± 0.1	47.1 ± 0.2	ns	ns	ns
16:1 *n*–9	0.43 ± 0.02	0.39 ± 0.01	0.45 ± 0.01	0.47 ± 0.02	0.39 ± 0.01∗	0.42 ± 0.02	*P =* 0.09	ns	<0.01
16:1 *n*–7	0.43 ± 0.01	0.43 ± 0.02	0.44 ± 0.01	0.44 ± 0.01	0.41 ± 0.01	0.44 ± 0.00	ns	ns	ns
18:1 *n*–9	14.1 ± 0.2	13.3 ± 0.2	13.9 ± 0.3	14.3 ± 0.4	13.4 ± 0.2	14.1 ± 0.1	ns	ns	ns
20:1	0.46 ± 0.02	0.44 ± 0.03	0.43 ± 0.03	0.45 ± 0.04	0.44 ± 0.02	0.46 ± 0.01	ns	ns	ns
22:1	0.06 ± 0.01	0.07 ± 0.01	0.05 ± 0.01	0.07 ± 0.01	0.09 ± 0.01	0.07 ± 0.01	ns	ns	ns
24:1	0.25 ± 0.04	0.27 ± 0.03	0.22 ± 0.04	0.29 ± 0.06	0.25 ± 0.03	0.25 ± 0.01	ns	ns	ns
**MUFA**	18.7 ± 0.3	17.9 ± 0.3	18.2 ± 0.5	18.9 ± 0.6	18.0 ± 0.3	18.6 ± 0.1	ns	ns	ns
18:2 *n*–6 (LA)	0.62 ± 0.03	0.72 ± 0.01	0.40 ± 0.01∗∗∗^,^###	0.66 ± 0.05	0.65 ± 0.01	0.43 ± 0.01∗∗∗^,^###	ns	ns	<0.001
20:2 *n*–6	0.09 ± 0.01	0.13 ± 0.01	0.05 ± 0.01##	0.12 ± 0.02	0.12 ± 0.01	0.06 ± 0.01#	ns	ns	<0.001
20:3 *n*–6	0.52 ± 0.02	0.39 ± 0.01∗∗	0.36 ± 0.01∗∗∗	0.53 ± 0.04	0.36 ± 0.02∗∗∗	0.39 ± 0.01∗∗∗	ns	ns	<0.001
20:4 *n*–6 (AA)	12.5 ± 0.2	13.2 ± 0.1∗	12.6 ± 0.1	12.7 ± 0.2	13.2 ± 0.1	12.4 ± 0.1##	ns	ns	<0.001
22:4 *n*–6	4.52 ± 0.03	4.84 ± 0.07∗∗	4.39 ± 0.05###	4.53 ± 0.02	4.89 ± 0.03∗∗∗	4.36 ± 0.08∗∗∗	ns	ns	<0.001
22:5 *n*–6 (DPA)	0.97 ± 0.01	5.06 ± 0.27∗∗∗	0.90 ± 0.02###	0.96 ± 0.03	4.91 ± 0.31∗∗∗	0.84 ± 0.03###	ns	ns	<0.001
***n*–6 LCPUFA**	19.3 ± 0.2	24.4 ± 0.2∗∗∗	18.8 ± 0.1###	19.5 ± 0.2	24.72 ± 0.2∗∗∗	18.5 ± 0.2∗^,^###	ns	ns	<0.001
18:3 *n*–3 (ALA)	—	—	—	—	—	—			
20:5 *n*–3 (EPA)	—	—	—	—	—	—			
22:5 *n*–3 (DPA)	0.42 ± 0.02	0.19 ± 0.01∗∗∗	0.33 ± 0.01∗∗**^,^**###	0.44 ± 0.02	0.18 ± 0.01∗∗∗	0.33 ± 0.01∗∗∗**^,^**###	ns	ns	<0.001
22:6 *n*–3 (DHA)	14.6 ± 0.3	10.8 ± 0.3∗∗∗	15.4 ± 0.2###	14.1 ± 0.2	10.7 ± 0.3∗∗∗	15.4 ± 0.2**∗^,^**###	ns	ns	<0.001
***n*–3 LCPUFA**	15.1 ± 0.3	11.0 ± 0.3∗∗∗	15.7 ± 0.2###	14.5 ± 0.2	10.9 ± 0.3∗∗∗	15.8 ± 0.2∗^,^###	ns	ns	<0.001
**Total LCPUFA**	34.4 ± 0.3	35.3 ± 0.2	34.5 ± 0.3	34.1 ± 0.4	35.1 ± 0.2	34.3 ± 0.2	ns	ns	<0.01
***n*–6/*n*–3 ratio**	1.29 ± 0.03	2.23 ± 0.08∗∗∗	1.20 ± 0.01###	1.35 ± 0.01	2.23 ± 0.09∗∗∗	1.17 ± 0.02###	ns	ns	<0.001

Values are mean ± SEM.

*N =* 5.

Two-way ANOVA with the Tukey post hoc test.

Abbreviations: AA, arachidonic acid; ALA, alpha-linolenic acid; ANOVA, analysis of variance; CTL, control diet; DPA, docosapentaenoic acid; LA, linoleic acid; LCFA, long-chain SFA; LCPUFA, long-chain PUFA; MCFA, medium-chain SFA; *n*–3 DEF, *n*–3 deficient diet; ns, not significant; PND, postnatal day; SCFA, short-chain SFA.

∗*P <* 0.05, ∗∗*P <* 0.01, ∗∗∗*P <* 0.001 compared with CTL.

# *P <* 0.05

## *P <* 0.01

### *P <* 0.001 compared with *n*–3 DEF.

### The influence of maternal diet on offspring brain FA profiles vanishes after weaning

**FIGURE 5:**
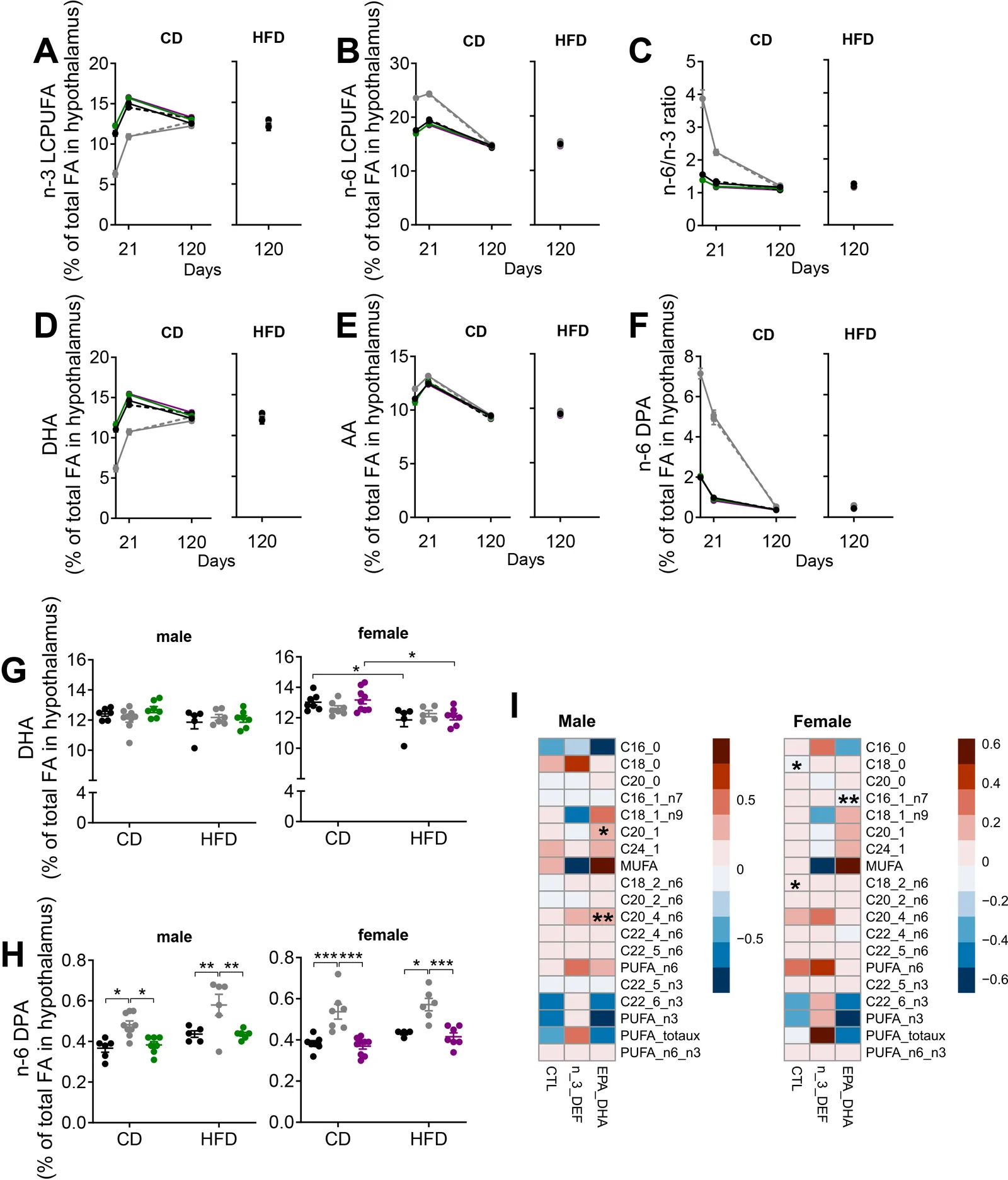
Evolution of selected fatty acid (FA) percentage in male and female hypothalamus from birth to PND120. Evolution of *n*–3 LCPUFA (A), *n*–6 LCPUFA (B), *n*–6/*n*–3 ratio (C), DHA (D), AA (E), *n*–6 DPA (F) in the hypothalamus of male and female offspring from birth to PND120 under chow (CD) and high-fat diet (HFD). Specific percentages of DHA (G) and *n*–6 DPA (H) in the hypothalamus from male and female offspring at PND120. Heatmap representing the delta of FA percentage between the unpurified diet and high-fat diet in the hypothalamus from male and female offspring at PND120 (I). Black line, CTL; gray line, *n*–3 DEF; Colored line, EPA+DHA. Solid line/green, male; dotted line/purple, female. Data are mean ± SEM and individual values (*n =* 4–10). *P <* 0.05, ∗∗*P <* 0.01, ∗∗∗*P <* 0.001, 2-way ANOVA with the Tukey post hoc test. ANOVA, analysis of variance; CTL, control diet; DPA, docosapentaenoic acid; EPA+DHA, EPA-, DHA-, and AA-enriched diet; LCPUFA, long-chain PUFA; *n*–3 DEF, *n*–3 deficient diet; PND, postnatal day.

At PND120, the initial differences in hypothalamic FA composition observed at birth and weaning had largely dissipated, especially after transitioning to a standard diet postweaning (Table 8, Figure 5A–F). Some weak effects of the maternal diet persisted, particularly in medium-chain SFA, LC-SFA, MUFA, *n*–3 LCPUFA, and the *n*–6/*n*–3 ratio. However, the only consistent change was an elevated *n*–6 DPA percentage in the *n*–3 DEF group (*P <* 0.001), albeit representing only 0.37%–0.54% of total hypothalamic FA.

**TABLE 8:** Percentage of selected fatty acids in the hypothalamus of male and female offspring at PND120

	CTL	*n*–3 DEF	EPA+DHA	CTL	*n*–3 DEF	EPA+DHA	2-way ANOVA		
	*Male*			*Female*			Inter	*Sex*	*Maternal diet*
14:0	0.11 ± 0.00	0.11 ± 0.00	0.11 ± 0.00	0.11 ± 0.00	0.11 ± 0.00	0.11 ± 0.00	ns	ns	ns
15:0	0.04 ± 0.00	0.04 ± 0.00	0.04 ± 0.00	0.04 ± 0.00	0.04 ± 0.00	0.04 ± 0.00	ns	ns	ns
16:0	20.8 ± 0.1	20.4 ± 0.1	21.0 ± 0.2	20.7 ± 0.1	20.6 ± 0.3	20.9 ± 0.1	ns	ns	*P =* 0.06
18:0	20.3 ± 0.1	20.3 ± 0.1	20.5 ± 0.1	20.9 ± 0.2&	20.6 ± 0.1	20.8 ± .1	ns	*P <* 0.001	ns
20:0	0.51 ± 0.01	0.53 ± 0.02	0.49 ± 0.02	0.46 ± 0.02	0.51 ± .02	0.45 ± 0.02	ns	*P <* 0.05	*P <* 0.05
22:0	0.46 ± 0.02	0.50 ± 0.03	0.49 ± 0.04	0.43 ± 0.02	0.47 ± 0.02	0.43 ± 0.02	ns	ns	ns
24:0	0.92 ± 0.02	0.97 ± 0.05	0.80 ± 0.07	0.83 ± 0.05	0.90 ± 0.03	0.80 ± 0.05	ns	ns	*P <* 0.05
**SCFA**	0.11 ± 0.00	0.11 ± 0.00	0.11 ± 0.00	0.11 ± 0.00	0.11 ± 0.00	0.11 ± 0.00	ns	ns	ns
**MCFA**	41.1 ± 0.1	40.8 ± 0.2	41.5 ± 0.3	41.6 ± 0.2	41.2 ± 0.3	41.7 ± 0.2	ns	ns	*P <* 0.05
**LCFA**	1.90 ± 0.05	2.00 ± 0.10	1.79 ± 0.06	1.72 ± 0.07	1.88 ± 0.07	1.67 ± 0.09	ns	*P <* 0.05	*P <* 0.05
**Total SFA**	43.1 ± 0.1	42.9 ± 0.1	43.4 ± 0.3	43.4 ± 0.1	43.2 ± 0.2	43.5 ± 0.1	ns	ns	ns
16:1 *n*–9	0.12 ± 0.01	0.11 ± 0.00	0.11 ± 0.010	0.12 ± 0.01	0.12 ± 0.01	0.12 ± 0.00	ns	ns	ns
16:1 *n*–7	0.34 ± 0.01	0.35 ± 0.00	0.39 ± 0.06	0.34 ± 0.00	0.34 ± 0.01	0.35 ± 0.01	ns	ns	ns
18:1 *n*–9	21.3 ± 0.1	21.5 ± 0.1	20.8 ± 0.2	21.1 ± 0.2	21.0 ± 0.2	21.0 ± 0.1	ns	ns	*P =* 0.06
20:1	1.87 ± 0.04	2.02 ± 0.07	1.75 ± 0.07	1.80 ± 0.04	1.95 ± 0.11	1.72 ± 0.07	ns	ns	*P <* 0.01
22:1	0.20 ± 0.01	0.23 ± 0.01	0.21 ± 0.02	0.20 ± 0.01	0.24 ± 0.02	0.20 ± 0.01	ns	ns	ns
24:1	1.49 ± 0.04	1.55 ± 0.08	1.40 ± 0.07	1.40 ± 0.07	1.51 ± 0.09	1.36 ± 0.07	ns	ns	ns
**MUFA**	29.6 ± 0.1	30.0 ± 0.3	28.8 ± 0.4	29.1 ± 0.2	29.4 ± 0.4	28.8 ± 0.3	ns	ns	*P <* 0.05
18:2 *n*–6 (LA)	0.51 ± 0.01	0.46 ± 0.03	0.45 ± 0.03	0.43 ± 0.02	0.41 ± 0.01	0.41 ± 0.01	ns	*P <* 0.01	ns
20:2 *n*–6	0.10 ± 0.00	0.09 ± 0.00	0.08 ± 0.01	0.08 ± 0.01	0.08 ± 0.00	0.07 ± 0.01	ns	*P <* 0.01	*P <* 0.05
20:3 *n*–6	0.32 ± 0.01	0.31 ± 0.01	0.31 ± 0.01	0.31 ± 0.01	0.32 ± 0.01	0.29 ± 0.01	ns	ns	ns
20:4 *n*–6 (AA)	9.5 ± 0.0	9.5 ± 0.1	9.4 ± 0.1	9.2 ± 0.1	9.2 ± 0.1	9.4 ± 0.1	ns	*P <* 0.001	ns
22:4 *n*–6	3.89 ± 0.02	3.94 ± 0.06	3.89 ± 0.05	3.88 ± 0.02	3.97 ± 0.09	3.88 ± 0.04	ns	ns	ns
22:5 *n*–6 (DPA)	0.37 ± 0.02	0.48 ± 0.02∗∗	0.38 ± 0.02#	0.38 ± 0.01	0.54 ± 0.04∗∗∗	0.37 ± 0.01###	ns	ns	*P <* 0.001
***n*–6 LCPUFA**	14.7 ± 0.02	14.9 ± 0.1	14.7 ± 0.1	14.3 ± 0.1	14.6 ± 0.2	14.4 ± 0.1	ns	*P <* 0.001	ns
18:3 *n*–3 (ALA)	—	—	—	—	—	—	—	—	—
20:5 *n*–3 (EPA)	—	—	—	—	—	—	—	—	—
22:5 *n*–3 (DPA)	0.15 ± 0.00	0.14 ± 0.01	0.15 ± 0.01	0.15 ± 0.00	0.15 ± 0.00	0.15 ± 0.00	ns	ns	ns
22:6 *n*–3 (DHA)	12.4 ± 0.2	12.1 ± 0.2	12.7 ± 0.2	13.0 ± 0.2	12.6 ± 0.2	13.2 ± 0.2	ns	*P <* 0.01	*P <* 0.05
***n*–3 LCPUFA**	12.6 ± 0.2	12.2 ± 0.2	13.0 ± 0.2	13.2 ± 0.2	12.8 ± 0.2	13.3 ± 0.2	ns	*P <* 0.01	*P <* 0.05
**Total LCPUFA**	27.3 ± 0.2	27.1 ± 0.3	27.8 ± 0.2	27.5 ± 0.1	27.4 ± 0.2	27.7 ± 0.2	ns	ns	ns
***n*–6/*n*–3 ratio**	1.17 ± 0.01	1.22 ± 0.02	1.13 ± 0.01	1.09 ± 0.02	1.14 ± 0.02	1.09 ± 0.02	ns	*P <* 0.001	*P <* 0.01

Values are mean ± SEM. *N =* 5–9. Two-way ANOVA with the Tukey post hoc test.

Abbreviations: AA, arachidonic acid; ALA, alpha-linolenic acid; ANOVA, analysis of variance; CTL, control diet; DPA, docosapentaenoic acid; LA, linoleic acid; LCFA, long-chain SFA; LCPUFA, long-chain PUFA; MCFA, medium-chain SFA; *n*–3 DEF, *n*–3 deficient diet; ns, not significant; PND, postnatal day; SCFA, short-chain SFA.

∗∗ *P <* 0.01

∗∗∗ *P <* 0.001 compared with CTL.

# *P <* 0.05

### *P <* 0.001 compared with *n*–3 DEF.

& compared with females.

Sex-specific effects were observed for stearic acid, LA, AA, and DHA, and several FA classes such as LC-SFA, *n*–6 LCPUFA, *n*–3 LCPUFA, and the *n*–6/*n*–3 ratio (Table 8).

To assess the interaction between maternal diet and postweaning high-fat diet (HFD), offspring were fed an HFD from PND60 to PND120. No significant differences in hypothalamic FA composition were detected between groups, regardless of sex or maternal diet (Table 9), with the notable exception of *n*–6 DPA, which remained elevated in *n*–3 DEF males and females (*P <* 0.001; Figure 5H). HFD by itself notably altered hypothalamic FA composition (Figure 5I).

**TABLE 9:** Percentage of selected fatty acids in the hypothalamus of male and female offspring at PND120 after HFD

	CTL	*n*–3 DEF	EPA+DHA	CTL	*n*–3 DEF	EPA+DHA	2-way ANOVA		
	*Male*			*Female*			*Inter*	*Sex*	*Maternal diet*
14:0	0.12 ± 0.01	0.11 ± 0.00	0.12 ± 0.01	0.12 ± 0.01	0.11 ± 0.00	0.11 ± 0.00	ns	ns	ns
15:0	0.04 ± 0.00	0.04 ± 0.00	0.04 ± 0.00	0.04 ± 0.00	0.04 ± 0.00	0.04 ± 0.00	ns	ns	ns
16:0	20.4 ± 0.1	20.2 ± 0.2	20.3 ± 0.2	20.7 ± 0.3	20.9 ± 0.2	20.5 ± 0.2	ns	*P <* 0.05	ns
18:0	20.6 ± 0.1	20.9 ± 0.2	20.6 ± 0.2	20.7 ± 0.1	20.6 ± 0.1	20.9 ± 0.1	ns	ns	ns
20:0	0.53 ± 0.02	0.52 ± 0.01	0.56 ± 0.01	0.48 ± 0.01	0.46 ± 0.03	0.50 ± 0.02	ns	*P <* 0.01	ns
22:0	0.52 ± 0.02	0.49 ± 0.02	0.51 ± 0.03	0.46 ± 0.02	0.44 ± 0.01	0.46 ± 0.01	ns	*P <* 0.01	ns
24:0	0.97 ± 0.03	0.92 ± 0.06	0.93 ± 0.06	0.89 ± 0.05	0.81 ± 0.03	0.87 ± 0.04	ns	*P <* 0.05	ns
**SCFA**	0.12 ± 0.01	0.11 ± 0.00	0.12 ± 0.01	0.12 ± 0.00	0.11 ± 0.00	0.11 ± 0.00	ns	ns	ns
**MCFA**	41.1 ± 0.0	41.1 ± 0.2	40.9 ± 0.3	41.5 ± 0.2	41.5 ± 0.2	41.4 ± 0.2	ns	*P <* 0.05	*P <* 0.05
**LCFA**	2.03 ± 0.06	1.92 ± 0.08	1.99 ± 0.09	1.84 ± 0.07	1.71 ± 0.07	1.83 ± 0.07	ns	*P <* 0.01	*P <* 0.05
**Total SFA**	43.2 ± 0.1	43.2 ± 0.2	43.1 ± 0.2	43.5 ± 0.1	43.3 ± 0.1	43.3 ± 0.1	ns	*P =* 0.07	ns
16:1 *n*–9	0.11 ± 0.00	0.11 ± 0.01	0.11 ± 0.00	0.13 ± 0.01	0.12 ± 0.01	0.12 ± 0.00	ns	*P <* 0.01	ns
16:1 *n*–7	0.32 ± 0.00	0.32 ± 0.01	0.31 ± 0.00	0.33 ± 0.01	0.32 ± 0.01	0.31 ± 0.00	ns	ns	ns
18:1 *n*–9	21.3 ± 0.3	20.9 ± 0.2	21.3 ± 0.2	21.1 ± 0.2	20.7 ± 0.2	21.2 ± 0.1	ns	ns	*P =* 0.06
20:1	2.04 ± 0.07	1.95 ± 0.06	2.06 ± 0.07	1.80 ± 0.11	1.81 ± 0.08	1.93 ± 0.05	ns	*P <* 0.01	ns
22:1	0.23 ± 0.01	0.23 ± 0.01	0.25 ± 0.01	0.21 ± 0.02	0.22 ± 0.01	0.23 ± 0.01	ns	ns	ns
24:1	1.70 ± 0.05	1.59 ± 0.08	1.62 ± 0.08	1.50 ± 0.11	1.40 ± 0.05	1.52 ± 0.05	ns	*P <* 0.01	ns
**MUFA**	29.9 ± 0.4	29.2 ± 0.3	29.8 ± 0.4	29.2 ± 0.4	28.7 ± 0.4	29.4 ± 0.2	ns	*P =* 0.08	ns
18:2 *n*–6 (LA)	0.52 ± 0.01	0.50 ± 0.02	0.50 ± 0.01	0.46 ± 0.02	0.47 ± 0.01	0.46 ± 0.01	ns	*P <* 0.001	ns
20:2 *n*–6	0.11 ± 0.01	0.12 ± 0.00	0.12 ± 0.00	0.09 ± 0.01	0.10 ± 0.01	0.09 ± 0.00&	ns	*P <* 0.001	ns
20:3 *n*–6	0.32 ± 0.02	0.29 ± 0.01	0.31 ± 0.00	0.30 ± 0.01	0.30 ± 0.01	0.30 ± 0.00	ns	ns	ns
20:4 *n*–6 (AA)	9.50 ± 0.11	9.76 ± 0.06	9.67 ± 0.06	9.39 ± 0.14	9.52 ± 0.07	9.33 ± 0.07	ns	*P <* 0.01	ns
22:4 *n*–6	3.94 ± 0.10	4.08 ± 0.03	3.98 ± 0.05	3.87 ± 0.03	4.03 ± 0.06	3.82 ± 0.04	ns	ns	*P <* 0.05
22:5 *n*–6 (DPA)	0.44 ± 0.02	0.58 ± 0.05∗	0.43 ± 0.01#	0.43 ± 0.01	0.57 ± 0.03∗	0.42 ± 0.02##	ns	ns	*P <* 0.001
***n*–6 LCPUFA**	14.9 ± 0.2	15.4 ± 0.1	15.0 ± 0.1	14.6 ± 0.1	15.0 ± 0.1	14.5 ± 0.1#	ns	*P <* 0.001	*P <* 0.001
18:3 *n*–3 (ALA)	—	—	—	—	—	—	—	—	—
20:5 *n*–3 (EPA)	—	—	—	—	—	—	—	—	—
22:5 *n*–3 (DPA)	0.13 ± 0.01	0.12 ± 0.01	0.13 ± 0.00	0.14 ± 0.00	0.14 ± 0.01	0.14 ± 0.00	ns	*P <* 0.01	ns
22:6 *n*–3 (DHA)	11.9 ± 0.4	12.2 ± 0.2	12.0 ± 0.2	12.6 ± 0.3	12.8 ± 0.2	12.6 ± 0.2	ns	*P <* 0.01	ns
***n*–3 LCPUFA**	12.0 ± 0.4	12.3 ± 0.2	12.1 ± 0.2	12.8 ± 0.3	12.9 ± 0.2	12.8 ± 0.2	ns	*P <* 0.01	ns
**Total LCPUFA**	26.9 ± 0.5	27.6 ± 0.3	27.2 ± 0.2	27.4 ± 0.4	27.9 ± 0.3	27.2 ± 0.2	ns	ns	ns
***n*–6/*n*–3 ratio**	1.25 ± 0.06	1.25 ± 0.02	1.24 ± 0.03	1.14 ± 0.03	1.17 ± 0.02	1.13 ± 0.03	ns	*P <* 0.001	ns

Values are mean ± SEM. *N =* 4–7. Two-way ANOVA with the Tukey post hoc test.

Abbreviations: AA, arachidonic acid; ALA, alpha-linolenic acid; ANOVA, analysis of variance; CTL, control diet; DPA, docosapentaenoic acid; HFD, high-fat diet; LA, linoleic acid; LCFA, long-chain SFA; LCPUFA, long-chain PUFA; MCFA, medium-chain SFA; *n*–3 DEF, *n*–3 deficient diet; ns, not significant; PND, postnatal day; SCFA, short-chain SFA.

∗ *P <* 0.05 compared with CTL

# *P <* 0.05

## *P <* 0.01 compared with *n*–3 DEF.

& vs. males.

### Maternal diet influences fat accumulation in response to postweaning HFD in offspring according to sex

**FIGURE 6:**
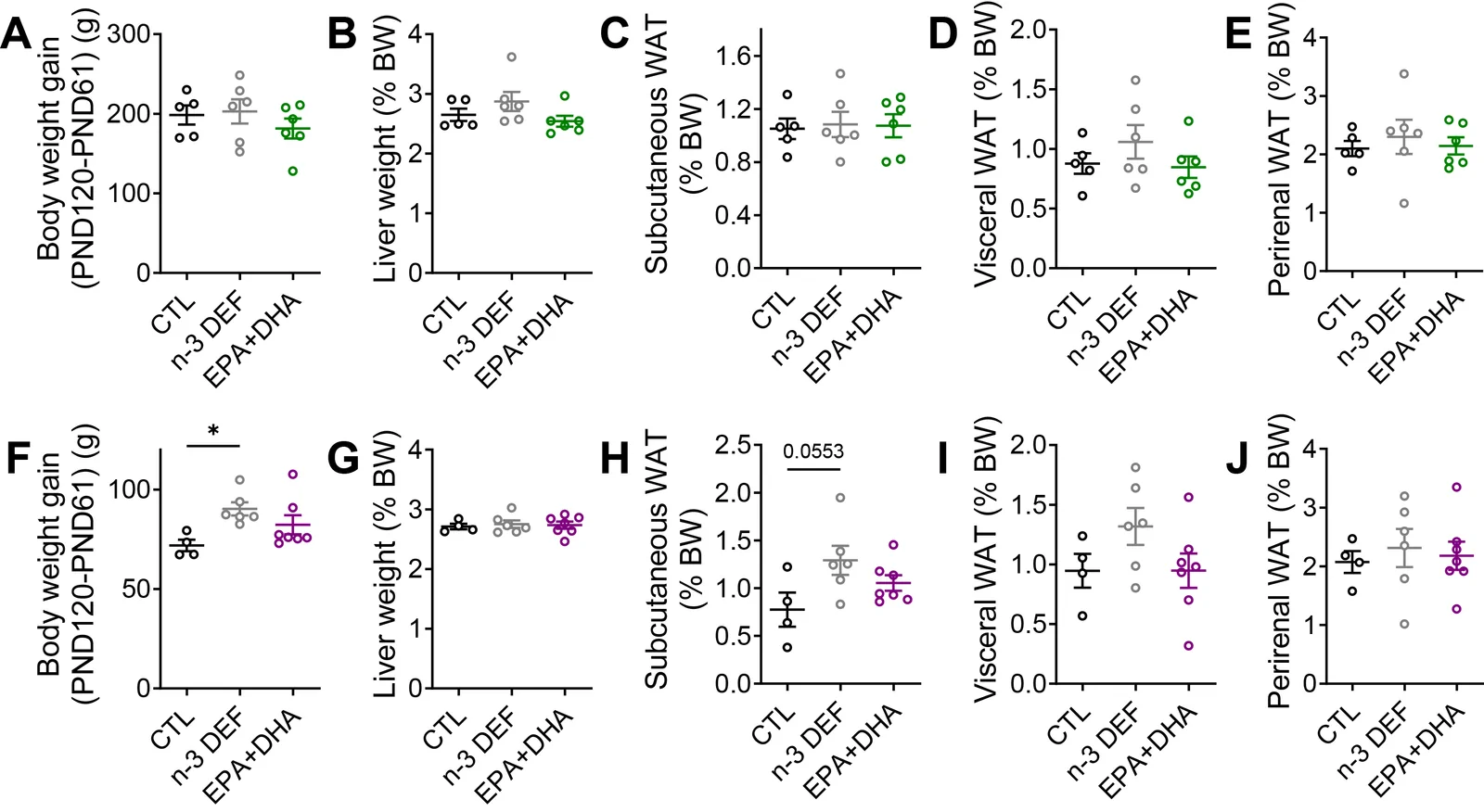
Consequences of a high-fat diet on postnatal growth of male and female offspring. Body weight gain (BW; A, F) from PND61 to PND120 under a high-fat diet in male (A) and female (F). Liver (B, G), subcutaneous (C, H), visceral (D, I), and perirenal (E, J) white adipose tissues (WAT) have been weighed at PND120 in male (A–E) and female (F–J). Black circle, CTL; gray circle, *n*–3 DEF; colored circle, EPA+DHA. Green, male; purple, female. Data are mean ± SEM or individual values (*n* = 4–7). ∗ *P <* 0.05, ∗∗ *P <* 0.01, ∗∗∗ *P <* 0.001 compared with CTL, 2-way ANOVA with Sidak posttest, unpaired *t*-test. ANOVA, analysis of variance; CTL, control diet; EPA+DHA, EPA-, DHA-, and AA-enriched diet; LCPUFA, long-chain PUFA; *n*–3 DEF, *n*–3 deficient diet; PND, postnatal day.

At the start of the challenge, all groups showed similar body weight (Supplemental Figure 4A and B). In males, no significant group differences in body weight gain (PND61–120) or fat mass accumulation were observed, regardless of maternal diet (Supplemental Figure 4C, Figure 6A–E). In females, the *n*–3 DEF group gained significantly more weight (Figure 6F) with subcutaneous fat mass that tended to increase (Figure 6G–J). EPA+DHA+AA–enriched diet appears to protect against HFD-induced fat accumulation in females.

### Offspring fasted glycemia and insulin secretion in response to glucose after a challenge with HFD at PND120 were impacted by the early maternal diet according to sex

**FIGURE 7:**
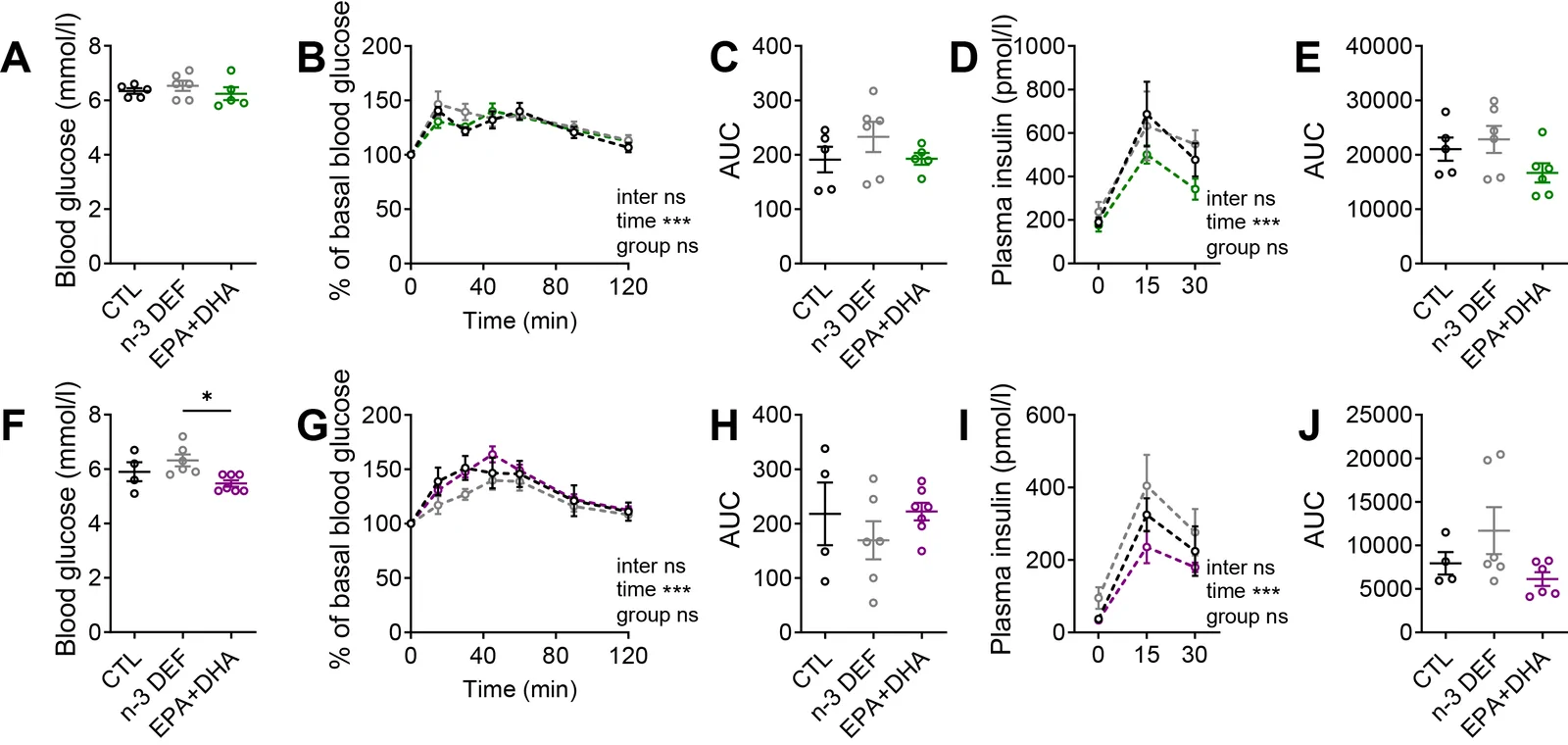
Glucose tolerance in male and female offspring under a high-fat diet on postnatal day (PND) 100. Basal blood glucose (A,F), blood glucose follow-up (B,G), and area under the curve (AUC; C,H) after oral glucose gavage (2 g/kg) in male (A–C) and female (F–H). Plasma insulin in response to glucose (D, I) and AUC (E, J) in male (D, E) and female (I, J) are presented. Solid line, CTL; gray line, *n*–3 DEF; colored line, EPA+DHA; Dotted line, high-fat diet. Black circle, CTL; gray circle, *n*–3 DEF; colored circle, EPA + DHA; opened circle, high-fat diet. Green, male; purple, female. Data are mean ± SEM or individual values (*n* = 4–7). ∗ *P <* 0.05, ∗∗ *P <* 0.01, ∗∗∗ *P <* 0.001 compared with CTL, 2-way ANOVA with Sidak posttest, unpaired *t*-test. ANOVA, analysis of variance; CTL, control diet; EPA+DHA, EPA-, DHA-, and AA-enriched diet; *n*–3 DEF, *n*–3 deficient diet.

Under chow diet (CD), females with the *n*–3 DEF group showed elevated insulin secretion, suggesting altered beta-cell function (Supplemental Figure 5). HFD challenge induced an increased insulin response to glucose gavage (Figure 7I, J) in females of the *n*–3 DEF group, whereas females of the EPA+DHA group showed a trend toward an improved glucose response, suggesting that maternal *n*–3 deficient diet had some negative influence on glucose metabolism later in life of the offspring, whereas maternal EPA+DHA+AA–enriched diet may positively influence offspring glucose handling.

### Maternal diet affects hepatic lipid metabolism in offspring according to sex

At weaning, plasma and hepatic TG levels were significantly decreased in males of *n*–3 DEF and EPA+DHA groups (*P <* 0.01), and in the liver of the females of the *n*–3 DEF group (*P <* 0.01; Supplemental Figure 7A, B). Plasma cholesterol levels were higher in females than in males (*P <* 0.05, Supplemental Figure 7C). *n*–3 DEF females had lower plasma total cholesterol levels than CTL females (*P <* 0.05). Hepatic cholesterol content was significantly decreased in males and females of the EPA+DHA group compared with both CTL and *n*–3 DEF animals (Supplemental Figure 7D). The analysis of liver FA composition at PND21–22 revealed mainly an influence of maternal diet rather than sex, in accordance with FA diet composition (Supplemental Table 4).

**FIGURE 8:**
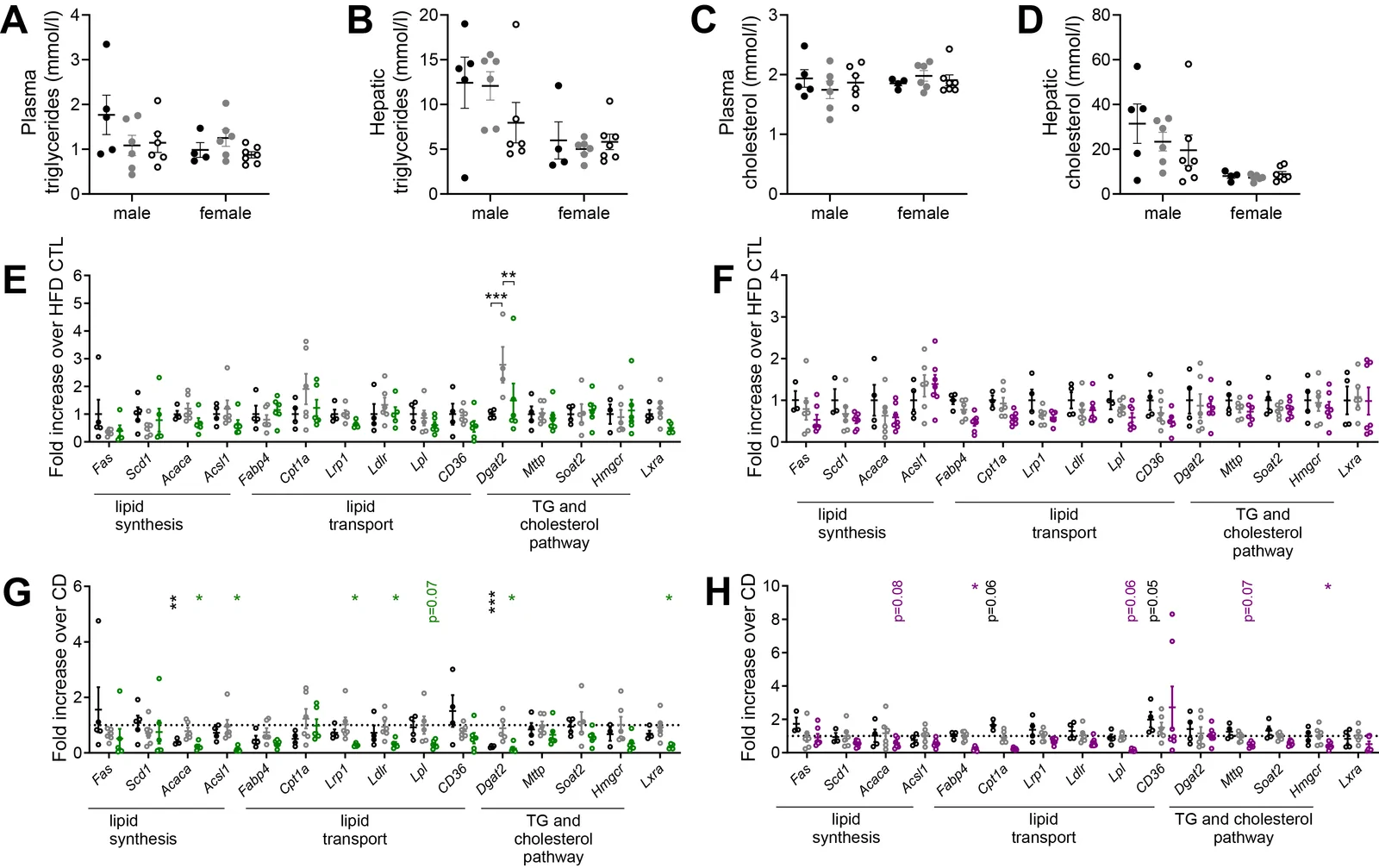
Plasma and liver levels of triglycerides and cholesterol and gene expression of lipid pathways in male and female under high-fat diet (HFD) on postnatal day (PND) 120. Triglycerides (TGs) (A, B) and cholesterol (C, D) have been measured in plasma (A, C) and liver (B, D) in male and female under a chow diet (CD) at PND120. Liver gene expression in male (E) and female (F) at PND120. Comparison between liver gene expression in unpurified diet (dotted line) compared with HFD in male (G) and female (H) at PND120. Black circle, CTL; gray circle, *n*–3 DEF; colored circle, EPA+DHA; Opened circle, HFD. Green, male; purple, female. Data are mean ± SEM or individual values (*n* = 4–7). ∗ *P <* 0.05, ∗∗ *P <* 0.01, ∗∗∗ *P <* 0.001 compared with CTL, 2-way ANOVA with Sidak posttest, unpaired *t*-test. ANOVA, analysis of variance; CTL, control diet; EPA+DHA, EPA-, DHA-, and AA-enriched diet; *n*–3 DEF, *n*–3 deficient diet.

At adulthood, post-HFD plasma TGs significantly decreased in males (*P <* 0.05; Figure 8A), whereas hepatic TG drastically increased in males and moderately in females (*P <* 0.001; Figure 8B). The analysis of liver FA composition at PDN120 revealed sex difference effects rather than maternal diet effects when we compared males and females under CD (Supplemental Table 5) or HFD (Supplemental Table 6). However, HFD exposure significantly increased MUFA levels, especially C18:1 *n*–9, in males and females independently of maternal diet. This increase was more important in male rats (Table 10) than in female rats (Table 11).

**TABLE 10:** Percentage of selected fatty acids in the liver of male offspring at PND120 after CD or HFD

	CTL	*n*–3 DEF	EPA+DHA	CTL	*n*–3 DEF	EPA+DHA	2-way ANOVA		
	*CD*			*HFD*			*Inter*	*Diet*	*Maternal diet*
14:0	0.52 ± 0.08	0.56 ± 0.07	0.45 ± 0.06	0.35 ± 0.06	0.45 ± 0.05	0.29 ± 0.05	ns	*P <* 0.01	ns
15:0	0.25 ± 0.01	0.26 ± 0.02	0.24 ± 0.02	0.11 ± 0.00^&&&^	0.13 ± 0.01^&&&^	0.11 ± 0.01^&&&^	ns	*P <* 0.001	ns
16:0	23.42 ± 0.68	23.32 ± 0.53	21.81 ± 1.00	21.78 ± 0.28	22.76 ± 1.01	20.28 ± 0.89	ns	Ns	*P <* 0.05
18:0	12.97 ± 0.68	12.56 ± 0.62	13.15 ± 1.19	10.70 ± 1.34	9.12 ± 0.71	14.39 ± 2.12#	ns	Ns	ns
20:0	0.05 ± 0.00	0.06 ± 0.00	0.07 ± 0.01∗	0.05 ± 0.00	0.05 ± 0.00	0.06 ± 0.00&	ns	*P <* 0.05	*P <* 0.01
22:0	0.11 ± 0.01	0.10 ± 0.00	0.11 ± 0.00	0.07 ± 0.01&	0.06 ± 0.01^&&^	0.09 ± 0.02	ns	*P <* 0.001	ns
24:0	0.36 ± 0.01	0.34 ± 0.01	0.38 ± 0.02	0.15 ± 0.02^&&&^	0.13 ± 0.01^&&&^	0.22 ± 0.03∗^,^##^,&&&^	ns	*P <* 0.001	*P <* 0.01
**SCFA**	0.54 ± 0.08	0.58 ± 0.07	0.48 ± 0.05	0.37 ± 0.06	0.46 ± 0.05	0.31 ± 0.05&	ns	*P <* 0.01	ns
**MCFA**	36.65 ± 0.50	36.14 ± 0.32	35.20 ± 0.61	32.60 ± 1.21^&&^	32.02 ± 0.86^&&^	34.77 ± 1.35	ns	*P <* 0.001	ns
**LCFA**	0.53 ± 0.01	0.50 ± 0.01	0.55 ± 0.03	0.27 ± 0.04^&&&^	0.23 ± 0.02^&&&^	0.37 ± 0.05#^,&&&^	ns	*P <* 0.001	*P <* 0.05
**Total SFA**	37.71 ± 0.50	37.21 ± 0.33	36.24 ± 0.64	33.23 ± 1.19^&&^	32.71 ± 0.90^&&^	35.45 ± 1.36	ns	*P <* 0.001	ns
16:1 *n*–9	0.18 ± 0.02	0.22 ± 0.02	0.19 ± 0.01	0.30 ± 0.06	0.35 ± 0.04&	0.23 ± 0.04	ns	*P <* 0.01	ns
16:1 *n*–7	2.62 ± 0.64	2.75 ± 0.33	2.33 ± 0.28	1.05 ± 0.25^&&^	1.30 ± 0.16^&&^	0.91 ± 0.23^&&^	ns	*P <* 0.001	ns
18:1 *n*–9	7.81 ± 0.73	8.57 ± 0.37	8.25 ± 0.68	28.10 ± 2.74^&&&^	33.35 ± 2.44^&&&^	23.79 ± 3.00##^,&&&^	ns	*P <* 0.001	ns
20:1	0.19 ± .01	0.20 ± 0.01	0.20 ± 0.01	0.40 ± 0.05^&&^	0.50 ± 0.07^&&&^	0.32 ± 0.04##^,^&	*P <* 0.05	*P <* 0.001	ns
22:1	0.04 ± 0.01	0.04 ± 0.00	0.05 ± 0.01	0.05 ± 0.01	0.04 ± 0.01	0.04 ± 0.01			
24:1	0.18 ± 0.01	0.18 ± 0.01	0.18 ± 0.00	0.08 ± 0.01^&&&^	0.06 ± 0.01^&&&^	0.09 ± 0.01^&&&^	ns	*P <* 0.001	ns
**MUFA**	16.40 ± 1.92	17.83 ± 0.85	17.16 ± 0.56	32.76 ± 3.24^&&&^	38.76 ± 2.69##^,&&&^	27.85 ± 3.48^&&^	ns	*P <* 0.001	ns
18:2 *n*–6 (LA)	16.11 ± 0.64	15.61 ± 0.26	16.57 ± 0.20	15.60 ± 0.069	12.12 ± 3.10	13.78 ± 0.81	ns	ns	ns
20:2 *n*–6	0.45 ± 0.02	0.42 ± 0.02	0.46 ± 0.03	0.26 ± 0.03^&&^	0.30 ± 0.05&	0.22 ± 0.01^&&&^	ns	*P <* 0.001	ns
20:3 *n*–6	1.00 ± 0.11	0.86 ± 0.05	0.92 ± 0.08	0.48 ± 0.05^&&&^	0.55 ± 0.07^&&^	0.57 ± 0.06^&&&^	ns	*P <* 0.001	ns
20:4 *n*–6 (AA)	18.23 ± 1.32	18.19 ± 0.92	18.02 ± 0.34	12.05 ± 1.80^&&^	10.53 ± 0.99#^,&&&^	15.77 ± 1.98	ns	*P <* 0.001	ns
22:4 *n*–6	0.42 ± 0.03	0.54 ± 0.03	0.49 ± 0.03	0.47 ± 0.04	0.50 ± 0.08	0.40 ± 0.03	ns	ns	ns
22:5 *n*–6 (DPA)	0.24 ± 0.00	0.30 ± 0.03	0.27 ± 0.01	0.36 ± 0.11	0.36 ± 0.05	0.46 ± 0.12	ns	ns	ns
***n*–6 LCPUFA**	36.57 ± 1.69	36.06 ± 0.78	36.86 ± 0.45	29.44 ± 1.49&	24.62 ± 3.37#^,&&&^	31.42 ± 1.69&	ns	*P <* 0.001	ns
18:3 *n*–3 (ALA)	0.30 ± 0.01	0.32 ± 0.02	0.35 ± 0.04	0.51 ± 0.08&	0.57 ± 0.11^&&^	0.33 ± 0.06#	ns	*P <* 0.01	ns
20:5 *n*–3 (EPA)	0.48 ± 0.03	0.46 ± 0.03	0.51 ± 0.03	0.10 ± 0.01^&&&^	0.12 ± 0.03^&&&^	0.11 ± 0.01^&&&^	ns	*P <* 0.001	ns
22:5 *n*–3 (DPA)	1.19 ± 0.07	1.17 ± 0.07	1.18 ± 0.10	0.49 ± 0.04^&&&^	0.42 ± 0.08^&&&^	0.44 ± 0.06^&&&^	ns	*P <* 0.001	ns
22:6 *n*–3 (DHA)	7.32 ± 0.18	6.92 ± 0.29	7.67 ± 0.32	3.45 ± 0.65^&&&^	2.75 ± 0.15^&&&^	4.37 ± 0.79^&&&^	ns	*P <* 0.001	ns
***n*–3 LCPUFA**	9.33 ± 0.12	8.90 ± 0.27	9.75 ± 0.35	4.57 ± 0.61^&&&^	3.92 ± 0.31^&&&^	5.28 ± 0.75^&&&^	ns	*P <* 0.001	ns
**Total LCPUFA**	45.89 ± 1.73	44.97 ± 0.92	46.61 ± 0.77	34.01 ± 2.07^&&&^	28.53 ± 3.25#^,&&&^	36.70 ± 2.19^&&^	ns	*P <* 0.001	ns
***n*–6/*n*–3 ratio**	3.92 ± 0.17	4.06 ± 0.12	3.79 ± 0.10	6.69 ± 0.47^&&^	6.52 ± 0.99^&&^	6.41 **±** 0.65^&&&^	ns	*P <* 0.001	ns

Values are mean ± SEM.

*N =* 4–7. Two-way ANOVA with the Tukey post hoc test.

Abbreviations: AA, arachidonic acid; ALA, alpha-linolenic acid; ANOVA, analysis of variance; CD, chow diet; CTL, control diet; DPA, docosapentaenoic acid; HFD, high-fat diet; LA, linoleic acid; LCFA, long-chain SFA; LCPUFA, long-chain PUFA; MCFA, medium-chain SFA; *n*–3 DEF, *n*–3 deficient diet; ns, not significant; PND, postnatal day; SCFA, short-chain SFA.

& *P <* 0.05, ^&&^*P <*^&&&^ 0.01, *P <* 0.001

∗ *P <* 0.05 compared with CTL

# *P <* 0.05

## *P <* 0.01 compared with *n*–3 DEF.

**TABLE 11:** Percentage of selected fatty acids in the liver of female offspring at PND120 after CD or HFD

	CTL	*n*–3 DEF	EPA+DHA	CTL	*n*–3 DEF	EPA+DHA	2-way ANOVA		
	*CD*			*HFD*			*Inter*	*Diet*	*Maternal diet*
14:0	0.51 ± 0.07	0.42 ± 0.06	0.44 ± 0.06	0.25 ± 0.04^&&^	0.34 ± 0.01	0.34 ± 0.06	ns	*P <* 0.01	ns
15:0	0.15 ± 0.01	0.15 ± 0.01	0.16 ± 0.01	0.09 ± 0.00^&&&^	0.09 ± 0.00^&&&^	0.09 ± 0.00^&&&^	ns	*P <* 0.001	ns
16:0	20.20 ± 0.60	17.80 ± 1.01	19.02 ± 0.87	18.11 ± 0.98	18.71 ± 0.42	18.40 ± 0.63	ns	ns	ns
18:0	20.45 ± 0.95	21.48 ± 1.10	20.01 ± 1.43	20.03 ± 1.65	18.81 ± 0.91	19.16 ± 1.17	ns	ns	ns
20:0	0.06 ± 0.00	0.06 ± 0.00	0.06 ± 0.00	0.06 ± 0.00	0.06 ± 0.00	0.06 ± 0.00	ns	ns	ns
22:0	0.12 ± 0.01	0.12 ± 0.01	0.11 ± 0.01	0.13 ± 0.01	0.12 ± 0.01	0.12 ± 0.01	ns	ns	ns
24:0	0.38 ± 0.03	0.37 ± 0.01	0.38 ± 0.02	0.30 ± 0.02&	0.26 ± 0.02^&&&^	0.26 ± 0.01^&&&^	ns	*P <* 0.001	ns
**SCFA**	0.54 ± 0.07	0.43 ± 0.06	0.46 ± 0.06	0.27 ± 0.04	0.35 ± 0.01	0.45 ± 0.16	ns	ns	ns
**MCFA**	40.80 ± 1.01	39.43 ± 0.43	39.19 ± 1.02	38.23 ± 0.67&	37.60 ± 0.51	37.65 ± 0.61	ns	*P <* 0.01	ns
**LCFA**	0.56 ± 0.04	0.55 ± 0.0	0.56 ± 0.03∗∗^,^##	0.49 ± 0.04	0.43 ± 0.03&	0.44 ± 0.02	*P <* 0.05	ns	*P <* 0.01
**Total SFA**	41.90 ± 1.08	40.41 ± 0.43	40.20 ± 1.06	38.98 ± 0.67&	38.39 ± 0.54	38.54 ± 0.49	ns	*P <* 0.01	ns
16:1 *n*–9	0.22 ± 0.01	0.21 ± 0.03	0.22 ± 0.02	0.21 ± 0.03	0.24 ± 0.01	0.22 ± 0.01	ns	ns	ns
16:1 *n*–7	1.74 ± 0.28	1.45 ± 0.36	1.66 ± 0.18	0.60 ± 0.12^&&^	0.83 ± 0.06	0.71 ± 0.08^&&^	ns	*P <* 0.001	ns
18:1 *n*–9	11.13 ± 1.25	10.93 ± 1.05	10.84 ± 0.78	19.72 ± 2.29^&&&^	21.36 ± 0.81^&&&^	20.10 ± 1.01^&&&^	ns	*P <* 0.001	ns
20:1	0.14 ± 0.01	0.16 ± 0.00	0.15 ± 0.01	0.20 ± 0.01^&&^	0.22 ± 0.01^&&^	0.22 ± 0.02^&&&^	ns	*P <* 0.001	ns
22:1	0.05 ± 0.01	0.05 ± 0.01	0.04 ± 0.01	0.05 ± 0.00	0.04 ± 0.00	0.03 ± 0.00	—	—	—
24:1	0.19 ± 0.02	0.19 ± 0.01	0.17 ± 0.01	0.13 ± 0.02^&&^	0.11 ± 0.01^&&^	0.11 ± 0.01^&&^	ns	*P <* 0.001	ns
**MUFA**	15.97 ± 1.57	15.72 ± 1.58	16.13 ± 0.74	22.87 ± 2.48^&&^	24.92 ± 0.86^&&&^	23.55 ± 1.16^&&&^	ns	*P <* 0.001	ns
18:2 *n*–6 (LA)	13.70 ± 0.83	13.70 ± 0.52	13.27 ± 1.07	11.74 ± 0.65	11.52 ± .69&	11.24 ± 0.49&	ns	*P <* 0.01	ns
20:2 *n*–6	0.23 ± 0.01	0.25 ± 0.01	0.25 ± 0.03	0.19 ± 0.01	0.20 ± 0.01&	0.19 ± 0.01^&&^	ns	*P <* 0.001	ns
20:3 *n*–6	0.85 ± 0.06	0.81 ± 0.05	0.85 ± 0.07	0.55 ± 0.04^&&&^	0.55 ± 0.04^&&&^	0.55 ± 0.02^&&&^	ns	*P <* 0.001	ns
20:4 *n*–6 (AA)	16.75 ± 0.82	18.61 ± 1.05	18.17 ± 0.79	16.99 ± 1.52	16.05 ± 0.61	17.31 ± 0.84	ns	ns	ns
22:4 *n*–6	0.36 ± 0.01	0.43 ± 0.02	0.36 ± 0.01#	0.33 ± 0.02	0.36 ± 0.03&	0.36 ± 0.02	ns	*P <* 0.05	ns
22:5 *n*–6 (DPA)	0.26 ± 0.03	0.28 ± 0.03	0.27 ± 0.02	0.57 ± 0.14^&&^	0.50 ± 0.05^&&^	0.54 ± 0.05^&&&^	ns	*P <* 0.001	ns
***n*–6 LCPUFA**	32.35 ± 1.10	34.25 ± 1.33	33.33 ± 1.57	30.59 ± 1.06	29.35 ± 0.08^&&^	30.37 ± 0.61&	ns	*P <* 0.01	ns
18:3 *n*–3 (ALA)	0.32 ± 0.04	0.33 ± 0.03	0.28 ± 0.03	0.32 ± 0.05	0.35 ± 0.04	0.30 ± 0.03	ns	ns	ns
20:5 *n*–3 (EPA)	0.68 ± 0.07	0.49 ± 0.04∗∗	0.61 ± 0.04	0.12 ± 0.02^&&&^	0.13 ± 0.01^&&&^	0.11 ± 0.01^&&&^	ns	*P <* 0.001	ns
22:5 *n*–3 (DPA)	0.90 ± 0.06	0.79 ± 0.04	0.86 ± 0.04	0.39 ± 0.02^&&&^	0.41 ± 0.06^&&&^	0.43 ± 0.03^&&&^	ns	*P <* 0.001	ns
22:6 *n*–3 (DHA)	7.86 ± 0.33	7.98 ± 0.39	8.54 ± 0.36	6.70 ± 0.88	6.44 ± 0.49&	6.66 ± 0.55^&&^	ns	*P <* 0.01	ns
***n*–3 LCPUFA**	9.78 ± 0.30	9.62 ± 0.32	10.33 ± 0.34	7.56 ± 0.80^&&^	7.35 ± 0.40^&&^	7.54 ± 0.52^&&&^	ns	*P <* 0.001	ns
**Total LCPUFA**	42.13 ± 1.17	43.87 ± 1.61	43.66 ± 1.59	38.15 ± 1.82	36.69 ± 0.35^&&&^	37.91 ± 0.82^&&^	ns	*P <* 0.001	ns
***n*–6/*n*–3 ratio**	3.32 ± 0.15	3.56 ± 0.08	3.24 ± 0.20	4.15 ± 0.33&	4.04 ± 0.23	4.16 ± 0.34^&&^	ns	*P <* 0.01	ns

Values are mean ± SEM.

*N =* 4–7.

Abbreviations: AA, arachidonic acid; ALA, alpha-linolenic acid; ANOVA, analysis of variance; CD, chow diet; CTL, control diet; DPA, docosapentaenoic acid; HFD, high-fat diet; LA, linoleic acid; LCFA, long-chain SFA; LCPUFA, long-chain PUFA; MCFA, medium-chain SFA; *n*–3 DEF, *n*–3 deficient diet; ns, not significant; PND, postnatal day; SCFA, short-chain SFA.

∗*P <* 0.05, ∗∗∗*P <* 0.001 vs. CTL. ^###^*P* < 0.001 vs. *n*–3 DEF.

∗∗ P < 0.01

# *P <* 0.05

## *P <* 0.01

& vs. CD. Two-way ANOVA with the Tukey post hoc test.

Concerning cholesterol levels in the offspring, HFD significantly increased plasma and hepatic total cholesterol in males of all groups (*P <* 0.001; Figure 8C; Supplemental Figure 8C).

Thus, it suggests that *1*) TGs were greatly stored in the liver of males under HFD compared with females, *2*) the enrichment in EPA, DHA, and AA in the maternal diet modulates hepatic cholesterol content in both males and females at weaning, and *3*) influences cholesterol handling under HFD in males.

### Early maternal diet influences the hepatic expression of genes involved in lipid synthesis in the offspring

We evaluated the expression of several lipid-related genes in the liver at PND21 and PND120. The expression of genes involved in *n*–3 and *n*–6 synthesis was significantly upregulated in both sexes at weaning, likely adapting to maternal supply of *n*–3 LCPUFA (Supplemental Figure 7E, F). Some genes involved in FA synthesis and intracellular transport, such as *Fas, Acaca, Fabp4,* and *Lxra*, were particularly upregulated. This corresponds with elevated C24:0 and C24:1 levels in the liver (Supplemental Table 4), suggesting that maternal diet promotes lipogenesis at weaning. At adulthood, increased expression of *CD36, Acsl1, and Dgat2* in males of the EPA+DHA group suggests enhanced TG synthesis, whereas the decreased expression of *Cpt1a* indicated reduced FA oxidation (Supplemental Figure 8B). In females, an increased expression of *Acsl1, Cpt1a*, and *Mttp* implies enhanced FA oxidation and lipid excretion (Supplemental Figure 8F), which may be linked to the decrease of hepatic TG storage and the plasma TG/cholesterol increase (Supplemental Figure 8A–D).

After HFD, lipid transporter expression tended to be globally decreased in both sexes as well as lipid synthesis in males (Figure 8E, F). *Lrp1, Ldlr, and Lpl* expression were unchanged across groups, suggesting an unaffected lipoprotein uptake (Figure 8G). Despite that, *Acsl1* and *Dgat2* were downregulated, *Soat2* was not changed, suggesting cholesterol esterification in the liver, and *Cpt1a* was decreased, suggesting a lower FA oxidation in the CTL group. Conversely, in males of the EPA+DHA group, *Soat2* trended down, and *Cpt1a* trended up, supporting the lower increase of TG and liver cholesterol content. In females, gene expression was particularly affected in the EPA+DHA group (Figure 8H). Despite the fact that *Cpt1a* was decreased in females of the EPA+DHA group, the constant expression of *Dgat2* might contribute to the apparently best management of TG and cholesterol in females under HFD.

### Exploratory behavior is slightly modified in offspring according to maternal *n*–3-deficient diet or EPA+DHA+AA–enriched diet according to sex

**FIGURE 9:**
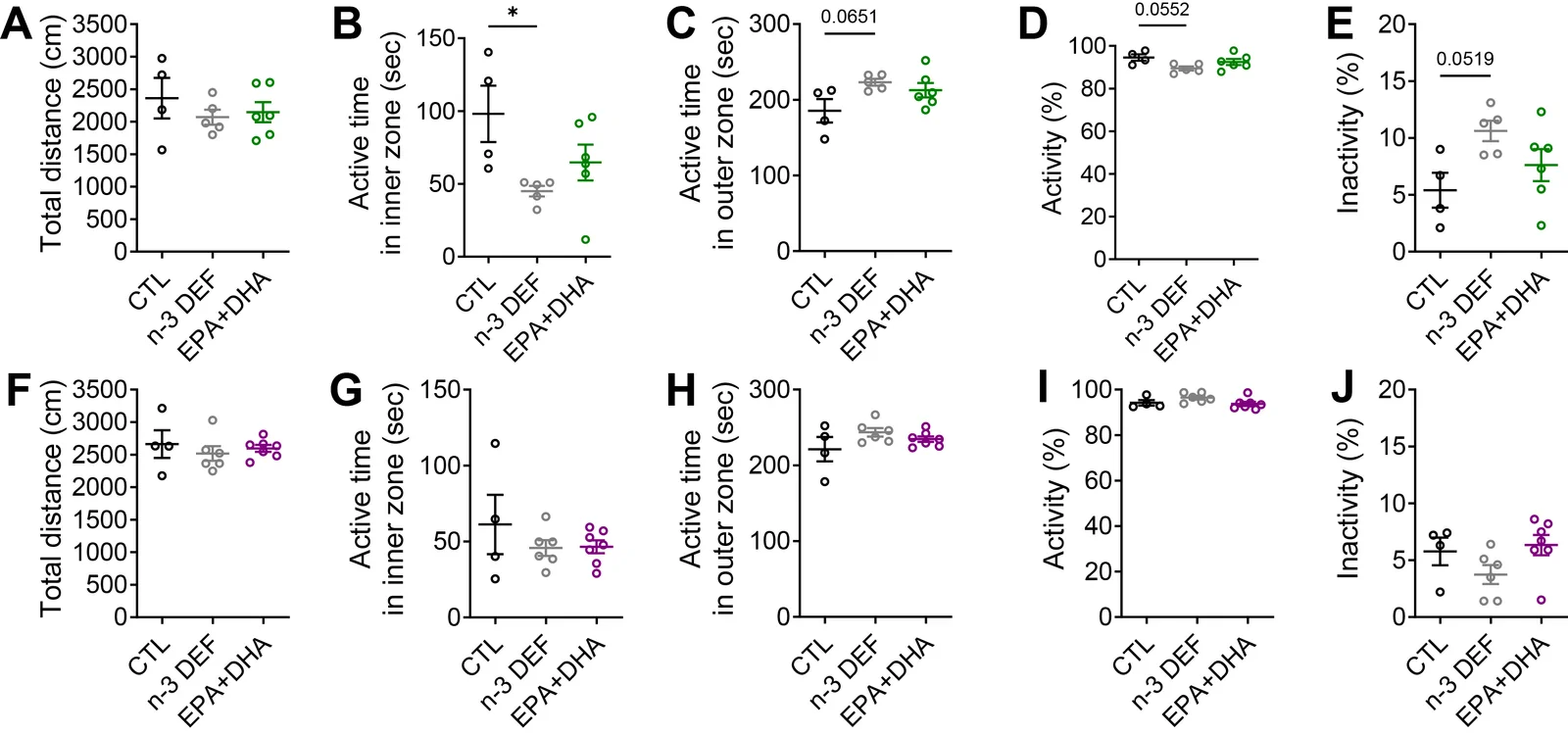
Locomotor activity and anxiety measurements in the open field on postnatal day (PND) 80. Total distance traveled (A, F), active time in inner (B, G) and outer (C, H) zone, and percentages of activity (D, I) and inactivity (E, J) have been measured during 5 min in males (A–E) and females (F–J) at PND80. Black circle, CTL; gray circle, *n*–3 DEF; colored circle, EPA+DHA; Opened circle, high-fat diet. Green, male; purple, female. Data are mean ± SEM or individual values (*n* = 4–7). ∗ *P <* 0.05, ∗∗ *P <* 0.01, ∗∗∗ *P <* 0.001 compared with CTL, 2-way ANOVA with Sidak posttest, unpaired *t*-test. ANOVA, analysis of variance; CTL, control diet; EPA+DHA, EPA-, DHA-, and AA-enriched diet; *n*–3 DEF, *n*–3 deficient diet.

We assessed the exploratory behavior of the offspring using an open-field test at PND80–85. Under CD, no behavioral differences were observed between groups (Figure 9A, Supplemental Figure 6). However, under HFD, males of the *n*–3 DEF groups showed reduced activity in the inner zone and signs of decreased exploration (Figure 9B–E), suggesting potential anxiety-like behavior. This was not the case for the animals of the EPA+DHA group.

## Discussion

In a maternal PR animal model, we assessed how a diet enriched in LCPUFA of *n*–3 type during gestation and lactation only affects milk and offspring hypothalamic FA composition at various developmental stages. An *n*–3 LCPUFA–deficient diet significantly decreased *n*–3 LCPUFA levels in mother RBC membrane and milk, as well as in the hypothalamus and the liver of both male and female offspring. In contrast, dietary enrichment with EPA, DHA, and AA increased *n*–3 LCPUFA levels, particularly DHA, in these tissues. Correlation analyses and a predictive model identified mother RBC *n*–3 LCPUFA content and the *n*–6/*n*–3 ratio as strong indicators of milk and developing brain FA composition.

Although a slight, nonsignificant increase in mortality was noted in the EPA+DHA group, our findings align with previous studies that suggest that excessive *n*–3 LCPUFA, with or without AA, can impact pup viability due to potential lipid peroxidation when administered directly [21,[24], [25], [26]]. In our model, however, LCPUFAs were supplied via the maternal diet and were not administered directly to pups, thus avoiding perinatal toxicity. Importantly, to the best of our knowledge, no adverse effects on newborn health from maternal *n*–3 supplementation have been reported in human studies [27]. Furthermore, the number of female pups at birth was significantly higher in the *n*–3 DEF group. The influence of diet, particularly the presence of *n*–3 or *n*–6 LCPUFAs, on the sex of offspring has already been described [28,29].

Offspring brain FA composition is largely determined by FA transfer through the placenta and early-life nutrition. In LBW, inadequate in utero LCPUFA supply may impair development [4,5,30]. Supported by earlier studies [9,10,31], our results suggest that enhancing maternal dietary FA, which results particularly in milk DHA and AA increases, can potentially help to reduce developmental deficits linked to PR, which may be due to an altered hepatic lipid metabolism in PR mothers [7,32]. In PR dams, delayed mammary gland maturation and altered milk FA profiles further contribute to EUGR and reduced offspring postnatal brain lipid accretion [8,10,[31], [32], [33]].

Despite high dietary LA, we observed low maternal ALA and EPA levels in the *n*–3 DEF group, indicating preferential *n*–6 LCPUFA synthesis. LA is known to compete with ALA for endogenous conversion to EPA and DHA and to inhibit incorporation of DHA and EPA into tissues [34]. Thus, in accordance with the literature, high dietary LA generally results in low *n*–3 LCPUFA status. This emphasizes the importance of including preformed EPA and DHA in the maternal diet to support optimal neural development. The European Academy of Pediatrics and the Child Health Foundation now recommend that infant formulas include both DHA and AA to maintain balanced intake and avoid impaired AA brain accretion, thereby supporting neurodevelopment [35]. Our study highlights further the importance of the *n*–6/*n*–3 ratio as a predictor of brain DHA incorporation, associated with individual FA species.

Although the benefits of *n*–3 LCPUFA supplementation during pregnancy or lactation remain inconclusive aside from improved visual outcomes [[36], [37], [38]], DHA remains important for brain maturation. Given the difficulty of direct measurement of brain FA in humans, maternal RBC membranes serve as practical biomarkers, reflecting both dietary intake and endogenous LCPUFA synthesis. In our model, maternal RBC FA levels changed within a week of dietary intervention, consistent with the FA profile of the diet. Long-term human dietary interventions such as *n*–3 LCPUFA supplementation modulate the FA composition of RBC membrane [39]. This is especially true for FA with absent or marginal endogenous synthesis, such as LA, ALA, EPA, and DHA [[40], [41], [42]]. Measuring the FA profile of RBC membrane may surpass the accuracy of food frequency questionnaires by accounting for genetic differences in FA metabolism [15].

Key FA—oleate, MUFA, LA, DHA, *n*–3 LCPUFA, and *n*–6/*n*–3 ratio—were found to be predictive of offspring FA composition. Using PLS regression, we identified DHA, *n*–3 LCPUFA, and the *n*–6/*n*–3 ratio as the most relevant predictors of brain DHA content. Once validated, the membrane RBC FA profile could provide a noninvasive method, particularly when milk sampling is impractical. To achieve this, we would need to replicate our observations in an independent study to validate the predictive algorithms and to use a continuous range of DHA concentrations—from low to high—specifically filling the 12%–14% DHA gap observed in our study, thereby strengthening the robustness of our findings. This aligns with previous observations in baboons demonstrating the value of such an approach [41].

AA is primarily provided through food and accumulates throughout pregnancy in maternal adipose tissue, supplying babies with it through breast milk [35]. Interestingly, AA is not a strong predictor of brain DHA levels and may not be a reliable biomarker. Although elevated in maternal milk, AA increased in the offspring hypothalamus only under *n*–3 deficiency, possibly because of reduced competition from DHA. In addition, deprivation of *n*–3 LCPUFA may also prolong the half-life of DHA to conserve it [43,44], explaining its moderate presence even in the deficient group.

The FA modulation exhibits variability depending on factors such as brain region, animal strain, species, timing of LCPUFA supplementation, and age [[45], [46], [47]]. Other studies using a high LA diet during the gestation and lactation, as well as postweaning in offspring, evidenced relatively small changes in FA composition in brain or blood in young offspring [48,49]. We detected differences in neonatal and weanling brain FAs, which largely normalized by PND120. However, DPA remained elevated in the *n*–3 DEF offspring, possibly compensating for low DHA levels. Previous studies have indicated that DPA *n*–6 can replace DHA in the absence of the latter [50]. The potential impact of this substitution warrants further investigation because Moriguchi et al. [51] demonstrated in a rat model of *n*–3 deprivation, an altered escape latency in rats with a brain DHA level of 7.6% of total FA but not in those with a level of 10.6%, suggesting a potential functional threshold.

Despite the normalization of certain brain FAs, early-life exposure to unbalanced maternal diets may program offspring for long-term metabolic and cognitive vulnerability [48,[52], [53], [54], [55]]. High intake of SFA during pregnancy is associated with pregnancy complications and poor birth outcomes, including IUGR. The SFA biomagnification process could help to satisfy AA demands in fetal circulation and DHA in the brain [56]. An adequate concentration of MUFA is also fundamental because a nonoptimal level has been associated with a high risk of preterm delivery and LBW [56]. The consumption of a diet enriched in MUFA could benefit DHA metabolism, thus not interfering with endogenous conversion of ALA to DHA [34]. From day 60 to 120, we challenged offspring with either standard chow or HFD. Although the HFD duration was insufficient to induce obesity, this allowed us to isolate the effects of the maternal diet in early life on the metabolism and behavior of the offspring. Importantly, a maternal *n*–3-enriched diet enhanced the metabolic resiliency of offspring at a later age. It improved insulin sensitivity, modulated hepatic lipid metabolism, and altered hepatic gene expression involved in lipid metabolism, especially in females. This led to better lipid oxidation and reduced TG accumulation under HFD, especially in males.

In conclusion, the maternal diet enriched with EPA+DHA+AA positively influences offspring FA composition and may support cognitive development. The levels of *n*–3 LCPUFA and *n*–6/*n*–3 ratio in the mother RBC are effective, noninvasive predictors of hypothalamic DHA levels. These findings advocate the use of maternal dietary strategies to improve neurodevelopmental outcomes and suggest a possible role in partially reducing the effects of obesogenic stressors in offspring.

## Author contributions

The authors’ responsibilities were as follows – VSM: designed and performed experiments, collected and analyzed data, and wrote and revised the manuscript; BC, IG, AG: performed animal experiments; AD-S: performed gas chromatography analysis; J-CM: performed PLS analysis, analyzed data, and revised the manuscript; PP: designed experiments, analyzed data, and wrote and revised the manuscript.

## Ethics approval

All animal experiments were approved by the Animal Ethics Committee of Pays de La Loire under reference APAFIS#13253-2020060814013364 v1.

## Data availability

Data described in the manuscript, codebook, and analytic code will be made available upon request pending application and approval.

## Funding

Funding supporting this work includes a Postdoctoral Fellowship from the Fondation pour la Recherche Médicale (ARF20170938730; VSM), the local grant from the Fondation SantéDige (VSM), and the regional grant RFI-Food For Tomorrow (PP).

## Declaration of generative AI and AI-assisted technologies in the writing process

During the preparation of this work, the authors used DeepL to edit the manuscript for clarity and language. After using these tools, the authors reviewed and edited the content as needed and take full responsibility for the content of the publication.

## Conflict of interest

The authors report no conflicts of interest.
