# Supplementary material for: Maternal Diet Enrichment in n–3 Long-Chain Polyunsaturated Fatty Acids during Pregnancy and Lactation Improves Their Transfer from the Mother to the Offspring and Counterbalances High-Fat Diet-Induced Alterations in a Sex-Dependent Manner in Rats
Source: Curr Dev Nutr. 2026 Aug 7;10(9):109508. doi: 10.1016/j.cdnut.2026.109508 (PMC13545725; doi:10.1016/j.cdnut.2026.109508)
Supplement: Supplementary file 1 [file mmc1.docx]

Maternal diet enrichment in n-3 long-chain polyunsaturated fatty acids during pregnancy and lactation improves their transfer from the mother to the offspring and counterbalances high-fat diet-induced alterations in a sex-dependent manner in rats

Moullé et al

**Supplemental material**

**MATERIAL & METHODS**

*Biological sampling collection*

Blood samples from pregnant and lactating rats were collected on alert animals by a tail snip in EDTA-tubes (Pfizer-Centravet, Plancoët, France) at D6, D13, D19, D27, D34, and D41. Blood samples were centrifuged at 10 000 rpm for 2 min at 4°C, and plasma and mother RBC membrane were collected in Eppendorf before storage at -20 C until analysis.

Milk samples were collected from lactating dams at L6, L13, and L19, as described in (Martin Agnoux et al., 2015). Briefly, dams were separated from their pups and received an intraperitoneal injection of oxytocin (1 unit of Syntocinon®; Sigma-Tau, Ivry-sur-Seine, France) to stimulate milk ejection. After 15-20 min, dams were anesthetized with 4% of isoflurane, and by applying manual pressure to nipples, about 200 to 400 μL of milk were collected before storage at -20C. Since we have seen a deleterious effect of pup separation from their mother on offspring growth, we performed this milk collection on a separate cohort and did not keep pups for further analysis.

At sacrifice, blood samples were collected by an intra-cardiac puncture in EDTA-tubes before sacrifice on anaesthetized animals.

*Triglyceride and cholesterol content in liver*

Around 50 mg of piece of liver were crushed in saline with a zirconium marble in PreCellys. A mixture of chloroform:methanol (2:1) was added then tubes were centrifuged 15 min at 2500g. The organic phase was withdrawn and evaporated under the fume cupboard during few hours. The bottom was resuspended in isopropanol:triton X-100 (9:1) and assayed with colorimetric test for triglyceride and cholesterol detection (Triglycerides FS®; Cholesterol FS®; Diasys, Holzeim, Germany).

*Biochemical analysis*

Blood glucose was measured on glucometer (ACCU-CHEK® PERFORMA, Roche Diagnostics France, Meylan, France) by a tail snip. Plasma insulin, leptin and adiponectin were assayed by ELISA (ALPCO, Salem, USA); plasma triglycerides and total cholesterol were assayed by colorimetric tests cited above; milk proteins (PierceTM BCA Protein Assay Kit; ThermoFisher Scientific, Waltham, MA, USA), lactose (K-LACGAR®; Megazyme, Bray, Ireland) and cholesterol were assayed by colorimetric or enzymatic assays after milk dilution, following manufacturers’ instructions.

**BIBLIOGRAPHY**

Martin Agnoux, A., Antignac, J. P., Boquien, C. Y., David, A., Desnots, E., Ferchaud-Roucher, V., Darmaun, D., Parnet, P., & Alexandre-Gouabau, M. C. (2015). Perinatal protein restriction affects milk free amino acid and fatty acid profile in lactating rats : Potential role on pup growth and metabolic status. *J Nutr Biochem*, *26*(7), 784‑795. https://doi.org/10.1016/j.jnutbio.2015.02.012

**Supplemental Figure 1. Plasma parameters and adiposity follow-up of dams during gestation and lactation.** Blood glucose (A), plasma insulin (B), leptin (C), adiponectin (D), and triglyceride (E) levels have been followed during gestation (from D1 to D21) and lactation (from D22 to D42). At weaning, mothers have been sacrificed and mesenteric (F) and perirenal (G) white adipose tissues, mammary gland (H), and liver (I) have been weighed. Triglyceride (J) and cholesterol (K) contents have been assayed in the liver. Solid line, CTL; Grey line, n-3DEF; Dotted black line, EPA+DHA. Vertical dotted line at D21 indicates delivery and at D42 indicates weaning. CTL, control. N-3 DEF, n-3 deficient diet. EPA+DHA, EPA, DHA and AA enriched diet. WAT, white adipose tissue; TG, triglycerides; CHOL, cholesterol. Data are mean ± SEM or individual values (n=3-11).

**Supplemental figure 2. Selected fatty acid percentage in offspring hypothalamus at PND21.** Evolution of n-3 LCPUFA (A), n-6 LCPUFA (B), n-6/n-3 ratio (C), DHA (D), LA (E), AA (F), n-6 DPA (G), and 18:1 n-9/18:2 n-6 ratio (H) in hypothalamus of male and female offspring at birth. Black circle, CTL; Grey circle, n-3 DEF; Opened circle, EPA+DHA. CTL, control. N-3 DEF, n-3 deficient diet. EPA+DHA, EPA, DHA and AA enriched diet. LCPUFA, polyunsaturated fatty acid. DHA, docosahexaenoic acid. LA, linoleic acid. AA, arachidonic acid. Data are mean ± SEM and individual values (n=4-10). *p<0.05, **p<0.01, ***p<0.001, 2-way ANOVA with Tukey post-test.

**Supplemental Figure 3.** Predicted DHA in offspring hypothalamus at PND21 from maternal RBC membrane FA composition and FA milk composition. Differences among measured and predicted DHA in offspring hypothalamus at G19 (A), L6 (B), and L13 (C), according to the predictive PLS algorithm. Correlations between measured and predicted DHA in offspring hypothalamus at G9 (D), L6 (E), and L13 (F). The predictive equation calculated for the offspring hypothalamus from mother RBC fatty acids at G19 was as follows: [Pred%hypoDHA = (-0.120348 *RBC C16:0 G19) – (0.039965 *RBC C18:0 G19) – (0.0378928 *RBC SFA G19) + (0.389819 * RBC C22:1 G19) – (0.0999879 * RBC C18:2n6 G19) – (0.534511 * RBC C22:5n6 G19) + (1.16242 * RBC C18:3n3 G19) – (1.45841 * RBC C20:5n3 G19) + (0.498747 * RBC C22:6n3 G19) + (0.323422 * RBC LCPUFAn3 G19) – (0.018243 * RBC n6/n3 ratio G19) +21.4195], at L6 [Pred%hypoDHA = ( -2.52602 * RBC C12:0 L6) – (8.27924 *RBC C20:0 L6) + (0.304856 * RBC C18:1n7 L6) – (0.118181 * RBC C22:1 L6) + (0.370521 * RBC C24:1 L6) – (0.0756837 * RBC C18:2n6 L6) – (3.83211 * RBC C18:3n6 L6) – (3.96969 * RBC C20:2n6 L6) – (2.34125 * RBC C20:3n6 L6) – (0.674357 * RBC C22:5n6 L6) – (0.0502067 * RBC LCPUFAn6 L6) – (0.815973 * RBC C20:5n3 L6) + (0.884166 * RBC C22:5n3 L6) + (0.502417 * RBC C22:6n3 L6) + (0.230227 * RBC LCPUFAn3 L6) – (0.00609789 * RBC n6/n3 ratio L6) +16.8319], and at L13 [Pred%hypoDHA = (2.82027 * RBC C12:0 L13) + (1.76097 * RBC C14:0 L13) + (2.52434 * RBC C15:0 L13) – (0.153006 * RBC C18:0 L13) + (0.906336 * RBC C16:1n9 L13) + (0.375553 * RBC C16:1n7 L13) + (0.219012 * RBC C18:1n7 L13) + (1.91735 * RBC C20:1 L13) + (0.156062 * RBC C22:1 L13) + (0.284661 * RBC C24:1 L13) + (0.0330093 * RBC MUFA L13) – (0.0559647 * RBC C18:2n6 L13) – (3.29423 * RBC C20:2n6 L13) – (0.810686 * RBC C20:3n6 L13) – (0.398996 * RBC C22:4n6 L13) – (0.332697 * RBC C22:5n6 L13) – (0.020429 * RBC LCPUFAn6 L13) + (0.242132 * RBC C22:6n3 L13) + (0.193236 * RBC LCPUFAn3 L13) – (0.018656 * RBC LCPUFAtotaux L13) – (0.00624814 * RBC n6/n3 ratio L13) +12.9818]. Black circle, CTL; Grey circle, n-3 DEF; Opened circle, EPA+DHA. CTL, control. N-3 DEF, n-3 deficient diet. EPA+DHA, EPA, DHA and AA enriched diet. LCPUFA, long-chain polyunsaturated fatty acid. DHA, docosahexaenoic acid. Data are mean ± SEM and individual values (n=4).

**Supplemental Figure 4. Consequences of high-fat diet on postnatal growth of male and female offspring.** Body weight (BW; A,B) at postnatal (PND) 61 in male (A) and female (B). BW gain (C,D) from PND61 to PND120 under high-fat diet in male (C) and female (D). Liver (E,I), subcutaneous (F,J), mesenteric (G,K), and perirenal (H,L) white adipose tissues have been weighed at PND120 in male (E-H) and female (I-L). Black circle, CTL; Grey circle, n-3 DEF; Coloured circle, EPA+DHA. Green, male; purple, female. CTL, control. N-3 DEF, n-3 deficient diet. EPA+DHA, EPA, DHA and AA enriched diet. Data are mean ± SEM or individual values (n = 9-12). * p < 0.05, ** p < 0.01, *** p < 0.001 vs. CTL, 2-way ANOVA with Sidak post-test, unpaired t-test.

**Supplemental Figure 5. Glucose tolerance in male and female offspring under chow diet at postnatal day (PND) 100.** Basal blood glucose (A,F), blood glucose follow-up (B,G), and area under the curve (AUC; C,H) after oral glucose gavage (2 g/kg) in male (A-C) and female (F-H). Plasma insulin in response to glucose (D,I) and AUC (E,J) in male (D,E) and female (I,J) are presented. Solid line, CTL; Grey line, n-3DEF; Coloured line, EPA+DHA. Black circle, CTL; Grey circle, n-3 DEF; Coloured circle, EPA+DHA. Green, male; purple, female. CTL, control. N-3 DEF, n-3 deficient diet. EPA+DHA, EPA, DHA and AA enriched diet. Data are mean ± SEM or individual values (n = 4–6). * p < 0.05, ** p < 0.01, *** p < 0.001 vs. CTL, 2-way ANOVA with Sidak post-test, unpaired t-test.

**Supplemental Figure 6. Locomotor activity and anxiety measurements in open field at postnatal day (PND) 80.** Total distance travelled (A,F), active time in inner (B,G) and outer (C,H) zones, and percentages of activity (D,I) and inactivity (E,K) have been measured during 5 minutes in male (A-E) and female (F-J) at PND80. Black circle, CTL; Grey circle, n-3 DEF; Coloured circle, EPA+DHA, Opened circle, high-fat diet. Green, male; purple, female. CTL, control. N-3 DEF, n-3 deficient diet. EPA+DHA, EPA, DHA and AA enriched diet. Data are mean ± SEM or individual values (n = 6-9). * p < 0.05, ** p < 0.01, *** p < 0.001 vs. CTL, 2-way ANOVA with Sidak post-test, unpaired t-test.

**Supplemental Figure 7. Plasma and liver levels of triglycerides and cholesterol and gene expression of lipid pathways in male and female under chow diet at postnatal day (PND) 21.** Triglycerides (A,B) and cholesterol (C,D) have been measured in plasma (A,C) and liver (B,D) in male and female at PND21. Liver gene expression in male (E) and female (F) at PND21. Black circle, CTL; Grey circle, n-3 DEF; Coloured circle, EPA+DHA, Opened circle, high-fat diet. Green, male; purple, female. CTL, control. N-3 DEF, n-3 deficient diet. EPA+DHA, EPA, DHA and AA enriched diet. Data are mean ± SEM or individual values (n = 6-13). * p < 0.05, ** p < 0.01, *** p < 0.001 vs. CTL, 2-way ANOVA with Sidak post-test, unpaired t-test.

**Supplemental Figure 8. Plasma and liver levels of triglycerides and cholesterol and gene expression of lipid pathways in male and female under chow diet at postnatal day (PND) 120.** Triglycerides (A,B) and cholesterol (C,D) have been measured in plasma (A,C) and liver (B,D) in male and female at PND120. Liver gene expression in male (E) and female (F) at PND120. Black circle, CTL; Grey circle, n-3 DEF; Coloured circle, EPA+DHA, Opened circle, high-fat diet. Green, male; purple, female. CTL, control. N-3 DEF, n-3 deficient diet. EPA+DHA, EPA, DHA and AA enriched diet. Data are mean ± SEM or individual values (n = 5–10). * p < 0.05, ** p < 0.01, *** p < 0.001 vs. CTL, 2-way ANOVA with Sidak post-test, unpaired t-test.

**Supplemental Table 1.** Composition of Control, n-3 DEF, and EPA+DHA diets.

| g/100g | CTL | n-3 DEF | EPA+DHA |
| --- | --- | --- | --- |
| Casein | 8 | 8 | 8 |
| L-Cystine | 0.3 | 0.3 | 0.3 |
| Corn starch | 47.32 | 47.32 | 47.32 |
| Maltodextrine | 15.72 | 15.72 | 15.72 |
| Sucrose | 11.91 | 11.91 | 11.91 |
| Cellulose | 5 | 5 | 5 |
| Corn oil | - | 7 | - |
| Colza oil | 7 | - | 5.495 |
| Arasco | - | - | 0.560 |
| Coconut oil | - | - | 0.210 |
| Omegavie® tuna oil 25 DHA TG^a^ | - | - | 0.735 |
| t-butylhydroquinone | 0.0014 | 0.0014 | 0.0014 |
| Mineral mix AIN 93 G^b^ | 3.5 | 3.5 | 3.5 |
| Vitamin mix AIN 93 Vx^c^ | 1 | 1 | 1 |
| Choline bitartrate | 0.25 | 0.25 | 0.25 |
| Proteins | 8.3 | 8.3 | 8.3 |
| Carbohydrates | 74.95 | 74.95 | 74.95 |
| Lipids | 7 | 7 | 7 |

^a^ Omegavie® tuna oil 25 DHA TG = tuna oil rich in n-3 fatty acids in triglyceride form.

^b^ Mineral mix AIN 93 G = Calcium Carbonate 35.7%, Monopotassium Phosphate 19.6%, Potassium Citrate Monohydrate 7.078%, Sodium Chloride 7.4%, Potassium Sulfate 4.66%, Magnesium Oxide 2.4%, Ferric Citrate 0.606%, Zinc Carbonate 0.165%, Manganese Carbonate 0.063%, Copper Carbonate 0.03%, Potassium Iodate 0.001%, Sodium Selenate, Anhydrous 0.00103%, Ammonium Molybdate.4H2O 0.000795%, Sodium Metasilicate.9H2O 0.145%, Chromium Potassium Sulfate.12H2O 0.0275%, Lithium Chloride 0.00174%, Boric Acid 0.008145%, Sodium Fluoride 0.00635%, Nickel Carbonate 0.00318%, Ammonium Vanadate 0.00066%, Powdered Sugar 22.1%.

^c^ Vitamin mix AIN 93 Vx (gm/kg) = Nicotinic Acid 3.00, D-Calcium Pantothenate 1.60, Pyridoxine HCl 0.70, Thiamine HCl 0.60, Riboflavin 0.60, Folic Acid 0.20, D-Biotin 0.02, Vitamin B12 (0.1% triturated in mannitol) 2.50, a-Tocopherol Powder (250 U/gm) 30.00, Vitamin A Palmitate (250,000 U/gm) 1.60, Vitamin D3 (400,000 U/gm) 0.25, Phylloquinone 0.075, Powdered Sucrose 959.655.

**Supplemental Table 2.** Selected fatty acid composition in percentage of total fatty acid in Control, n-3 DEF, and EPA+DHA diets.

|  | CTL | n-3 DEF | EPA+DHA |
| --- | --- | --- | --- |
| 10:0 | 0.06 | 0.05 | 0.24 |
| 12:0 | 0.06 | 0.05 | 1.51 |
| 14:0 | 0.16 | 0.14 | 1.07 |
| 15:0 | 0.04 | 0.03 | 0.14 |
| 16:0 | 5.49 | 12.19 | 7.30 |
| 18:0 | 1.95 | 2.32 | 2.85 |
| 20:0 | 0.52 | 0.44 | 0.52 |
| 22:0 | 0.29 | 0.17 | 0.37 |
| 24:0 | 0.12 | 0.19 | 0.20 |
| **SCFA** | **0.27** | **0.24** | **2.82** |
| **MCFA** | **7.49** | **14.54** | **10.29** |
| **LCFA** | **0.93** | **0.79** | **1.09** |
| **Total SFA** | **8.69** | **15.57** | **14.19** |
| 16:1 n-9 | 0.05 | 0.05 | 0.07 |
| 18:1 n-9 | 58.96 | 30.25 | 50.30 |
| 18:1 n-7 | 2.85 | 0.41 | 2.54 |
| 20:1 | 1.23 | 0.30 | 1.14 |
| 22:1 | 0.23 | 0.27 | 0.33 |
| 24:1 | 0.12 | 0.01 | 0.16 |
| **Total MUFA** | **63.66** | **31.42** | **55.15** |
| 18:2 n-6 (LA) | 19.78 | 51.86 | 16.74 |
| 20:2 n-6 | 0.08 | 0.04 | 0.14 |
| 20:4 n-6 (ARA) | 0.03 | 0.02 | 3.52 |
| 22:4 n-6 | 0.03 | 0.03 | 0.07 |
| 22:5 n-6 (DPA) | 0.01 | 0.01 | 0.18 |
| **n-6 LCPUFA** | **19.93** | **51.96** | **21.21** |
| 18:3 n-3 (ALA) | 7.72 | 1.03 | 6.25 |
| 20:5 n-3 (EPA) | - | - | 0.62 |
| 22:5 n-3 (DPA) | - | - | 0.13 |
| 22:6 n-3 (DHA) | - | - | 2.34 |
| **n-3 LCPUFA** | **7.72** | **1.05** | **9.44** |
| **Total LCPUFA** | **27.64** | **53.01** | **30.65** |
| **n-6/n-3 ratio** | **2.6** | **49.6** | **2.3** |

SCFA, short-chain saturated fatty acid. MCFA, medium-chain saturated fatty acid. LCFA, long-chain saturated fatty acid. MUFA, monounsaturated fatty acid. LA, linoleic acid. ARA, arachidonic acid. DPA, docosapentaenoic acid. ALA, alpha-linolenic acid. EPA, eicosapentaenoic acid. DHA, docosahexaenoic acid. LCPUFA, long-chain polyunsaturated fatty acid.

**Supplemental Table 3.** Average percentage of fatty acid in cortex of male and female offspring at PND21-22.

|  | CTL | n-3 DEF | EPA+DHA | CTL | n-3 DEF | EPA+DHA | 2-way Anova | | |
| --- | --- | --- | --- | --- | --- | --- | --- | --- | --- |
|  | *Male* | | | *Female* | | | *Inter* | *Sex* | *Maternal diet* |
| 14:0 | 0.60±0.02 | 0.57±0.02 | 0.60±0.03 | 0.56±0.02 | 0.52±0.03 | 0.57±0.01 | ns | ns | ns |
| 15:0 | 0.08±0.00 | 0.06±0.00 | 0.08±0.00 | 0.07±0.01 | 0.06±0.00 | 0.09±0.00^##^ | ns | ns | p<0.001 |
| 16:0 | 26.9±0.2 | 26.8±0.4 | 26.0±0.3 | 26.2±0.2 | 27.1±0.6 | 26.3±0.3 | ns | ns | ns |
| 18:0 | 20.1±0.2 | 20.1±0.4 | 20.3±0.2 | 19.8±0.2 | 19.7±0.6 | 20.1±0.2 | ns | ns | ns |
| 20:0 | 0.11±0.01 | 0.13±0.02 | 0.14±0.01 | 0.16±0.02 | 0.15±0.03 | 0.14±0.01 | ns | ns | ns |
| 22:0 | 0.06±0.01 | 0.10±0.02 | 0.12±0.02 | 0.15±0.02 | 0.14±0.03 | 0.11±0.02 | ns | p<0.05 | ns |
| 24:0 | 0.10±0.02 | 0.13±0.03 | 0.21±0.02 | 0.20±0.03 | 0.16±0.05 | 0.20±0.03 | ns | ns | ns |
| **SCFA** | **0.60±0.02** | **0.57±0.02** | **0.60±0.03** | **0.56±0.02** | **0.52±0.03** | **0.57±0.01** | **ns** | **ns** | **ns** |
| **MCFA** | **47.1±0.1** | **46.9±0.2** | **46.3±0.1** | **46.1±0.2^&^** | **46.8±0.3** | **46.5±0.2** | **p<0.05** | **ns** | **ns** |
| **LCFA** | **0.28±0.05** | **0.37±0.07** | **0.47±0.05** | **0.51±0.05** | **0.45±0.10** | **0.45±0.06** | **ns** | **p=0.08** | **ns** |
| **SFA** | **48.0±0.1** | **47.9±0.2** | **47.4±0.2** | **47.2±0.2^&^** | **47.8±0.2** | **47.5±0.2** | **p<0.05** | **ns** | **ns** |
| 16:1 n-9 | 0.47±0.02 | 0.41±0.02 | 0.46±0.01 | 0.50±0.02 | 0.40±0.01** | 0.44±0.01 | ns | ns | p<0.001 |
| 16:1 n-7 | 0.59±0.02 | 0.57±0.02 | 0.59±0.01 | 0.59±0.02 | 0.56±0.01 | 0.58±0.01 | ns | ns | ns |
| 18:1 n-9 | 11.2±0.2 | 10.7±0.03 | 11.6±0.2 | 12.2±0.3 | 11.2±0.5 | 11.5±0.1 | ns | ns | p<0.05 |
| 20:1 | 0.25±0.02 | 0.24±0.03 | 0.30±0.01 | 0.34±0.01 | 0.27±0.03 | 0.26±0.01 | p<0.05 | ns | ns |
| 22:1 | 0.02±0.01 | 0.03±0.01 | 0.04±0.01 | 0.04±0.00 | 0.06±0.02 | 0.03±0.01 | ns | p=0.07 | ns |
| 24:1 | 0.09±0.02 | 0.12±0.02 | 0.14±0.03 | 0.18±0.03 | 0.15±0.04 | 0.12±0.02 | ns | ns | ns |
| **MUFA** | **15.2±0.3** | **14.7±0.03** | **15.7±0.3** | **16.5±0.3** | **15.3±0.6** | **15.5±0.2** | **ns** | **p=0.06** | **p=0.06** |
| 18:2 n-6 (LA) | 0.98±0.03 | 1.14±0.02** | 0.66±0.03***^,###^ | 0.97±0.04 | 1.01±0.04^&^ | 0.67±0.02***^,###^ | p<0.05 | p=0.06 | p<0.001 |
| 20:2 n-6 | 0.14±0.01 | 0.21±0.01*** | 0.10±0.01**^,##^ | 0.15±0.00 | 0.18±0.01 | 0.10±0.01***^,##^ | ns | ns | p<0.001 |
| 20:3 n-6 | 0.53±0.02 | 0.40±0.02*** | 0.41±0.01*** | 0.53±0.01 | 0.37±0.01*** | 0.41±0.01*** | ns | ns | p<0.001 |
| 20:4 n-6 (ARA) | 14.0±0.2 | 14.6±0.2 | 13.9±0.1 | 13.9±0.01 | 14.1±0.3 | 13.7±0.1 | ns | p=0.08 | p<0.05 |
| 22:4 n-6 | 3.39±0.03 | 4.08±0.05*** | 3.31±0.07^###^ | 3.38±0.04 | 3.93±0.08*** | 3.28±0.05^###^ | ns | ns | p<0.001 |
| 22:5 n-6 (DPA) | 0.87±0.02 | 6.99±0.42*** | 0.61±0.15^###^ | 0.92±0.03 | 6.90±0.51*** | 0.71±0.02^###^ | ns | ns | p<0.001 |
| **n-6 LCPUFA** | **20.0±0.2** | **27.5±0.5***** | **19.1±0.1**^###^ | **19.9±0.1** | **26.6±0.8***** | **18.9±0.2**^###^ | **ns** | **ns** | **p<0.001** |
| 18:3 n-3 (ALA) | - | - | - | - | - | - | - | - | - |
| 20:5 n-3 (EPA) | - | - | - | - | - | - | - | - | - |
| 22:5 n-3 (DPA) | 0.41±0.02 | 0.22±0.01*** | 0.26±0.01*** | 0.38±0.01 | 0.21±0.00*** | 0.26±0.01***^, #^ | ns | ns | p<0.001 |
| 22:6 n-3 (DHA) | 16.4±0.3 | 9.7±0.3*** | 17.6±0.2^###^ | 16.0±0.2 | 10.1±0.6*** | 17.8±0.2**^,###^ | **ns** | **ns** | **p<0.001** |
| **n-3 LCPUFA** | **16.9±0.3** | **9.9±0.3***** | **17.8±0.2**^###^ | **16.4±0.2** | **10.3±0.6***** | **18.1±0.2**^,^**^###^ | **ns** | **ns** | **p<0.001** |
| **Total LCPUFA** | **36.8±0.2** | **37.4±0.2** | **36.9±0.2** | **36.3±0.2** | **36.9±0.4** | **37.0±0.1** | **ns** | **ns** | **p=0.07** |
| **n-6/n-3 ratio** | **1.19±0.03** | **2.78±0.12***** | **1.07±0.02**^###^ | **1.21±0.01** | **2.62±0.22***** | **1.05±0.02**^###^ | **ns** | **ns** | **p<0.001** |

SCFA, short-chain saturated fatty acid. MCFA, medium-chain saturated fatty acid. LCFA, long-chain saturated fatty acid. MUFA, monounsaturated fatty acid. LA, linoleic acid. ARA, arachidonic acid. DPA, docosapentaenoic acid. ALA, alpha-linolenic acid. EPA, eicosapentaenoic acid. DHA, docosahexaenoic acid. LCPUFA, long-chain polyunsaturated fatty acid. Values are mean±sem. N=8-10. **p<0.01, **p<0.001 vs. CTL. ^#^p<0.05, ^##^p<0.01, ^###^p<0.001 vs. n-3 DEF. ^&^p<0.05 vs. male. One-way ANOVA with tukey posthoc test.

**Supplemental Table 4.** Average percentage of fatty acid in liver of male and female offspring at PND21-22.

|  | CTL | n-3 DEF | EPA+DHA | CTL | n-3 DEF | EPA+DHA | 2-way Anova | | |
| --- | --- | --- | --- | --- | --- | --- | --- | --- | --- |
|  | *Male* | | | *Female* | | | *Inter* | *Sex* | *Maternal diet* |
| 14:0 | 4.33±0.21 | 3.45±0.24 | 2.77±0.23*** | 4.36±0.24 | 3.29±0.19** | 2.74±0.20*** | ns | ns | **p<0.001** |
| 15:0 | 0.11±0.00 | 0.10±0.00 | 0.13±0.01^###^ | 0.11±0.01 | 0.10±0.00 | 0.14±0.00***^,###^ | ns | ns | **p<0.001** |
| 16:0 | 19.0±0.4 | 20.7±0.4** | 18.1±0.4^###^ | 18.9±0.3 | 20.7±0.4** | 18.0±0.2^###^ | ns | ns | **p<0.001** |
| 18:0 | 6.1±0.2 | 8.4±0.7 | 9.4±0.8** | 6.5±0.3 | 8.6±0.5 | 9.6±0.6** | ns | ns | **p<0.001** |
| 20:0 | 0.04±0.00 | 0.04±0.00 | 0.04±0.00 | 0.04±0.00 | 0.04±0.00 | 0.04±0.00 | ns | ns | ns |
| 22:0 | 0.05±0.00 | 0.07±0.01 | 0.08±0.01 | 0.06±0.00 | 0.07±0.01 | 0.08±0.01 | ns | ns | **p<0.01** |
| 24:0 | 0.15±0.01 | 0.25±0.04 | 0.31±0.03* | 0.16±0.01 | 0.26±0.03 | 0.31±0.03** | ns | ns | **p<0.001** |
| **SCFA** | **7.15±0.38** | **5.18±0.34**** | **4.20±0.28***** | **7.29±0.47** | **4.95±0.34***** | **4.08±0.28***** | **ns** | **ns** | **p<0.001** |
| **MCFA** | **25.2±0.4** | **29.2±0.6***** | **27.7±0.9** | **25.4±0.3** | **29.3±0.6***** | **27.7±0.7** | **ns** | **ns** | **p<0.001** |
| **LCFA** | **0.24±0.01** | **0.37±0.05** | **0.43±0.04*** | **0.26±0.01** | **0.38±0.03** | **0.43±0.03*** | **ns** | **ns** | **p<0.001** |
| **Total SFA** | **32.6±0.5** | **34.7±0.4** | **32.3±0.7** | **33.0±0.4** | **34.7±0.6** | **32.2±0.6^#^** | **ns** | **ns** | **p<0.001** |
| 16:1 n-9 | 0.35±0.03 | 0.25±0.04 | 0.23±0.03 | 0.36±0.04 | 0.24±0.03 | 0.25±0.02 | ns | ns | **p<0.001** |
| 16:1 n-7 | 1.15±0.13 | 1.01±.18 | 0.69±0.10 | 1.24±0.13 | 0.96±0.14 | 0.74±0.09 | ns | ns | **p<0.01** |
| 18:1 n-9 | 31.5±0.7 | 17.4±1.4*** | 21.2±1.5*** | 30.8±0.0.8 | 17.5±1.0*** | 21.5±1.2*** | ns | ns | **p<0.001** |
| 20:1 | 0.58±0.02 | 0.23±0.03*** | 0.35±0.01***^,###^ | 0.56±0.01 | 0.23±0.03*** | 0.35±0.01***^,###^ | ns | ns | **p<0.001** |
| 22:1 | 0.03±0.00 | - | - | 0.03±0.00 | - | - | - | - | - |
| 24:1 | 0.11±0.01 | 0.08±0.01 | 0.18±0.02^##^ | 0.12±0.01 | 0.10±0.01 | 0.18±0.02^##^ | ns | ns | **p<0.001** |
| **MUFA** | **36.1±1.0** | **20.4±1.7***** | **24.6±1.6***** | **35.6±1.1** | **20.6±1.2***** | **25.0±1.3***** | **ns** | **ns** | **p<0.001** |
| 18:2 n-6 (LA) | 11.79±0.29 | 22.3±0.7*** | 9.93±0.33^###^ | 11.82±0.3 | 19.0±1.0*** | 10.04±0.22*^,###^ | ns | ns | **p<0.001** |
| 20:2 n-6 | 0.36±0.02 | 0.77±0.06*** | 0.30±0.01^###^ | 0.3±0.0 | 0.77±0.06*** | 0.29±0.01^###^ | ns | ns | **p<0.001** |
| 20:3 n-6 | 0.85±0.06 | 1.05±.10 | 0.85±0.03 | 0.86±0.06 | 1.01±0.11 | 0.82±0.03 | ns | ns | **p<0.05** |
| 20:4 n-6 (ARA) | 5.5±0.3 | 11.6±1.0*** | 11.3±0.8*** | 5.9±0.4 | 11.5±0.8*** | 11.7±.6*** | ns | ns | **p<0.001** |
| 22:4 n-6 | 0.69±0.08 | 2.46±0.22*** | 1.13±0.06^###^ | 0.63±0.07 | 2.38±0.23*** | 1.05±.05^###^ | ns | ns | **p<0.001** |
| 22:5 n-6 (DPA) | 0.30±0.03 | 3.20±0.32*** | 0.46±0.02^###^ | 0.27±0.01 | 3.22±0.19*** | 0.43±0.02^###^ | ns | ns | **p<0.001** |
| **n-6 LCPUFA** | **19.8±0.6** | **42.1±1.3***** | **24.3±0.5*^,###^** | **20.1±0.7** | **42.1±1.1***** | **24.5±0.5**^,###^** | **ns** | **ns** | **p<0.001** |
| 18:3 n-3 (ALA) | 2.2±0.1 | 0.2±0.0*** | 1.7±.2**^,###^ | 2.2±0.1 | 0.2±.0*** | 1.7±.1**^,###^ | ns | ns | **p<0.001** |
| 20:5 n-3 (EPA) | 0.99±0.04 | 0.06±0.00*** | 0.94±0.05^###^ | 0.98±0.04 | 0.06±0.00*** | 0.94±0.05^###^ | ns | ns | **p<0.001** |
| 22:5 n-3 (DPA) | 2.95±0.22 | 0.58±0.06*** | 2.72±0.19^###^ | 2.75±0.21 | 0.54±0.05*** | 2.53±0.12^###^ | ns | ns | **p<0.001** |
| 22:6 n-3 (DHA) | 4.9±0.4 | 1.8±0.3** | 13.2±0.7***^,###^ | 4.9±0.5 | 1.8±0.2** | 12.9±0.6***^,###^ | ns | ns | **p<0.001** |
| **n-3 LCPUFA** | **11.5±0.6** | **2.7±0.4***** | **18.8±0.7***^,###^** | **11.3±0.7** | **2.6±0.3***** | **18.3±0.6***^,###^** | **ns** | **ns** | **p<0.001** |
| **Total PUFA** | **31.3±0.1.1** | **44.8±0.1.6***** | **43.1±1.0***** | **31.4±1.3** | **44.7±1.3***** | **42.7±1.0***** | **ns** | **ns** | **p<0.001** |
| **n-6/n-3 ratio** | **1.75±.0.05** | **17.15±1.55***** | **1.30±0.04^###^** | **1.80±0.06** | **17.33±1.35***** | **1.35±0.03^###^** | **ns** | **ns** | **p<0.001** |

SCFA, short-chain saturated fatty acid. MCFA, medium-chain saturated fatty acid. LCFA, long-chain saturated fatty acid. MUFA, monounsaturated fatty acid. LA, linoleic acid. ARA, arachidonic acid. DPA, docosapentaenoic acid. ALA, alpha-linolenic acid. EPA, eicosapentaenoic acid. DHA, docosahexaenoic acid. LCPUFA, long-chain polyunsaturated fatty acid. Values are mean±sem. n=8-10. *p<0.05, **p<0.01, **p<0.001 vs. CTL. ^#^p<0.05, ^##^p<0.01, ^###^p<0.001 vs. n-3 DEF. Two-way ANOVA with tukey posthoc test.

**Supplemental Table 5.** Percentage of selected fatty acid in liver of male and female offspring at PND120.

|  | CTL | n-3 DEF | EPA+DHA | CTL | n-3 DEF | EPA+DHA | 2-way Anova | | |
| --- | --- | --- | --- | --- | --- | --- | --- | --- | --- |
|  | *Male* | | | *Female* | | | *Inter* | *Sex* | *Maternal diet* |
| 14:0 | 0.52±0.08 | 0.56±0.07 | 0.45±0.06 | 0.51±0.07 | 0.42±0.06 | 0.44±0.06 | ns | ns | ns |
| 15:0 | 0.25±0.01 | 0.26±0.02 | 0.24±0.02 | 0.15±0.01^&&^ | 0.15±0.01^&&&^ | 0.16±0.01^&&^ | ns | **p<0.001** | ns |
| 16:0 | 23.42±0.68 | 23.32±0.53 | 21.81±1.00 | 20.20±0.60 | 17.80±1.01^&&&^ | 19.02±0.87 | ns | **p<0.001** | ns |
| 18:0 | 12.97±0.68 | 12.56±0.62 | 13.15±1.19 | 20.45±0.95^&&^ | 21.48±1.10^&&&^ | 20.01±1.43^&&^ | ns | **p<0.001** | ns |
| 20:0 | 0.05±0.00 | 0.06±0.00 | 0.07±0.01* | 0.06±0.00 | 0.06±0.00 | 0.06±0.00 | ns | ns | **p<0.05** |
| 22:0 | 0.11±0.01 | 0.10±0.00 | 0.11±0.00 | 0.12±0.01 | 0.12±0.01 | 0.11±0.01 | ns | **p<0.01** | ns |
| 24:0 | 0.36±0.01 | 0.34±0.01 | 0.38±0.02 | 0.38±0.03 | 0.37±0.01 | 0.38±0.02 | ns | ns | ns |
| **SCFA** | **0.54**±0.08 | **0.58**±0.07 | **0.48**±0.05 | **0.54**±0.07 | **0.43**±0.06 | **0.46**±0.06 | ns | ns | ns |
| **MCFA** | **36.65**±0.50 | **36.14**±0.32 | **35.20**±0.61 | **40.80**±1.01^&^ | **39.43**±0.43^&^ | **39.19**±1.02^&&^ | ns | **p<0.001** | ns |
| **LCFA** | **0.53**±0.01 | **0.50**±0.01 | **0.55**±0.03 | **0.56**±0.04 | **0.55**±0.0 | **0.56**±0.03 | ns | ns | ns |
| **Total SFA** | **37.71**±0.50 | **37.21**±0.33 | **36.24**±0.64 | **41.90**±1.08^&^ | **40.41**±0.43 | **40.20**±1.06^&^ | ns | **p<0.001** | ns |
| 16:1 n-9 | 0.18±0.02 | 0.22±0.02 | 0.19±0.01 | 0.22±0.01 | 0.21±0.03 | 0.22±0.02 | ns | ns | ns |
| 16:1 n-7 | 2.62±0.64 | 2.75±0.33 | 2.33±0.28 | 1.74±0.28 | 1.45±0.36 | 1.66±0.18 | ns | **p<0.01** | ns |
| 18:1 n-9 | 7.81±0.73 | 8.57±0.37 | 8.25±0.68 | 11.13±1.25 | 10.93±1.05 | 10.84±0.78 | ns | **p<0.001** | ns |
| 20:1 | 0.19±.01 | 0.20±0.01 | 0.20±0.01 | 0.14±0.01^&&^ | 0.16±0.00^&^ | 0.15±0.01^&&^ | ns | **p<0.001** | ns |
| 22:1 | 0.04±0.01 | 0.04±0.00 | 0.05±0.01 | 0.05±0.01 | 0.05±0.01 | 0.04±0.01 | ns | ns | ns |
| 24:1 | 0.18±0.01 | 0.18±0.01 | 0.18±0.00 | 0.19±0.02 | 0.19±0.01 | 0.17±0.01 | ns | ns | ns |
| **MUFA** | **16.40**±1.92 | **17.83**±0.85 | **17.16**±0.56 | **15.97**±1.57 | **15.72**±1.58 | **16.13**±0.74 | ns | ns | ns |
| 18:2 n-6 (LA) | 16.11±0.64 | 15.61±0.26 | 16.57±0.20 | 13.70±0.83 | 13.70±0.52 | 13.27±1.07^&^ | ns | **p<0.001** | ns |
| 20:2 n-6 | 0.45±0.02 | 0.42±0.02 | 0.46±0.03 | 0.23±0.01^&&&^ | 0.25±0.01^&&&^ | 0.25±0.03^&&&^ | ns | **p<0.001** | ns |
| 20:3 n-6 | 1.00±0.11 | 0.86±0.05 | 0.92±0.08 | 0.85±0.06 | 0.81±0.05 | 0.85±0.07 | ns | ns | ns |
| 20:4 n-6 (AA) | 18.23±1.32 | 18.19±0.92 | 18.02±0.34 | 16.75±0.82 | 18.61±1.05 | 18.17±0.79 | ns | ns | ns |
| 22:4 n-6 | 0.42±0.03 | 0.54±0.03* | 0.49±0.03 | 0.36±0.01 | 0.43±0.02^&^ | 0.36±0.01^&&^ | ns | **p<0.001** | **p<0.01** |
| 22:5 n-6 (DPA) | 0.24±0.00 | 0.30±0.03 | 0.27±0.01 | 0.26±0.03 | 0.28±0.03 | 0.27±0.02 | ns | ns | ns |
| **n-6 LCPUFA** | **36.57**±1.69 | **36.06**±0.78 | **36.86**±0.45 | **32.35**±1.10 | **34.25**±1.33 | **33.33**±1.57 | ns | **p<0.01** | ns |
| 18:3 n-3 (ALA) | 0.30±0.01 | 0.32±0.02 | 0.35±0.04 | 0.32±0.04 | 0.33±0.03 | 0.28±0.03 | ns | ns | ns |
| 20:5 n-3 (EPA) | 0.48±0.03 | 0.46±0.03 | 0.51±.03 | 0.68±0.07^&&^ | 0.49±0.04* | 0.61±0.04 | ns | **p<0.01** | ns |
| 22:5 n-3 (DPA) | 1.19±0.07 | 1.17±0.07 | 1.18±0.10 | 0.90±0.06 | 0.79±0.04^&&^ | 0.86±0.04^&^ | ns | **p<0.001** | ns |
| 22:6 n-3 (DHA) | 7.32±0.18 | 6.92±0.29 | 7.67±0.32 | 7.86±0.33 | 7.98±0.39 | 8.54±0.36 | ns | **p<0.01** | ns |
| **n-3 LCPUFA** | **9.33**±0.12 | **8.90**±0.27 | **9.75**±0.35 | **9.78**±0.30 | **9.62**±0.32 | **10.33**±0.34 | ns | **p<0.05** | **p<0.05** |
| **Total LCPUFA** | **45.89**±1.73 | **44.97**±0.92 | **46.61**±0.77 | **42.13**±1.17 | **43.87**±1.61 | **43.66**±1.59 | ns | **p<0.05** | ns |
| **n-6/n-3 ratio** | **3.92**±0.17 | **4.06**±0.12 | **3.79**±0.10 | **3.32**±0.15 | **3.56**±0.08 | **3.24**±0.20 | ns | **p<0.001** | ns |

SCFA, short-chain saturated fatty acid. MCFA, medium-chain saturated fatty acid. LCFA, long-chain saturated fatty acid. MUFA, monounsaturated fatty acid. LA, linoleic acid. AA, arachidonic acid. DPA, docosapentaenoic acid. ALA, alpha-linolenic acid. EPA, eicosapentaenoic acid. DHA, docosahexaenoic acid. LCPUFA, long-chain polyunsaturated fatty acid. Values are mean±sem. N=4-6. *p<0.05 vs. CTL. ^&^p<0.05, ^&&^p<0.01, ^&&&^p<0.001 vs. male. Two-way ANOVA with tukey posthoc test.

**Supplemental Table 6.** Percentage of selected fatty acid in liver of male and female offspring at PND120 after HFD.

|  | CTL | n-3 DEF | EPA+DHA | CTL | n-3 DEF | EPA+DHA | 2-way Anova | | |
| --- | --- | --- | --- | --- | --- | --- | --- | --- | --- |
|  | *Male* | | | *Female* | | | *Inter* | *Sex* | *Maternal diet* |
| 14:0 | 0.35±0.06 | 0.45±0.05 | 0.29±0.05 | 0.25±0.04 | 0.34±0.01 | 0.34±0.06 | ns | ns | ns |
| 15:0 | 0.11±0.00 | 0.13±0.01 | 0.11±0.01 | 0.09±0.00 | 0.09±0.00^&&^ | 0.09±.00 | ns | **p<0.001** | ns |
| 16:0 | 21.78±0.28 | 22.76±1.01 | 20.28±0.89 | 18.11±0.98 | 18.71±0.42^&^ | 18.40±0.63 | ns | **p<0.001** | ns |
| 18:0 | 10.70±1.34 | 9.12±0.71 | 14.39±2.12 | 20.03±1.65^&&^ | 18.81±0.91^&&^ | 19.16±1.17 | ns | **p<0.001** | ns |
| 20:0 | 0.05±0.00 | 0.05±0.00 | 0.06±0.00 | 0.06±0.00 | 0.06±0.00 | 0.06±0.00 | ns | **p<0.01** | ns |
| 22:0 | 0.07±0.01 | 0.06±0.01 | 0.09±0.02 | 0.13±0.01^&^ | 0.12±0.01^&&^ | 0.12±0.01 | ns | **p<0.001** | ns |
| 24:0 | 0.15±0.02 | 0.13±0.01 | 0.22±0.03 | 0.30±0.02^&&^ | 0.26±0.02^&&^ | 0.26±.01 | **p<0.05** | **p<0.001** | ns |
| **SCFA** | **0.37**±0.06 | **0.46**±0.05 | **0.31**±0.05 | **0.27**±0.04 | **0.35**±0.01 | **0.45**±0.16 | ns | ns | ns |
| **MCFA** | **32.60**±1.21 | **32.02**±0.86 | **34.77**±1.35 | **38.23**±0.67^&^ | **37.60**±0.51^&&^ | **37.65**±0.61 | ns | **p<0.001** | ns |
| **LCFA** | **0.27**±0.04 | **0.23**±0.02 | **0.37**±0.05 | **0.49**±0.04^&&^ | **0.43**±0.03^&&^ | **0.44**±0.02 | ns | **p<0.001** | ns |
| **Total SFA** | **33.23**±1.19 | **32.71**±0.90 | **35.45**±1.36 | **38.98**±0.67^&&^ | **38**.39±0.54^&&^ | **38.54**±0.49 | ns | **p<0.001** | ns |
| 16:1 n-9 | 0.30±0.06 | 0.35±0.04 | 0.23±0.04 | 0.21±.03 | 0.24±0.01 | 0.22±0.01 | ns | **p<0.01** | ns |
| 16:1 n-7 | 1.05±0.25 | 1.30±0.16 | 0.91±0.23 | 0.60±0.12 | 0.83±0.06 | 0.71±0.08 | ns | **p<0.01** | ns |
| 18:1 n-9 | 28.10±2.74 | 33.35±2.44 | 23.79±3.00 | 19.72±2.29 | 21.36±0.81^&^ | 20.10±1.01 | ns | **p<0.001** | ns |
| 20:1 | 0.40±0.05 | 0.50±0.07 | 0.32±0.04 | 0.20±0.01^&^ | 0.22±.01^&&&^ | 0.22±0.02 | ns | **p<0.001** | ns |
| 22:1 | 0.05±0.01 | 0.04±0.01 | 0.04±0.01 | 0.05±0.00 | 0.04±0.00 | 0.03±0.00 | ns | ns | ns |
| 24:1 | 0.08±0.01 | 0.06±0.01 | 0.09±0.01 | 0.13±0.02 | 0.11±0.01 | 0.11±0.01 | ns | **p<0.001** | ns |
| **MUFA** | **32.76**±3.24 | **38.76**±2.69* | **27.85**±3.48 | **22.87**±2.48 | **24.92**±0.86^&&^ | **23.55**±1.16 | ns | **p<0.001** | ns |
| 18:2 n-6 (LA) | 15.60±0.069 | 12.12±3.10 | 13.78±0.81 | 11.74±0.65 | 11.52±.69 | 11.24±0.49 | ns | **p<0.05** | ns |
| 20:2 n-6 | 0.26±0.03 | 0.30±0.05 | 0.22±0.01 | 0.19±0.01 | 0.20±0.01 | 0.19±0.01 | ns | **p<0.01** | ns |
| 20:3 n-6 | 0.48±0.05 | 0.55±0.07 | 0.57±0.06 | 0.55±0.04 | 0.55±0.04 | 0.55±0.02 | ns | ns | ns |
| 20:4 n-6 (AA) | 12.05±1.80 | 10.53±0.99 | 15.77±1.98 | 16.99±1.52 | 16.05±.61 | 17.31±0.84 | ns | **p<0.01** | ns |
| 22:4 n-6 | 0.47±0.04 | 0.50±0.08 | 0.40±0.03 | 0.33±0.02 | 0.36±0.03 | 0.36±0.02 | ns | **p<0.01** | ns |
| 22:5 n-6 (DPA) | 0.36±0.11 | 0.36±0.05 | 0.46±0.12 | 0.57±0.14 | 0.50±0.05 | 0.54±0.05 | ns | ns | ns |
| **n-6 LCPUFA** | **29.44**±1.49 | **24.62**±3.37 | **31.42**±1.69 | **30.59**±1.06 | **29.35**±0.08 | **30.37**±0.61 | ns | ns | ns |
| 18:3 n-3 (ALA) | 0.51±0.08 | 0.57±0.11* | 0.33±0.06 | 0.32±0.05 | 0.35±0.04^&^ | 0.30±0.03 | ns | **p<0.01** | ns |
| 20:5 n-3 (EPA) | 0.10±0.01 | 0.12±0.03 | 0.11±.01 | 0.12±0.02 | 0.13±0.01 | 0.11±0.01 | ns | ns | ns |
| 22:5 n-3 (DPA) | 0.49±0.04 | 0.42±0.08 | 0.44±0.06 | 0.39±0.02 | 0.41±0.06 | 0.43±0.03 | ns | ns | ns |
| 22:6 n-3 (DHA) | 3.45±0.65 | 2.75±0.15 | 4.37±0.79 | 6.70±0.88^&^ | 6.44±0.49^&&^ | 6.66±0.55 | ns | **p<0.001** | ns |
| **n-3 LCPUFA** | **4.57**±0.61 | **3.92**±0.31 | **5.28**±0.75 | **7.56**±0.80^&^ | **7.35**±0.40^&&^ | **7.54**±0.52 | ns | **p<0.001** | ns |
| **Total LCPUFA** | **34.01**±2.07 | **28.53**±3.25 | **36.70**±2.19 | **38.15**±1.82 | **36.69**±0.35 | **37.91**±0.82 | ns | **p<0.01** | ns |
| **n-6/n-3 ratio** | **6.69**±0.47 | **6.52**±0.99 | **6.41**±0.65 | **4.15**±0.33 | **4.04**±0.23 | **4.16**±0.34^&^ | ns | **p<0.001** | ns |

SCFA, short-chain saturated fatty acid. MCFA, medium-chain saturated fatty acid. LCFA, long-chain saturated fatty acid. MUFA, monounsaturated fatty acid. LA, linoleic acid. AA, arachidonic acid. DPA, docosapentaenoic acid. ALA, alpha-linolenic acid. EPA, eicosapentaenoic acid. DHA, docosahexaenoic acid. LCPUFA, long-chain polyunsaturated fatty acid. Values are mean±sem. N=4-7. *p<0.05 vs. CTL. ^&^p<0.05, ^&&^p<0.01 vs. male. Two-way ANOVA with tukey posthoc test.

**Supplemental Table 7.** List of primers used for qPCR.

| Gene | Name | Forward primer | Reverse primer |
| --- | --- | --- | --- |
| Acaca | Acetyl CoA carboxylase alpha | TGAAGCCAAGATAATCCAGCA | CATATCTTTCATCCCACCAGAG |
| Acsl1 | Acyl CoA synthetase long chain | TCACAAGATACAGTCGAGCCT | CATAGAACTGACAGCCGAGAG |
| Beta-actin | Beta-actin | CTATCGGCAATGAGCGGTTCC | GCACTGTGTTGGCATAGAGGTC |
| CD36 | - | TTGCAGGTCCATCTATGCTGTG | AAGGCGTTGGCTGGAAGAAC |
| Cpt1a | Carnitine palmitoyl transferase 1 | TGCCTGCCAGTTCCATTAAGC | GTCTCACTCCTCTTGCCAACAG |
| Dgat2 | Diacylglycerol O-acyltransferase 2 | GGTCATCTCAGTCCTACAG | CTATCAGCCAGCAGTCAG |
| Elovl2 | Elongation of very long chain fatty acids protein 2 | GACCAGTCAGATCACCTTCCT | GTCCGAAGAAGCTTTGACCAC |
| Elovl5 | Elongation of very long chain fatty acids protein 5 | CCTGGTGGTGTATAATCTTGG | GGACACGAATAACCTTCATATCTG |
| Elovl6 | Elongation of very long chain fatty acids protein 6 | CTTGCGGTCTTCAGTATATTCGG | AAACTCTGGTCACAAACTGACTG |
| Fabp4 | Fatty acid binding protein 4 | TGTGGGGACCTGGAAACTCGT | CGAAGCCAACTCCCACTTCTTT |
| Fads1 | Fatty acid desaturase 1 | CAGATTGAACACCACCTCTTCC | TGGACTCATACTTGATGCCGT |
| Fads2 | Fatty acid desaturase 2 | GATTGAGCACCACCTCTTCC | TGAACTCACAATGTCGAGCAG |
| Fas | Fatty acid synthase | CGCCGTGGTGCTGGAGATTG | CTTGCCGAGGTTGGTGAGGAAG |
| Hmgcr | Hydroxymethylglutaryl-CoA reductase | TGTTCACCGGCAACAACAAGA | GCTCAGCACGTCCTCTTCAAA |
| Ldlr | Low density lipoprotein receptor | CAGGATAAGTACGCCATCGG | GTCTGGTTTACCTTTCACTGAG |
| Lpl | Lipoprotein lipase | AACTGCCACTTCAACCACAGC | CATACATTCCTGTCACCGTCCA |
| LRP | LDL receptor-related protein 1 | ACTATTTATTGGGTGGACATGGG | GTAGATGTTGCCTGCGATCC |
| Lxra | Nuclear receptor subfamily 1, group H, member 3 | CAGAGCCTACAGAACTTCGT | CAGCTCAGCACATTGTAATGG |
| Mttp | Microsomal triglyceride transfer protein | ATAGTGTCGAAGCAGAAATTGG | GGGAATGGCTTTGTACTTGGA |
| Ppara | Peroxisome proliferator-activated receptor alpha | CACGATGCTGTCCTCCTTGATG | ATGATGTCGCAGAATGGCTTCC |
| Pparg | Peroxisome proliferator-activated receptor gamma | CCGTTCACAAGAGCTGACCC | TCGCACTTTGGTATTCTTGGAGC |
| Scd1 | Stearoyl-coa desaturase | GTTGGGTGCCTTATCGCTTTCC | CTCCAGCCAGCCTCTTGTCTAC |
| Soat2 | Sterol O-acyltransferase 2 | GATTCTACAAGGAAGACCCAAGAG | CTGCTATGAACATGTGGTAGATGG |
| Srebf1 | Sterol regulatory element binding factor 1 | CCG TGG TGG TGA TGG TGG TAT C | TGC TGT GCG TCC TGT GTT CC |
